# New Anticancer Theobromine Derivative Targeting EGFR^WT^ and EGFR^T790M^: Design, Semi-Synthesis, In Silico, and In Vitro Anticancer Studies

**DOI:** 10.3390/molecules27185859

**Published:** 2022-09-09

**Authors:** Eslam B. Elkaeed, Reda G. Yousef, Hazem Elkady, Aisha A. Alsfouk, Dalal Z. Husein, Ibrahim M. Ibrahim, Ahmed M. Metwaly, Ibrahim H. Eissa

**Affiliations:** 1 Department of Pharmaceutical Sciences, College of Pharmacy, AlMaarefa University, Riyadh 13713, Saudi Arabia; 2 Pharmaceutical Medicinal Chemistry & Drug Design Department, Faculty of Pharmacy (Boys), Al-Azhar University, Cairo 11884, Egypt; 3 Department of Pharmaceutical Sciences, College of Pharmacy, Princess Nourah bint Abdulrahman University, Riyadh 11671, Saudi Arabia; 4 Chemistry Department, Faculty of Science, New Valley University, El-Kharja 72511, Egypt; 5 Biophysics Department, Faculty of Science, Cairo University, Cairo 12613, Egypt; 6 Pharmacognosy and Medicinal Plants Department, Faculty of Pharmacy (Boys), Al-Azhar University, Cairo 11884, Egypt; 7 Biopharmaceutical Products Research Department, Genetic Engineering and Biotechnology Research Institute, City of Scientific Research and Technological Applications (SRTA-City), Alexandria 21934, Egypt

**Keywords:** anticancer, theobromine derivative, semi-synthesis, EGFR, Molecular docking, MD simulations, DFT

## Abstract

Based on the pharmacophoric features of EGFR inhibitors, a new semisynthetic theobromine-derived compound was designed to interact with the catalytic pocket of EGFR. Molecular docking against wild (EGFR^WT^; PDB: 4HJO) and mutant (EGFR^T790M^; PDB: 3W2O) types of EGFR-TK indicated that the designed theobromine derivative had the potential to bind to that pocket as an antiangiogenic inhibitor. The MD and MM-GBSA experiments identified the exact binding with optimum energy and dynamics. Additionally, the DFT calculations studied electrostatic potential, stability, and total electron density of the designed theobromine derivative. Both in silico ADMET and toxicity analyses demonstrated its general likeness and safety. We synthesized the designed theobromine derivative (compound **XI**) which showed an IC_50_ value of 17.23 nM for EGFR inhibition besides IC_50_ values of 21.99 and 22.02 µM for its cytotoxicity against A549 and HCT-116 cell lines, respectively. Interestingly, compound **XI** expressed a weak cytotoxic potential against the healthy W138 cell line (IC_50_ = 49.44 µM, 1.6 times safer than erlotinib), exhibiting the high selectivity index of 2.2. Compound **XI** arrested the growth of A549 at the G2/M stage and increased the incidence of apoptosis.

## 1. Introduction

World Health Organization, WHO, statistics indicate that cancer will become the major cause of death within a few years [1]. In response, developing safe and effective cancer therapies that bind with a specific molecular target and destroy cancer cells represents a challenging problem for scientists [2]. Angiogenesis contributes to tumor growth and reproduction. Hence, stopping angiogenesis is considered one of the possible strategies to treat cancer [3]. Angiogenesis and cancer cell growth depend greatly on the epidermal growth factor receptor (EGFR) [4]. Overexpression of EGFR promotes cellular proliferation, differentiation, and survival through downstream signaling pathways. The EGFR gene promotes the growth of various solid tumors and is elevated in multiple types of cancer [5]. Increased EGFR expression was proven to be associated with a reduced survival rate, and acted as a strong prognostic indicator [6]. EGFR receptors are overexpressed in cancer more than in normal cells, allowing researchers to target them as a cancer-fighting strategy [7].

Natural products have historically been the cornerstone of drug development [8,9]. Recently, natural products accounted for almost one-third of newly approved FDA drugs in the period 1981–2014 [10]. Xanthines, as well as xanthine derivatives, are an extremely interesting category of compounds in the field of anticancer drug discovery as they expressed various antimutagenic activities against different cancer types such as ovarian [11], prostate [12], leukemia [13], and breast [13] cancers. First discovered in 1841 [14], theobromine is a xanthine alkaloid (3,7-dimethylxanthine) found primarily in *Theobroma cacao*, “chocolate”, as well as in a variety of foods such as tea leaves [15]. It was first synthesized in 1882 [16]. Interestingly, theobromine expressed promising activities against colon cancer through in vitro [17] as well as in vivo [18] examinations. Additionally, theobromine showed anti-carcinogenic activity by inhibiting the DNA synthesis in cancer cells [19]. Further, theobromine inhibited the growth of glioblastoma multiforme in vitro [20]. Remarkably, theobromine could prevent angiogenesis in lung cancer in an in vivo study [21] as well as it exhibits promising anti-angiogenic activity via the inhibition of the vascular endothelial growth factor (VEGF), in vivo and in vitro, in ovarian cancer [22]. 

The semi-synthesis of natural products can aid in discovering more potent drugs and repurposing, as well as improving drug-likeness, pharmacokinetics, and pharmacodynamics [23].

Computational (computer-based, computer-aided) chemistry is used to explore the interactions of potential drugs with biomolecules using theoretical ideas and computer techniques [24]. Our team applied in molecular docking [25,26], molecular design [27], toxicity [28,29], ADMET [30,31], DFT [32,33], structural similarity [34], MD [35], and pharmacophore [36] evaluation.

**Figure 1:**
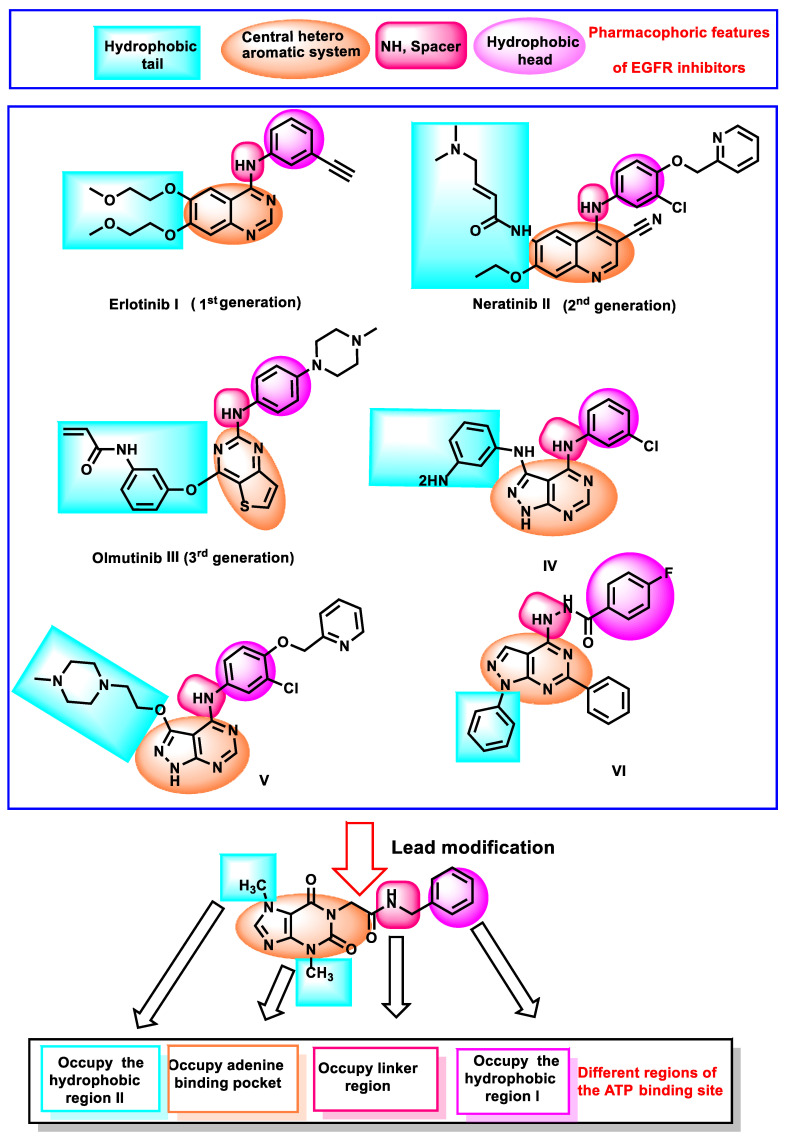
The design rationale of the targeted compound.

The clinically approved EGFR tyrosine kinase-inhibitors (EGFR TKIs) (Figure 1) have some problems. The anticancer effect of the 1st-generation EGFR-TKI as erlotinib **I** [37] was decreased due to the drug resistance acquired by a certain mutation (EGFR^T790M^) [38]. In addition, erlotinib was reported to exert life-threatening lung disease [39]. Although overcoming the drug resistance induced by EGFR^T790M^, the 2nd-generation EGFR-TKIs (as neratinib **II** [40]) showed poor clinical patient outcomes due to a low maximal-tolerated-dose [41,42]. Olmutinib **III**, the 3rd-generation EGFR-TKIs, showed improved activities towards EGFR^T790M^. Unfortunately, two cases of toxic epidermal necrolysis (one of them was a fatal case) and a case of Stevens–Johnson Syndrome were recorded by Olmutinib [43]. Compound **IV** was generated by Traxler et al. [44] and showed excellent efficacy in inhibiting EGFR TK. Compound **V** is also an example of 1*H*-pyrazolo[3,4-d]pyrimidines with anti-EGFR TK activity, have a low IC_50_ value against the BT474C cell line [45] (Figure 1). In 2018, our research group synthesized a series of 1*H*-pyrazolo[3,4-*d*]pyrimidine analogues as inhibitors of EGFR^WT^ and EGFR^T790M^. Compound **VI** showed good inhibitory effects against the two types of EGFR besides a good apoptotic effect [46].

### Rationale

The ATP-binding active pocket of the EGFR comprises five essential regions including the adenine and ribose binding pockets, in addition to the phosphate binding region, hydrophobic regions I and II. The ribose binding pocket and phosphate binding region are not essential for EGFR-TKIs intrinsic activity [47,48,49,50].

EGFR-TKIs have four common pharmacophoric features [51]: (**i**) A flat hetero aromatic system, terminal hydrophobic head, NH spacer, and hydrophobic tail. These moieties occupy and interact with the adenine binding pocket [52], the hydrophobic region I [51], linker region [53], and hydrophobic region II [47,54], respectively.

As shown in Figure 1, different moieties can occupy the adenine binding pocket as quinazoline (erlotinib **I**), quinoline (neratinib **II**), thieno[3,2-d]pyrimidine (olmutinib **III**), 1*H*-pyrazolo[3,4-d]pyrimidine (compounds **IV, V, VI**).

In continuation of our efforts in the discovery of EGFR-2 inhibitors [46,55,56,57], we designed and synthesized a new theobromine derivative as a potential compound that may exert a marked EGFR inhibitory activity and consequently inhibit the growth of tumor cells. The designed compound is a modified analog of 1*H*-pyrazolo[3,4-d]pyrimidine derivatives.

From a drug design point of view, the theobromine moiety was utilized in the current work to engage the adenine binding pocket. This moiety has six hydrogen acceptor atoms that can bind efficiently with the essential amino acid at the adenine binding pocket. In addition, the acetamide moiety was used as a linker. Furthermore, the hydrophobic head is to be inserted in the hydrophobic region I. Lastly, one of the two methyl groups at the 3- and 7-positions of theobromine moiety was used proposed to occupy the hydrophobic region II (Figure 1). The binding mode of the synthesized compound confirmed the design as each feature occupied its target pocket in the ATP binding site.

## 2. Results and Discussion

### 2.1. In Silico Studies

#### 2.1.1. Molecular Docking against Wild and Mutant EGFR

The molecular modeling tool allowed medicinal chemists to envisage interactions between compounds and their biological target [58,59,60]. The designed theobromine derivative’s binding mode against wild (EGFR^WT^; PDB: 4HJO) and mutant (EGFR^T790M^; PDB: 3W2O) types of EGFR-TK, [61,62]. The co-crystallized ligands, erlotinib, and TAK-285, of wild and mutant types, respectively, were utilized as references.

**Figure 2:**
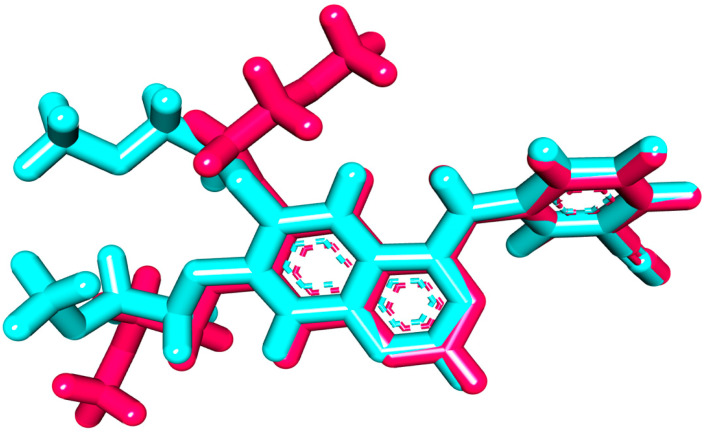
Validation step of erlotinib.

**Figure 3:**
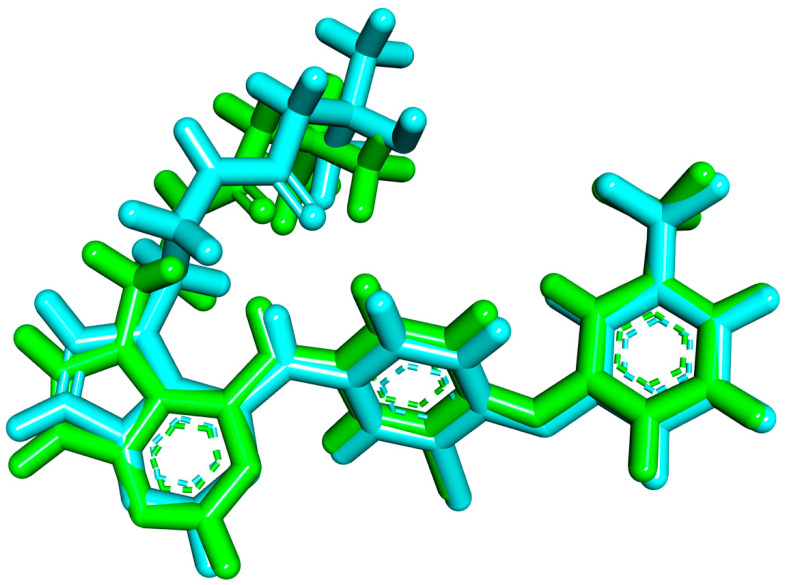
Validation step of TAK-285.

The re-docking validation step of the co-crystallized ligands (erlotinib and TAK-285) showed acceptable root–mean–square deviation, RMSD, values of (1.40 and 1.10 Å, respectively), as presented in Figure 2 and Figure 3.

**Figure 4:**
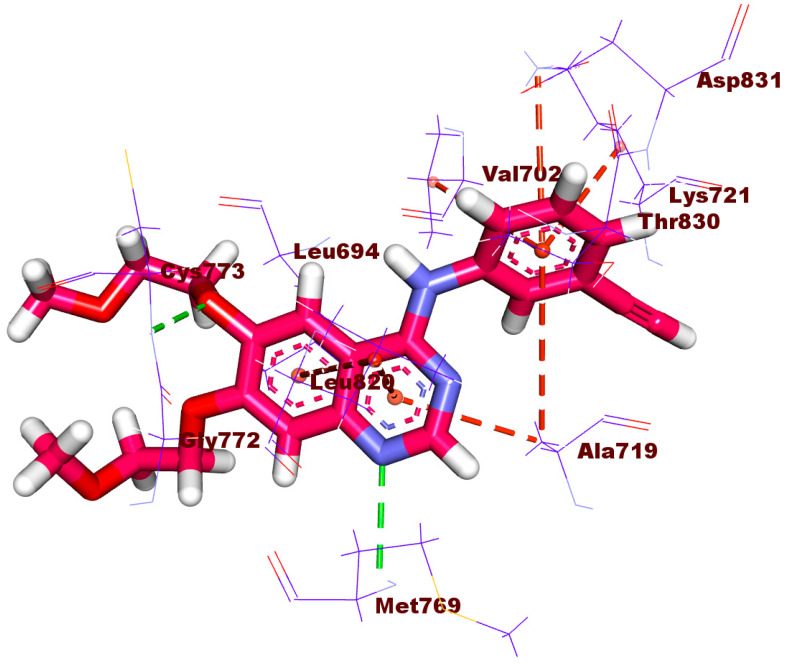

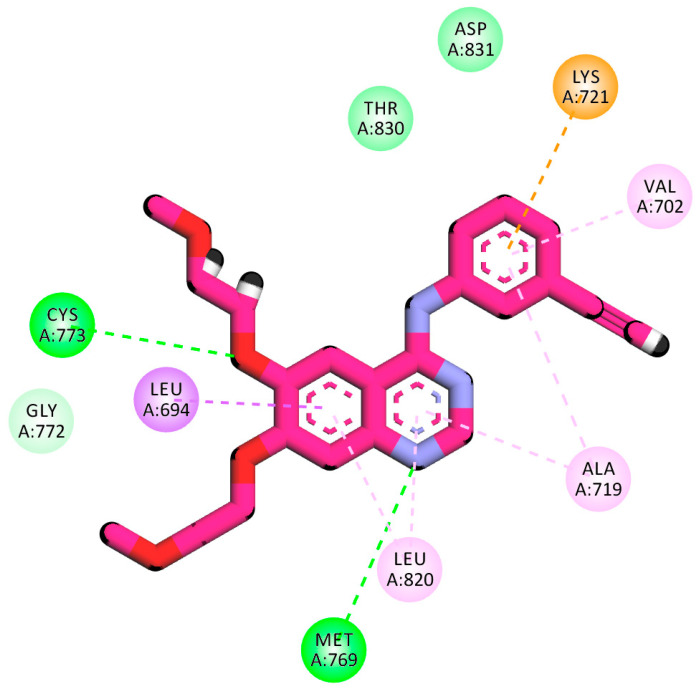
3D and 2D binding mode of erlotinib into the active site of EGFR^WT^.

Erlotinib’s binding interactions with EGFR^WT^ revealed that it occupied the major pockets (affinity value of −20.35 kcal/mol). A key hydrogen bond (H-B) with Met769, besides four hydrophobic interactions (H-I) with Leu694, Ala719, and Leu820, was observed in the adenine pocket. This was achieved via the quinazoline moiety. Moreover, the hydrophobic pocket I was occupied by the ethynylphenyl moiety through a network of H-Is with Ala719 and Val702, and Lys721 (Figure 4).

**Figure 5:**
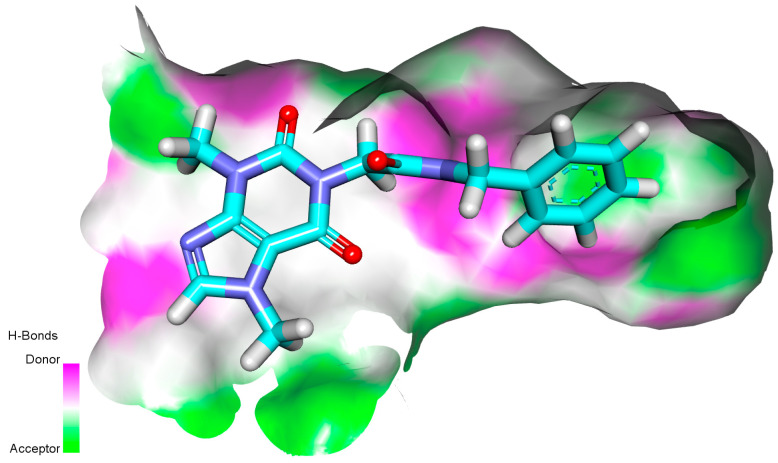

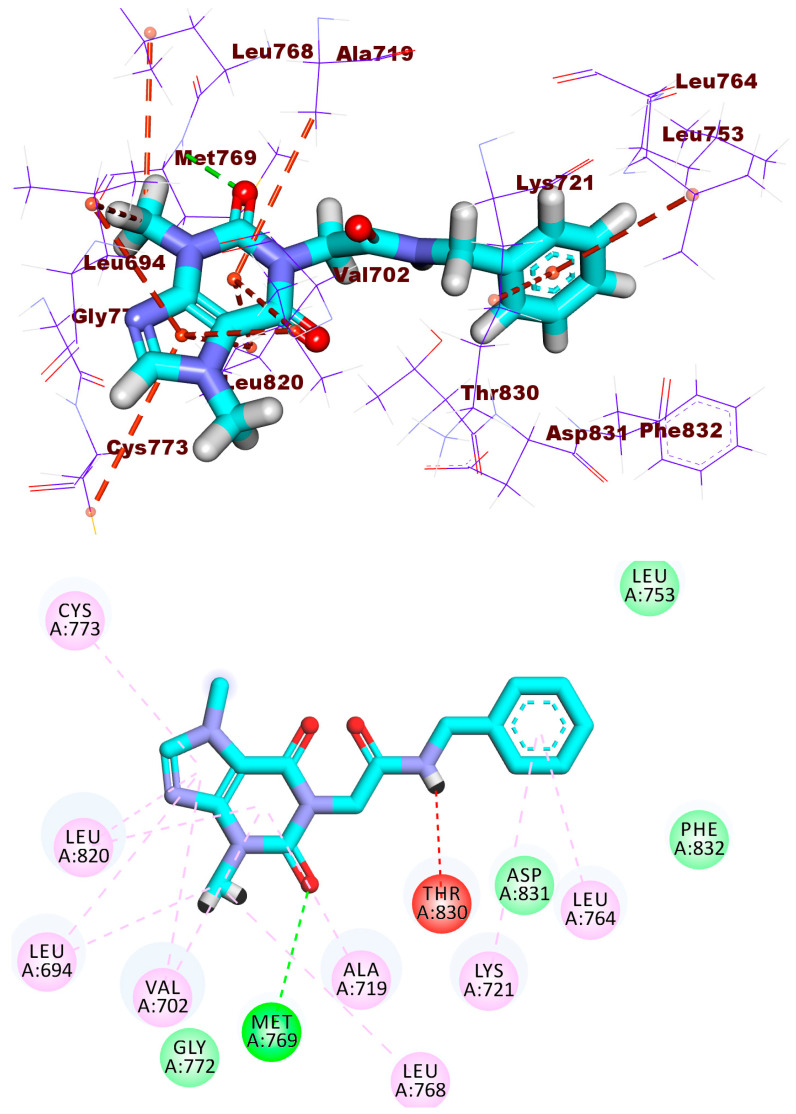
MS, 3D, and 2D binding pattern of the designed theobromine derivative with the active site of EGFR^WT^.

The designed theobromine derivative gave a comparable affinity value to erlotinib (−20.11 kcal/mol). In addition, it took the same orientation and interacted with EGFR^WT^ active site similar to erlotinib. The 7-methyl-2,6-dioxo-2,3,6,7-tetrahydro-1*H*-purine of the designed compound was oriented into the adenine pocket of the EGFR^WT^ with a H-B against the essential amino acid, Met769, and seven H-Is with Cys733, Leu820, Leu694, Leu786, Ala719, and Val 702. The NH group of the acetamide moiety was incorporated in an electrostatic attraction with Thr830. On the other side, the benzyl moiety occupied the hydrophobic pocket I via two H-Is with Leu764 and Lys721. The methyl group at the 4-position of xanthine moiety was oriented into the hydrophobic pocket II with two H-Is against Leu694 and Val 702 (Figure 5).

**Figure 6:**
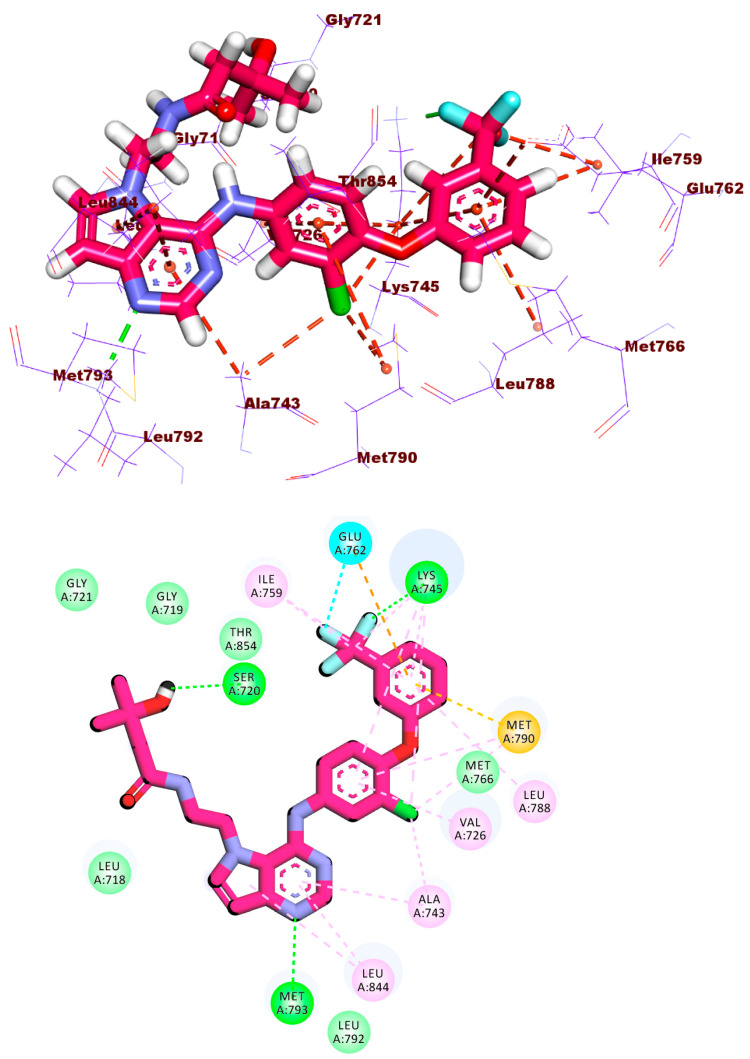
TAK-285 docked into EGFR^T790M^ active site.

The docking outputs of the mutant EGFR (EGFR^T790M^) were investigated to support the docking results of the wild type (EGFR^WT^). Figure 6 explains the binding of TAK-285, the co-crystallized ligand, (TAK-285) to the EGFR^T790M^ active site. The obtained findings and the reported data were identical [55].

**Figure 7:**
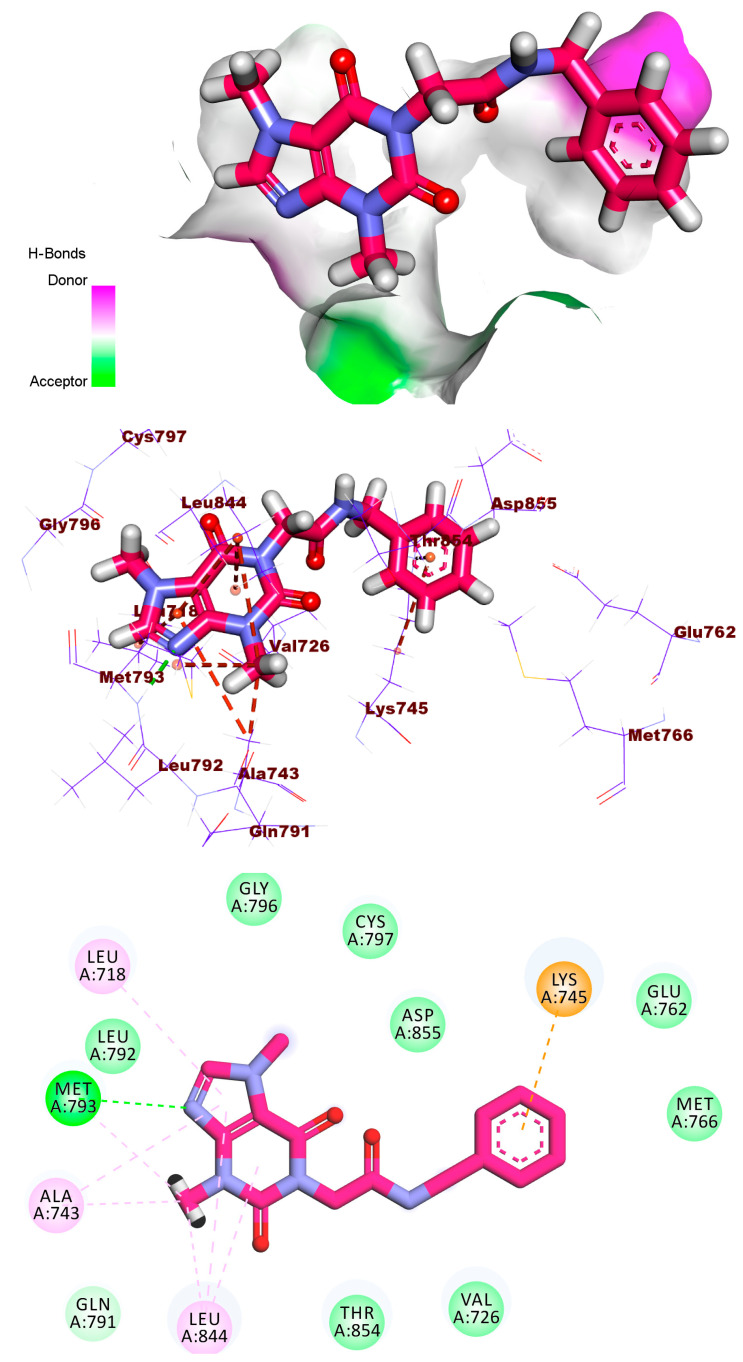
Binding of the designed theobromine derivative with EGFR^T790M^.

As displayed in Figure 7, the designed theobromine derivative was bound to the catalytic site of the EGFR^T790M^ in similar to TAK-285. The 7-methyl-2,6-dioxo-2,3,6,7-tetrahydro-1*H*-purine moiety was successfully buried in the adenine pocket to form one H-B with Met793 and four p-p bonds with Leu844, Leu718, Ala743, and Met793. Moreover, the terminal benzyl moiety interacted with the hydrophobic pocket I, forming an electrostatic interaction with Lys745. The methyl group at the 4-position of xanthine moiety was oriented into the hydrophobic pocket II, forming three H-Is with Leu844, Ala743, and Met793.

#### 2.1.2. MD Simulations

**Figure 8:**
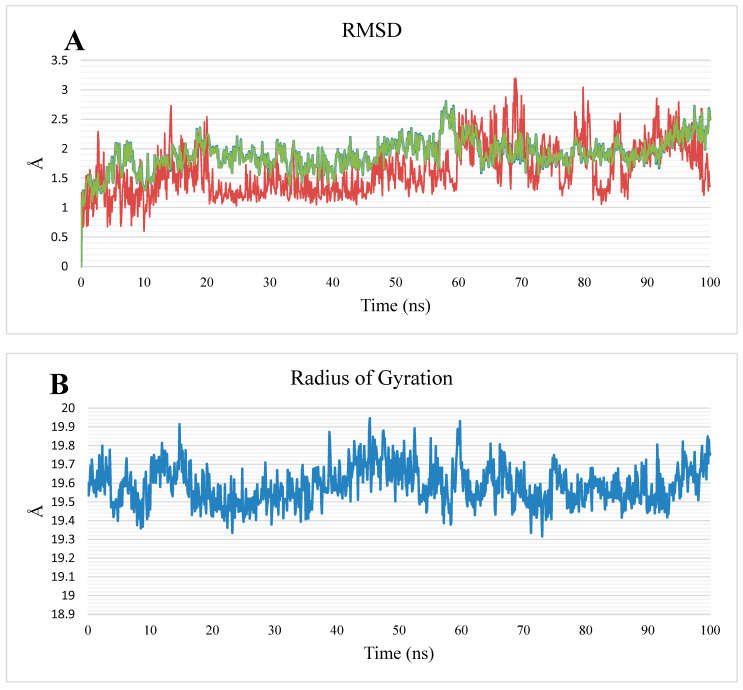

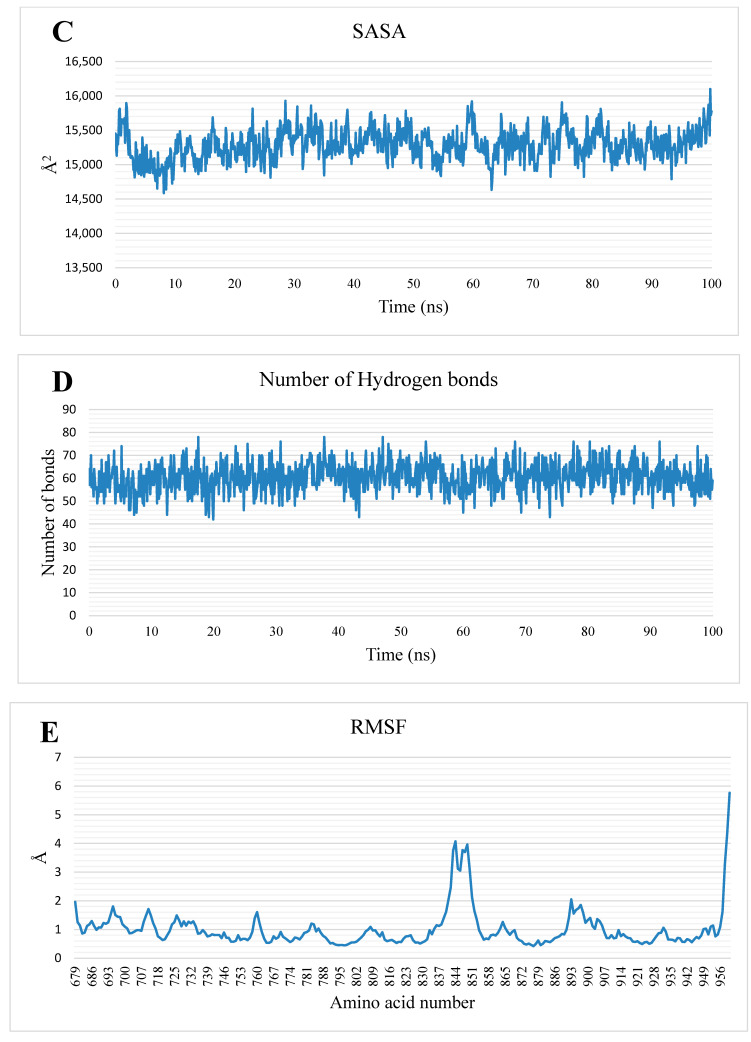

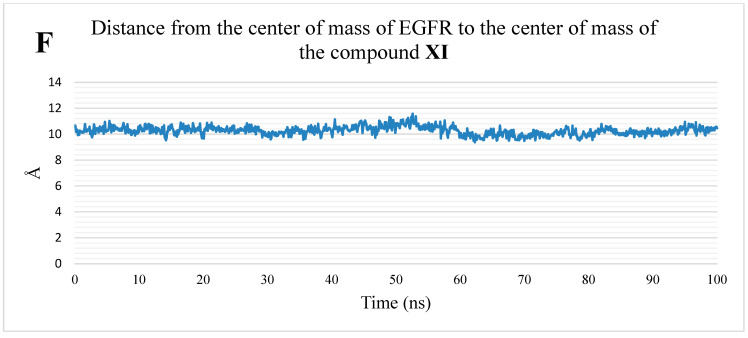
Different measurements produced from the trajectory. (**A**) RMSD: EGFR is blue, the designed theobromine derivative is red, and the complex is green c, (**B**) RoG, (**C**) SASA, (**D**) H-bonds number of, (**E**) RMSF, and (**F**) mass distance center between the designed theobromine derivative and the EGFR.

The MD analyses carried out on the production run showed that, overall, the designed theobromine derivative-EGFR system was stable. The RMSD plot (Figure 8A) showed a stable average after the first 8 ns at 1.93 Å for the EGFR, blue curve, and the obtained complex, green curve. Furthermore, the RMSD of the designed theobromine derivative, red curve, showed a variation in values ranging from 0.5 Å to, approximately, 3.0 Å. The RMSD of the designed theobromine derivative can be divided into three parts. During the first 20 ns, it shows a large variation (from 0.5 to 2.72 Å) with an average of 1.41 Å, while the next 40 ns shows a small variation of approximately 1 Å (from 1.02 to 2.24 Å) with approximately the same average value of 1.41 Å. The last 40 ns show a similar variation in the values ranging from 1.05 to 3.19 Å with an average value of 1.96 Å. The radius of gyration, RoG, (Figure 8B), solvent accessible surface area, SASA, (Figure 8C), and H-bonds (Figure 8D) showed a stable protein (EGFR) fluctuation expressing an average of 19.58 Å, 15288 Å^2^, and 60 bonds, respectively. The fluctuated amino acids depicted in the root–mean–square fluctuation, RMSF, plot (Figure 8E) showed low fluctuation (less than 2 Å), with an exception of the free *N*-terminal reaching 6 Å and the loop region E841:V852. During the 100 ns, the designed theobromine derivative remained almost in the same place relating the EGFR center of mass (Figure 8F), with a 10.26 Å average.

#### 2.1.3. MM-GBSA

**Figure 9:**
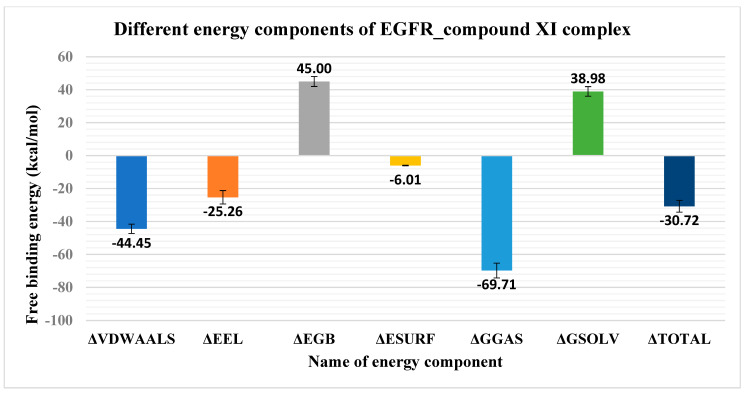
Different energetic components of MM-GBSA and their average values. Bars represent the standard deviation.

**Figure 10:**
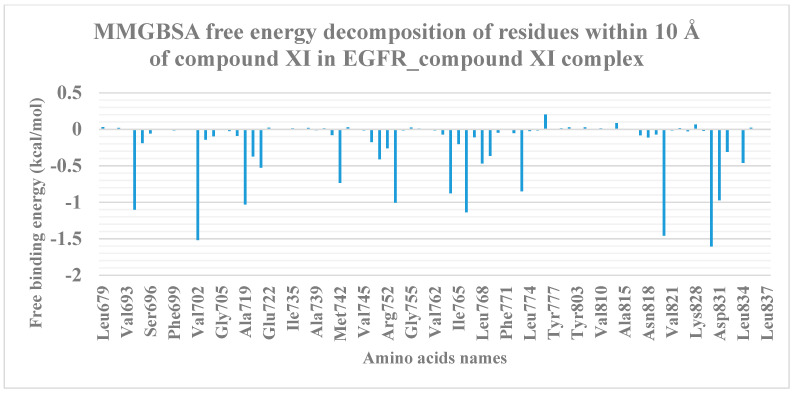
Binding-free energy decomposition of the EGFR, the designed theobromine derivative complex.

The binding exact free energy investigation through molecular mechanics with generalized born and surface area, MM-GBSA, (Figure 9) indicates the different energy components that contribute to the process of binding. The designed theobromine derivative showed a total binding energy with the average value of −30.72 kcal/mol. The most favorable calculated contribution in the energy was for the Van Der Waals interaction (average value = −44.45 kcal/mol). Coming next, the electrostatic interactions (average of −25.26 kcal/mol). Furthermore, a decomposition analysis (Figure 10) was performed to disclose which amino acid residues within 1 nm of the designed theobromine derivative have more contribution to the binding. Leu694 (−1.1), Val702 (1.51), Ala719 (−1.02), Leu753 (−1.0), Thr766 (−1.13), Leu820 (−1.45), and Thr830 (−1.6) are the key amino acid residues that have a significant contribution of a value that is less than −1 kcal/mol.

#### 2.1.4. Protein–Ligand Interaction Profiler (PLIP) Studies

**Figure 11:**
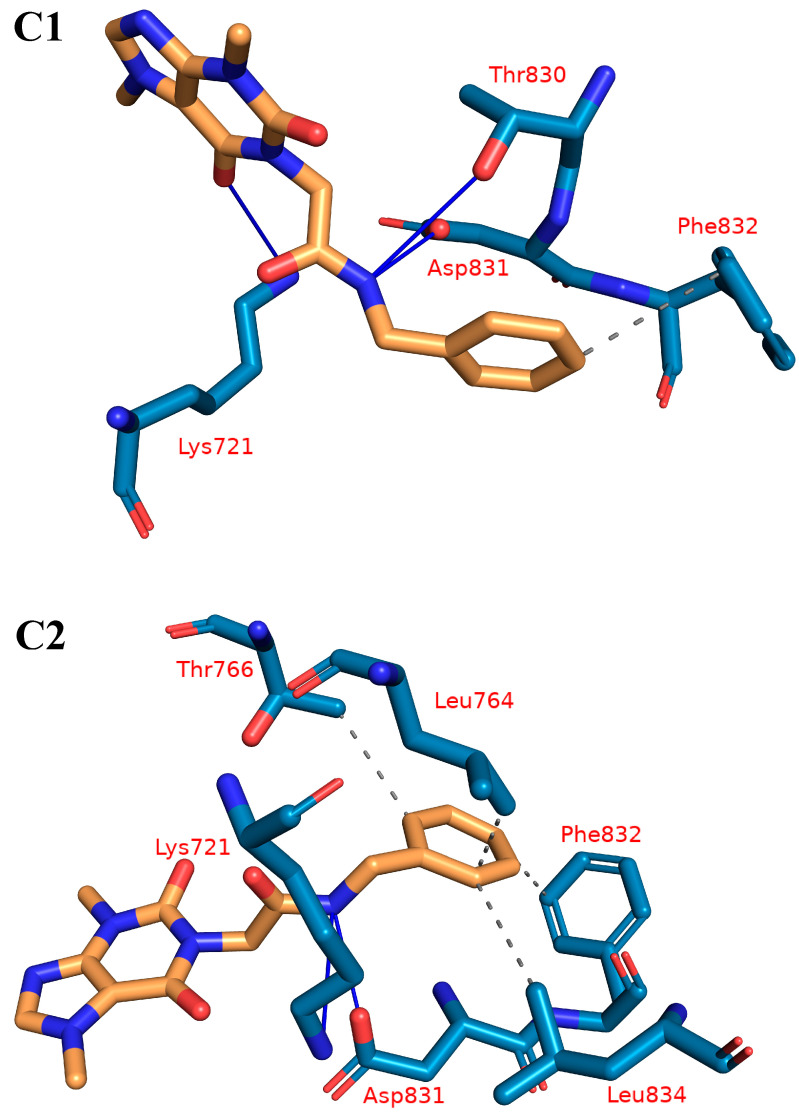

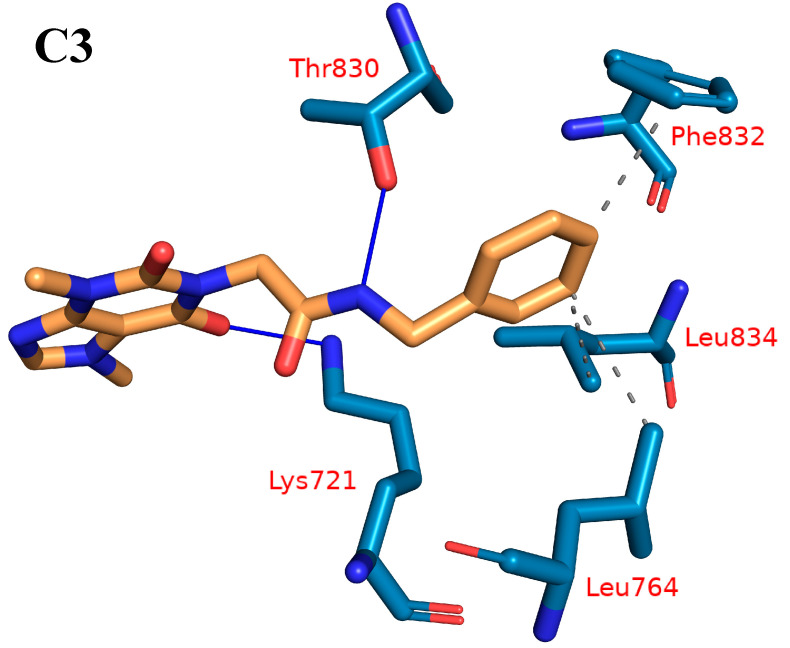
(**C1**–**C3**) Types and number of different interactions produced from PLIP for each cluster representative. H-bond: Blue solid line, H-I: dashed gray line, amino acids: blue sticks representation, and the designed theobromine derivative: orange sticks representation.

Next, the trajectories of the computed MD were clustered, providing several representative frames that represent every obtained cluster. For every cluster, the PLIP webserver was employed to identify the number as well as the types of the interactions that occur between the designed theobromine derivative and EGFR. Table 1 denotes both number as well as types of interactions that produced from the PLIP webserver. In the first cluster representative, the predominated interaction is a H-B with three bonds. On the other hand, the two remaining cluster representatives had H-Is greater than the H-Bs. Besides obtaining types and numbers of interactions, the PLIP also generated a.pse file to see the 3D conformation of the designed theobromine derivative and its interaction with EGFR (Figure 11).

#### 2.1.5. DFT

##### Geometry Optimization

**Figure 12:**
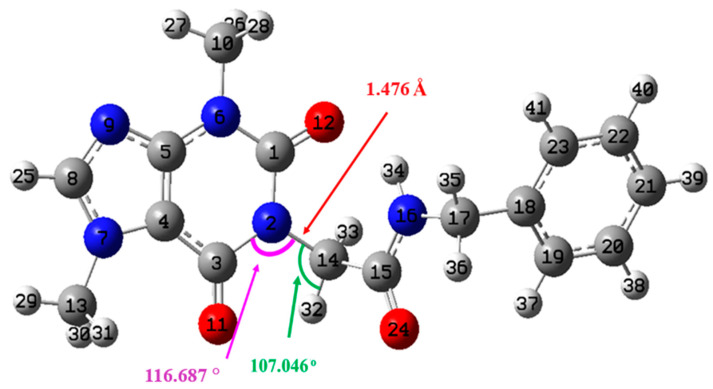
The optimized chemical structure of the compound at B3LYP/6-311G + +(d,p) level.

All calculations were performed at the B3LYP/6-311G + +(d,p) level of theory. The full optimization of the compound was run without any constraints and presented in Figure 12. The bonding between the intermediate and the reactant through the C14-N2 bond is highlighted with the two angles on either side of the bond as shown in Figure 12. The polarity of any system is determined by the dipole moment (*µ*), which measures the interaction inside the molecule. The *µ* value of the title compound is 5.8158 Debye (D), and the calculated ground total energy (TE) is −30469.8 eV (Table 2).

##### Frontier Molecular Orbital (FMO) Analysis

**Figure 13:**
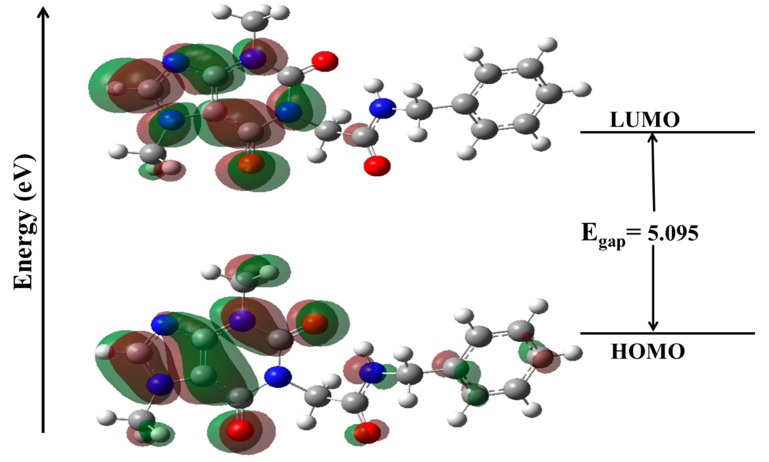
The FMO analysis; HOMO and LUMO at the ground state of the molecule at B3LYB/6-311 + +G(d,p).

From the FMO analysis, the calculated energy gap (E_gap_) between the HOMO (highest occupied molecular orbital) and LUMO (lowest unoccupied molecular orbital) for the ligand is −5.095 eV and the schematic diagram is presented in Figure 13. The E_gap_ value is relatively small and reflects a narrow frontier orbital HOMO-LUMO gap. Based on the Dm and E_gap_ values, the molecule is highly polarizable and chemically reactive [63]. The calculated energy of HOMO (E_HOMO_), LUMO (E_LUMO_), and E_gap_ was listed in Table 2.

##### Global Reactive Indices and Total Density of State (TDOS)

The quantum calculations can be used effectively to obtain the global reactivity indices, which provides details about the chemical reactivity or inhibition ability of a molecule. Global reactive indices such as global chemical hardness (*η*) and softness (*σ*) describe the inhibitory ability of the molecule to be stable or reactive. A molecule is thought to be a good inhibitor when its hardness value (*η*) is small and softness value (*σ*) is high. All electronic parameters and reactivity indices of the molecule, such as ionization potential (IP), electrophilicity (*ω*), maximal charge acceptance (∆N_max_), chemical potential (*µ*), energy change (∆E), global chemical softness (*σ*), global chemical hardness (*η*), electronegativity (*χ*), and electron affinity (EA), were estimated according to Koopmans’ theory (more information in the supporting data).

Based on the calculated values of global reactive indices (Table 2), the molecule under investigation is chemically reactive and can be a good inhibitor against the EGFR protein [64].

**Figure 14:**
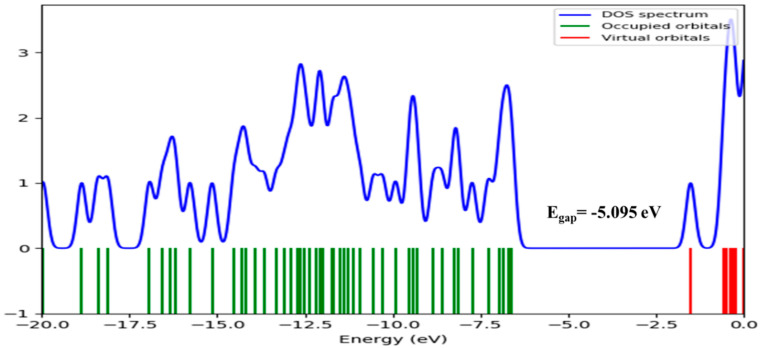
Molecular frontier orbital spectrum, total density of states (TDOS) and energies obtained at B3LYP/6-311 + +G(d,p) level of the designed compound.

At the border area, LUMO and HOMO may not provide a meaningful description of FMO due to the potential of quasi-degenerate levels. At equilibrium state, the product of the density of states of a chemical system and its probability distribution function gives the number of occupied states forunit volume. Such a number is utilized to study the physical properties of chemical systems. In this study, the TDOS analysis has been performed and the relative results confirmed that the compound under study had a significant small energy gap, and the highest intensity was reported for orbitals over LUMO orbital (Figure 14). Such results emphasized the promising inhibitor’s efficiency.

##### Surface Potential Mapping

**Figure 15:**
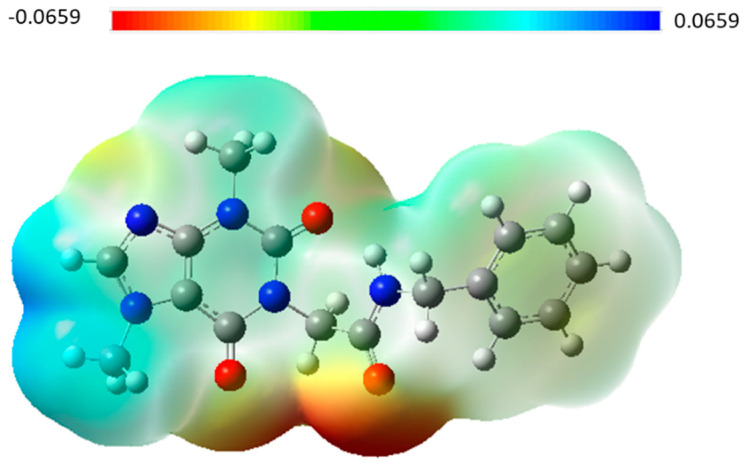
Molecular electrostatic potential map of the designed derivative at the 6-311G + +(d,p) basis set.

The potential (electron + nuclei) mapping displays the distribution, molecular shape, size, and dipole moments of the electrostatic potential of the molecule [65]. Graphically, molecular electrostatic potential is shown in Figure 15, where the color-coded red, yellow, blue, and green regions denote the electron-rich, slightly electron-rich, electron-deficient, and neutral zones, respectively. The areas around the carbonyl groups represent the most negative potential zones and the slightly electron-rich regions with yellow colors are localized over terminal phenyl and **N9** of purine moiety. The electron-deficient areas of blue color are localized on the purine substituted methyl (**C13**) and **C8H25** of purine.

#### 2.1.6. Absorption, Distribution, Metabolism, Excretion, and Toxicity (ADMET) Profiling Study

**Figure 16:**
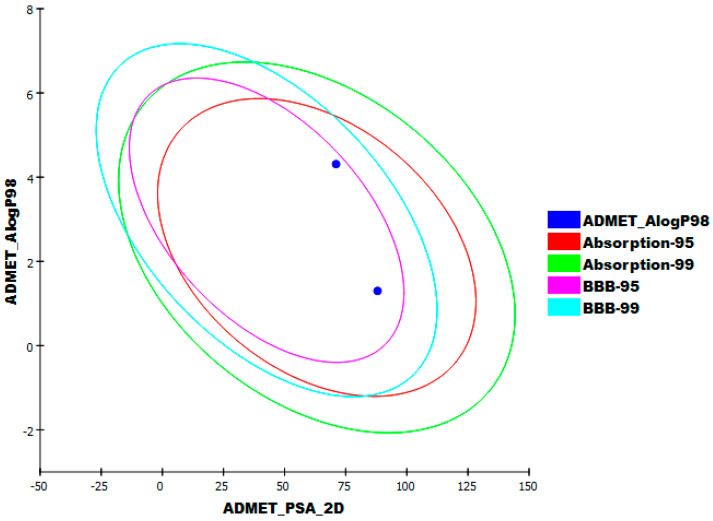
Computational prediction of ADMET parameters for the designed theobromine derivative and erlotinib.

Compounds are approved for use as drugs based on their pharmacokinetic evaluation along with their biological activity, so the pharmacokinetic evaluation of any new compound should be carried out at an early stage of its creation to help prevent the withdrawal of a drug after its approval. ADMET identifies absorption, distribution, metabolism, excretion, and toxicity, but despite the fact that in vitro studies can illustrate these properties, in silico analyses are more advantageous because of the limitations of cost, time, and effort, as well as the strict regulations regarding animal lives [66]. Computed ADMET parameters for the designed theobromine derivative against erlotinib as a reference molecule were determined using Discovery studio 4.0. According to ADMET results, the designed theobromine derivative and erlotinib exhibited similar good levels of solubility and intestinal absorption. Additionally, affinity to pass the blood–brain barrier, BBB, and to inhibit the cytochrome P450 (CYP2D6) was predicted as low and non-inhibitors, respectively. The two compounds (the designed theobromine derivative and erlotinib) showed a difference in the ability to bind with plasma protein as it was predicted to be less and more than 90%, respectively (Figure 16).

#### 2.1.7. In Silico Toxicity Studies

In silico approaches to toxicity prediction have played an indispensable role in drug development, as it avoids ethical regulation, resource availability, and time-wasting in traditional in vitro and in vivo studies [67]. This approach has played an indispensable role in the discovery of new drugs and treatments [68]. In silico toxicity prediction uses structure–activity relationships (SARs) to predict toxicities, and in detail, the computer compares the chemical structural properties of the molecules to thousands of compounds that have been reported to be safe or toxic [69]. A toxicity model built in Discovery studio software was used to estimate eight toxicity parameters. Table 3 shows that the designed theobromine derivative demonstrated high levels of safety in the computed models.

### 2.2. Chemistry

**Scheme 1:**
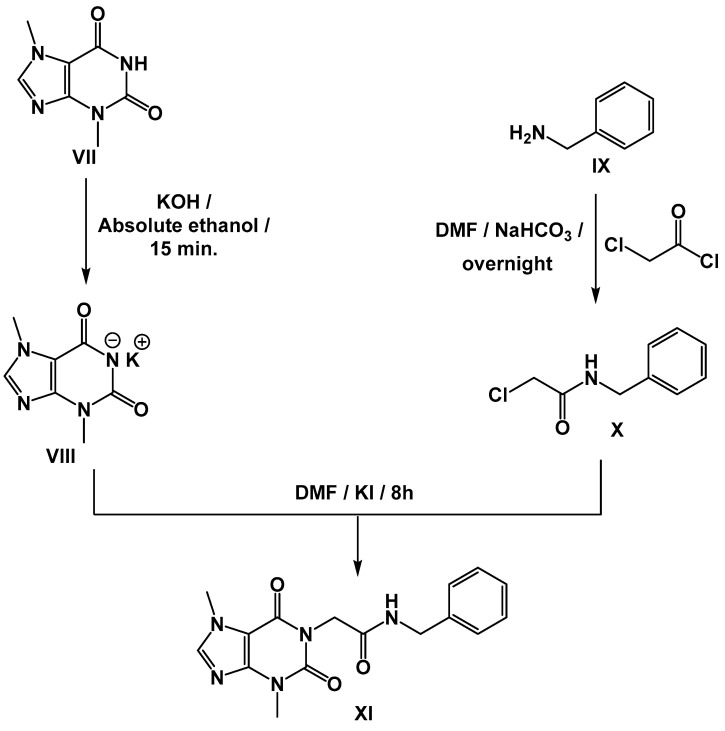
Synthesis of the target theobromine derivative **XI**.

Because the designed theobromine derivative demonstrated high affinity to bind with EGFR enzyme through different computational studies and it showed a good range of drug-likeness, it was semi-synthesized by the modification of theobromine. In the present work, potassium salt formation was achieved on the imidic nitrogen of theobromine **VII** [70] by treating it with alcoholic KOH while continuously stirring. The formed salt **VIII** was then reacted with *N*-benzyl-2-chloroacetamide **X**, which was formed via the reaction of phenylmethanamine **IX** with chloroacetylchloride in DMF, to produce the corresponding target product **XI** (Scheme 1).

The IR spectrum of compound **XI** was characterized by the appearance of carbonyl absorption bands at 1711 and 1656 cm^−1^. Moreover, the ^1^H NMR revealed the presence of amidic proton at 8.61 ppm. Moreover, a characteristic singlet signal appeared at 4.52 ppm corresponding to the CH_2_ group. The ^13^C NMR spectrum, which displayed distinct peaks at approximately 43.44, 42.52, 33.67, and 29.91 ppm corresponding to the two CH_3_ and two CH_2_ groups, respectively, further confirmed the validity of the proposed structure.

**Figure 17:**
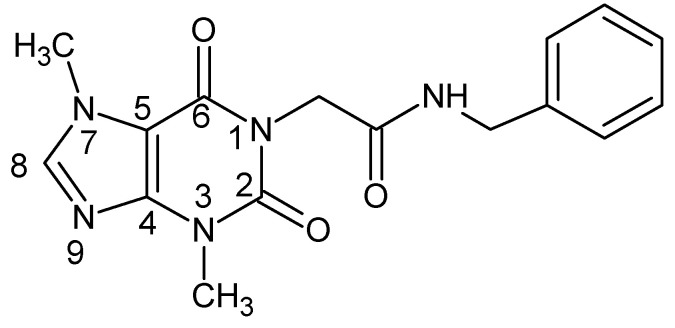
Chemical structure of compound **XI**.

The chemical structure of compound **XI** (Figure 17) was confirmed through spectroscopic analysis. In detail, the NH proton resonated clearly at δ 8.61 as a triplet signal (*J* = 5.9 Hz) with the integration of one due to the presence of neighboring CH_2_. Two CH_2_ groups were detected at δ 4.30 (d, *J* = 5.9 Hz, 2H), which is adjacent to the NH, and at 4.52 (s, 2H) which is adjacent to the carbonyl. The proton of C-8, the only proton of the theobromine rings, resonated at δ 8.07 (s, 1H). The two methyls of the theobromine moiety (3, 7) resonated as two singlet peaks at δ 3.44, and 3.90, respectively. Furthermore, a pattern of monosubstituted benzene ring was discovered at δ 7.37–7.31 (2H) and 7.26 (3H). Similarly, the ^13^C NMR showed three carbonyls at δ 167.50, for the acetamide moiety, in addition to the two up-field carbonyls of the theobromine moiety because of the high anisotropic effects at δ 154.73 and 151.39. Additionally, C-4, C-5, and C-8 of the theobromine moiety resonated at 148.97, 107.18, and 143.53, respectively. The monosubstituted benzene ring shoed its characteristic signals at 139.72, 128.75 (2C), 127.57 (2C), and 127.25. Two methylene and two methyl groups were detected at 43.44, 42.52, 33.67, and 29.91, respectively.

### 2.3. Biological Evaluation

#### 2.3.1. EGFR Inhibition

**Figure 18:**
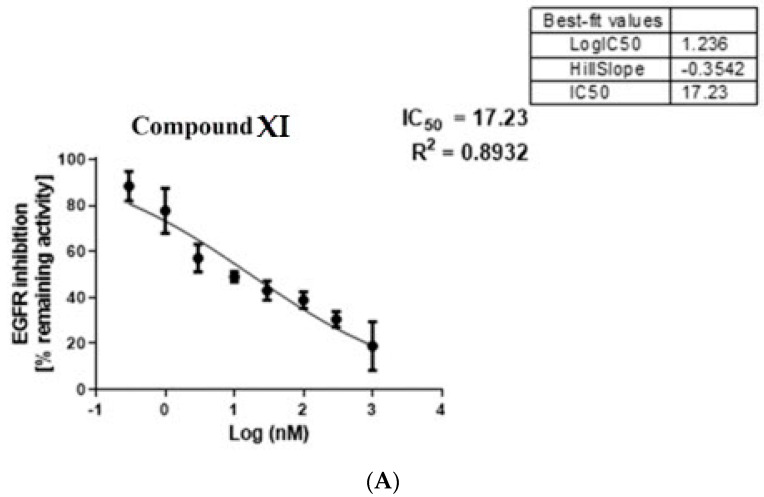

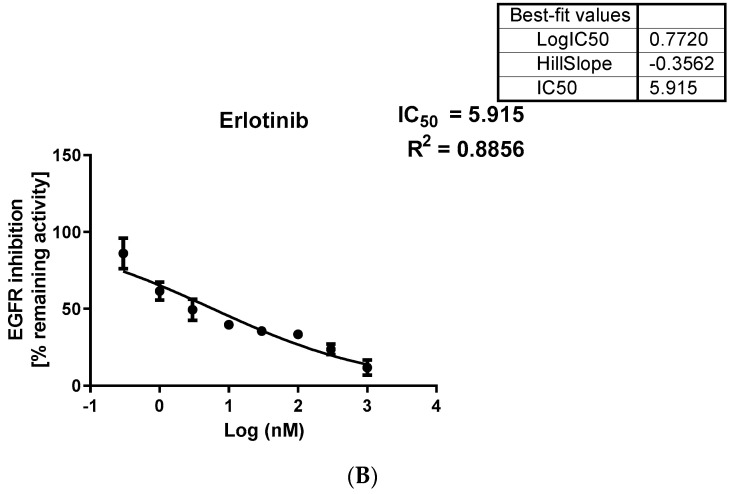
In vitro EGFR inhibition of compound **XI** (**A**) and erlotinib (**B**).

To examine the design and the computational results that indicated the strong binding affinity of compound **XI** to the EGFR enzyme, compound **XI** was tested in vitro against the EGFR enzyme in comparison with erlotinib. As shown in Figure 18, compound **XI** strongly inhibited the EGFR enzyme with 17.2 nM that was bordering erlotinib’s value. The obtained results were consistent with the acquired in silico results and confirmed the strong suppressing potential of compound **XI**.

#### 2.3.2. Cytotoxicity and Safety

An in vitro cytotoxicity assay to evaluate compound **XI**’s EGFR inhibition against cancer was conducted using the A549 and HCT-116 malignant cell lines against erlotinib as a reference. Compound **XI** demonstrated IC_50_ values of 21.99 and 22.02 µM, respectively (Table 4). The anticancer potentials of compound **XI** were very close to those of erlotinib (6.73 and 16.35 µM, respectively).

**Figure 19:**
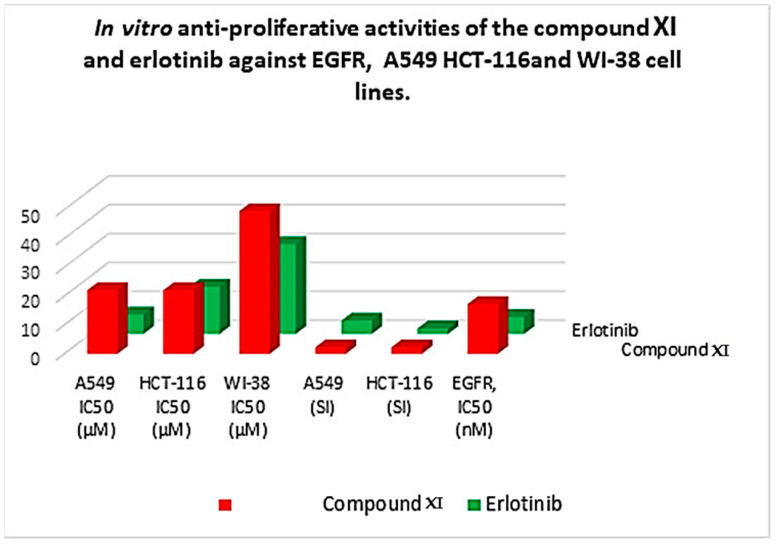
In vitro anti-proliferative activities of the compound **XI** and erlotinib against EGFR, cancer and normal cell lines.

The cytotoxic potential of compound **XI** against the W138 normal human cell line was evaluated to confirm its in silico safety results and determine its selectivity against cancer cell lines. Compound **XI** displayed excellent safety results, expressing a high IC_50_ value of 49.44 µM (safer than erlotinib) and very high selectivity indexes (SI) against both examined cell lines of 2.2 (Figure 19).

#### 2.3.3. Cell Cycle Analysis and Apoptosis Assay

**Figure 20:**
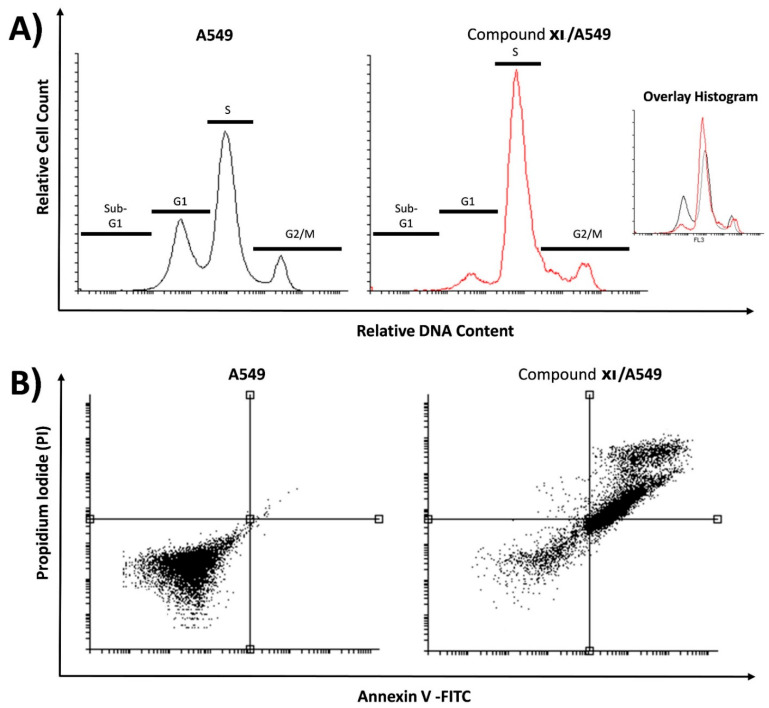
(**A**) Cell cycle and (**B**) apoptotic analysis of compound **XI** and control untreated **A549** cell line.

Initially, flow cytometric analysis of cell cycle phases was carried out [71,72]. Compound **XI** was treated with A549 cells for 72 h at a concentration of 21.99 µM. Then, the different stages of the cell cycle were examined. Compound **XI** decreased the growth of cells in the Sub-G1 and G1 phases from 0.87% to 0.80% and from 43.47% to 16%, respectively. Conversely, the f **A549** population percentage was significantly increased from 12.10 for control cells to 19.84 for **XI**-treated cells in the G2/M phase (Table 5 and Figure 20). To confirm the apoptotic effects of **XI**, A549 cells were stained with Annexin V and PI double stains after treatment of with 21.99 µM of **XI** for 72 h [73,74]. Comparing the control, compound **XI** induced a higher number of apoptotic cells. Compound **XI** caused a significant increase in the apoptotic cells percentage in both early apoptosis (from 0.05% to 24.02%) and late apoptosis (from 0.49% to 41.70%). A noticeable change in the total apoptosis percentage by 65.72 was observed, compared to 0.54% in the control cells (Figure 20 and Table 6). In conclusion, compound **XI** primarily arrested the cancer cell cycle at the G2/M stage caused cytotoxic activities that may be due to programmed apoptosis. (See Supplementary Materials).

## 3. Experimental

### 3.1. In Silico Studies

#### 3.1.1. Docking Studies

The molecular docking was carried out by MOE2014 software [75]. Supplementary data give a detailed explanation.

#### 3.1.2. MD Simulations

CHARMM-GUI web server and GROMACS 2021 were utilized as an MD engine [76,77]. Supplementary data give a detailed explanation.

#### 3.1.3. MM-GBSA

The Gmx_MMPBSA package was utilized [78]. Supplementary data give a detailed explanation.

#### 3.1.4. DFT

Gaussian 09 and GaussSum3.0 programs [79] were utilized [80]. Supplementary data give a detailed explanation.

#### 3.1.5. ADMET Studies

ADMET profile was carried out by Discovery Studio 4.0 [81]. Supplementary data give a detailed explanation.

#### 3.1.6. Toxicity Studies

The toxicity profile was carried out by Discovery Studio 4.0. Supplementary data give a detailed explanation.

### 3.2. Chemistry

#### Synthesis of Compound **XI**

**Scheme 2:**
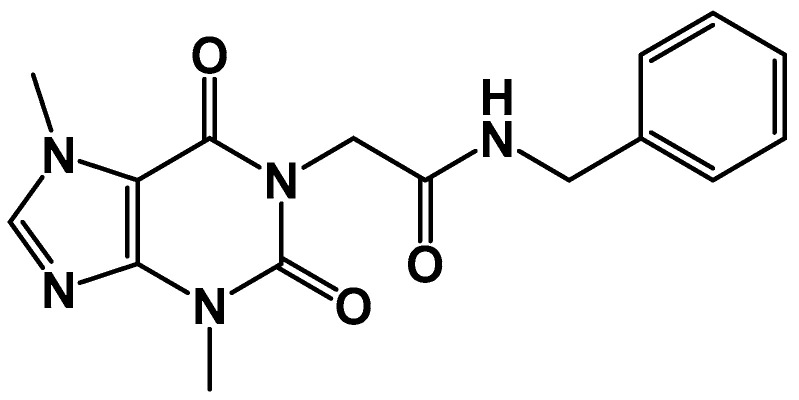
*N*-Benzyl-2-(3,7-dimethyl-2,6-dioxo-2,3,6,7-tetrahydro-1H-purin-1-yl)acetamide (**XI**).

To a solution of the potassium salt of theobromine **VIII** (0.001 mol, 0.25 g) in dry DMF (10 mL), *N*-benzyl-2-chloroacetamide **X** (0.001 mol, 0.21 g) was added and the mixture was heated for 5 h. The mixture was then cooled and the produced precipitate was filtered, washed with water, and crystallized from ethanol to attain the final target compound **XI** (Scheme 2).

Off-white crystal (yield, 84%); m. p. = 257–259 °C; IR (KBr) *ν* cm^−1^: 3282, 3108 (NH), 2929 (CH aliphatic), 1711, 1656 (C = O); ^1^H NMR (400 MHz, DMSO-*d*_6_) δ 8.61 (t, *J* = 5.9 Hz, 1H), 8.07 (s, 1H), 7.37–7.31 (m, 2H), 7.26 (d, *J* = 7.5 Hz, 3H), 4.52 (s, 2H), 4.30 (d, *J* = 5.9 Hz, 2H), 3.90 (s, 3H), 3.44 (s, 3H); ^13^C NMR (101 MHz, DMSO-d_6_) δ 167.50, 154.73, 151.39, 148.97, 143.53, 139.72, 128.75 (2C), 127.57 (2C), 127.25, 107.18, 43.44, 42.52, 33.67, 29.91. For C_16_H_17_N_5_O_3_ (327.34).

### 3.3. Biological Studies

#### 3.3.1. In Vitro Egfr Inhibition

This was performed using the Human EGFR ELISA kit. The supplementary data provide a thorough explanation.

#### 3.3.2. In Vitro Antiproliferative Activity

MTT procedure was utilized. The supplementary data provide a detailed explanation.

#### 3.3.3. Safety Assay

The normal cell lines, W138, were utilized. The supplementary data provide a detailed explanation.

#### 3.3.4. Cell Cycle Analysis and Apoptosis

The effect of compound **XI** on cell cycle distribution and apoptosis was performed using flowcytometry analysis technique. The supplementary data provide a detailed explanation.

## 4. Conclusions

Based on the essential structural properties of EGFR inhibitors, a new theobromine derivative was designed as an inhibitor. The potentiality of the designed theobromine derivative against EGFR was demonstrated by molecular docking against both wild and mutant types. The binding was confirmed by six MD (over 100 ns), two MM-GBSA, PLP, and four DFT experiments. In addition, an ADMET analysis confirmed the general likeness and safety. In vitro results were consistent with the in silico results, displaying EGFR inhibition with an IC_50_ value of 17.23 nM and cytotoxic properties against A549 and HCT-116 cell lines with IC_50_ values of 21.99 and 22.02 µM, respectively. Compound **XI** was much safer against the healthy W138 cell line (IC_50_ = 49.44 µM) than erlotinib (IC_50_ = 31.17 µM), showing a selectivity index of 2.2. Compound **XI** arrested the A549 cell cycle at the G2/M stage and induced apoptosis. According to these results, compound **XI** could be a new lead compound in the discovery of anti-EGFR candidates through further modifications and more examinations.

The results show that compound **XI** (the theobromine derivative) is a lead compound that could be employed for further modifications or in vivo and preclinical studies.

## Figures and Tables

**Table 1:** Interactions (number and types) detected from the PLIP webserver. Bold amino acids are common in all clusters.

Cluster Number	H-Is	Amino Acids in EGFR	H-Bs	Amino Acids in EGFR
C1	1	F832	3	Lys721-Thr830-Asp831
C2	4	Leu764-Thr766-Phe832-Leu834	2	Lys721-Asp831
C3	3	Leu764-Phe832-Leu834	2	Lys721-Thr830

**Table 2:** The calculated global reactivity indices and energetic parameters for the selected compound.

IP	EA	*μ* (eV)	*χ* (eV)	*η* (eV)	*σ* (eV)	*ω* (eV)	*µ* (D)	TE (eV)	∆Nmax	∆E (eV)
−1.512	−6.607	−4.059	4.059	−2.548	−0.393	−20.987	5.816	−30469.8	−1.593	20.987

**Table 3:** In silico toxicity studies of the designed theobromine derivative and erlotinib.

Comp.	FDA Rodent Carcinogenicity (Mouse-Male)	Carcinogenic Potency TD_50_ (Mouse) ^a^	Ames Mutagenicity	Rat Maximum Tolerated Dose (Feed) ^b^	Rat Oral LD_50_^b^	Rat Chronic LOAEL ^b^	Skin Irritancy	Ocular Irritancy
The designed compound	Non-Carcinogen	70.942	Non-Mutagen	0.024	1.575	0.039	Non-Irritant	Mild
Erlotinib		39.771		0.083	0.662	0.036		

^a^ Unit: mg/kg/day. ^b^ Unit: g/kg.

**Table 4:** In vitro anti-proliferative activities of the compound **XI** and erlotinib against EGFR, A549 HCT-116, and WI-38 cell lines.

Comp.	In Vitro Cytotoxicity IC_50_ (µM) ^a^			A549 (SI)	HCT-116 (SI)	EGFR, IC_50_ (nM)
	A549	HCT-116	WI-38			
Compound **XI**	21.99	22.02	49.44	2.2	2.2	17.23
Erlotinib	6.73	16.35	31.17	4.6	1.9	5.91

^a^ Data are presented as the mean of the IC_50_ values from three different experiments.

**Table 5:** Cell cycle analysis of compound **XI** and control untreated **A549** cell line.

Sample	Cell Cycle Distribution (%) ^a^			
	% Sub-G1	% G1	% S	% G2/M
A549	0.87 ± 0.35	43.47 ± 3.99	43.56 ± 2.31	12.10 ± 2.04
Compound XI/A549	0.80 ± 0.17	16.01 ± 5.00	63.35 ± 2.50 *	19.84 ± 2.69

^a^ All values are calculated as the mean of triplicates. * *p* < 0.1.

**Table 6:** Effect of compound **XI** on A549 cells’ stages of death.

Sample	Viable ^a^ (Left Bottom)	Apoptosis ^a^		Necrosis ^a^ (Left Top)
		Early (Right Bottom)	Late (Right Top)	
A549	99.44 ± 0.46	0.05 ± 0.02	0.49 ± 0.46	0.02 ± 0.01
Compound XI/A549	32.55 ± 2.98	24.02 ± 1.52 **	41.70 ± 1.76 **	1.73 ± 0.30

^a^ All values are calculated as the mean of at least three different experiments. ** *p* < 0.01.

## Data Availability

Data are available upon request.

## Supplementary Materials

The following are available online at https://www.mdpi.com/article/10.3390/molecules27185859/s1. Detailed toxicity report, the detailed methods, spectral data, and toxicity report.

## Sample Availability

Compound **XI** is available from the authors.

## References

[1] Abd El-Mageed M.M., Eissa A.A., Farag A.E.-S., Osman E.E.A. (2021). Design and synthesis of novel furan, furo [2, 3-d] pyrimidine and furo [3, 2-e][1,2,4] triazolo [1, 5-c] pyrimidine derivatives as potential VEGFR-2 inhibitors. Bioorganic Chem..

[2] Chaudhari P., Bari S., Surana S., Shirkhedkar A., Wakode S., Shelar S., Racharla S., Ugale V., Ghodke M. (2021). Logical synthetic strategies and structure-activity relationship of indolin-2-one hybrids as small molecule anticancer agents: An Overview. J. Mol. Struct..

[3] El-Dash Y., Elzayat E., Abdou A.M., Hassan R.A. (2021). Novel thienopyrimidine-aminothiazole hybrids: Design, synthesis, antimicrobial screening, anticancer activity, effects on cell cycle profile, caspase-3 mediated apoptosis and VEGFR-2 inhibition. Bioorganic Chem..

[4] Nicholson R.I., Gee J.M.W., Harper M.E. (2001). EGFR and cancer prognosis. Eur. J. Cancer.

[5] Spano J.-P., Lagorce C., Atlan D., Milano G., Domont J., Benamouzig R., Attar A., Benichou J., Martin A., Morere J.-F. (2005). Impact of EGFR expression on colorectal cancer patient prognosis and survival. Ann. Oncol..

[6] Normanno N., De Luca A., Bianco C., Strizzi L., Mancino M., Maiello M.R., Carotenuto A., De Feo G., Caponigro F., Salomon D.S. (2006). Epidermal growth factor receptor (EGFR) signaling in cancer. Gene.

[7] Ayati A., Moghimi S., Salarinejad S., Safavi M., Pouramiri B., Foroumadi A. (2020). A review on progression of epidermal growth factor receptor (EGFR) inhibitors as an efficient approach in cancer targeted therapy. Bioorganic Chem..

[8] Metwaly A.M., Ghoneim M.M., Eissa I.H., Elsehemy I.A., Mostafa A.E., Hegazy M.M., Afifi W.M., Dou D. (2021). Traditional ancient Egyptian medicine: A review. Saudi J. Biol. Sci..

[9] Han X., Yang Y., Metwaly A.M., Xue Y., Shi Y., Dou D. (2019). The Chinese herbal formulae (Yitangkang) exerts an antidiabetic effect through the regulation of substance metabolism and energy metabolism in type 2 diabetic rats. J. Ethnopharmacol..

[10] Newman D.J., Cragg G.M. (2016). Natural products as sources of new drugs from 1981 to 2014. J. Nat. Prod..

[11] Barcz E., Sommer E., Janik P., Marianowski L., Skopinska-Rozewska E. (2000). Adenosine receptor antagonism causes inhibition of angiogenic activity of human ovarian cancer cells. Oncol. Rep..

[12] Kakuyamanee Iwazaki A., Sadzuka Y. (2001). Effect of methylxanthine derivatives on doxorubicin transport and antitumor activity. Curr. Drug Metab..

[13] Sultani H.N., Ghazal R.A., Hayallah A.M., Abdulrahman L.K., Abu-Hammour K., AbuHammad S., Taha M.O., Zihlif M.A. (2017). Inhibitory effects of new mercapto xanthine derivatives in human mcf7 and k562 cancer cell lines. J. Heterocycl. Chem..

[14] Woskresensky A. (1842). Ueber das Theobromin. Justus Liebigs Ann. Der Chem..

[15] Smit H.J. (2011). Theobromine and the pharmacology of cocoa. Methylxanthines.

[16] Fischer E. (1907). Synthese des Theobromins. Untersuchungen in der Puringruppe.

[17] Carla Cadoná F., Kolinski Machado A., Farina Azzolin V., Barbisan F., Bortoluzzi Dornelles E., Glanzner W., Bayard Gonçalves P.D., Elias Assmann C., Esteves Ribeiro E., Beatrice Mânica da Cruz I. (2016). Guaraná a caffeine-rich food increases oxaliplatin sensitivity of colorectal HT-29 cells by apoptosis pathway modulation. Anti-Cancer Agents Med. Chem. (Former. Curr. Med. Chem.-Anti-Cancer Agents).

[18] Shojaei-Zarghani S., Khosroushahi A.Y., Rafraf M. (2021). Oncopreventive effects of theanine and theobromine on dimethylhydrazine-induced colon cancer model. Biomed. Pharmacother..

[19] Lee H.J., Lee K.W., Kang K.S., Kim D.Y., Park H.H., Lee M.J., Kim H.S., Kwon I.B. (2001). Theobromine with an Anti-Carcinogenic Activity. European Patent.

[20] Sugimoto N., Miwa S., Hitomi Y., Nakamura H., Tsuchiya H., Yachie A. (2014). Theobromine, the primary methylxanthine found in Theobroma cacao, prevents malignant glioblastoma proliferation by negatively regulating phosphodiesterase-4, extracellular signal-regulated kinase, Akt/mammalian target of rapamycin kinase, and nuclear factor-kappa B. Nutr. Cancer.

[21] Gil M., Skopińska-Rózewska E., Radomska D., Demkow U., Skurzak H., Rochowska M., Beuth J., Roszkowski K. (1993). Effect of purinergic receptor antagonists suramin and theobromine on tumor-induced angiogenesis in BALB/c mice. Folia Biol..

[22] Barcz E., Sommer E., Sokolnicka I., Gawrychowski K., Roszkowska-Purska K., Janik P., Skopinska-Rózewska E. (1998). The influence of theobromine on angiogenic activity and proangiogenic cytokines production of human ovarian cancer cells. Oncol. Rep..

[23] da Rosa R., Schenkel E.P., Bernardes L.S.C. (2020). Semisynthetic and newly designed derivatives based on natural chemical scaffolds: Moving beyond natural products to fight Trypanosoma cruzi. Phytochem. Rev..

[24] Goh G.B., Hodas N.O., Vishnu A. (2017). Deep learning for computational chemistry. J. Comput. Chem..

[25] Eissa I.H., Alesawy M.S., Saleh A.M., Elkaeed E.B., Alsfouk B.A., El-Attar A.-A.M., Metwaly A.M. (2022). Ligand and Structure-Based In Silico Determination of the Most Promising SARS-CoV-2 nsp16-nsp10 2′-o-Methyltransferase Complex Inhibitors among 3009 FDA Approved Drugs. Molecules.

[26] Elkady H., Elwan A., El-Mahdy H.A., Doghish A.S., Ismail A., Taghour M.S., Elkaeed E.B., Eissa I.H., Dahab M.A., Mahdy H.A. (2022). New benzoxazole derivatives as potential VEGFR-2 inhibitors and apoptosis inducers: Design, synthesis, anti-proliferative evaluation, flowcytometric analysis, and in silico studies. J. Enzym. Inhib. Med. Chem..

[27] Elkaeed E.B., Yousef R.G., Elkady H., Gobaara I.M.M., Alsfouk A.A., Husein D.Z., Ibrahim I.M., Metwaly A.M., Eissa I.H. (2022). The Assessment of Anticancer and VEGFR-2 Inhibitory Activities of a New 1H-Indole Derivative: In Silico and In Vitro Approaches. Processes.

[28] Mohammed S.O., El Ashry E.S.H., Khalid A., Amer M.R., Metwaly A.M., Eissa I.H., Elkaeed E.B., Elshobaky A., Hafez E.E. (2022). Expression, Purification, and Comparative Inhibition of Helicobacter pylori Urease by Regio-Selectively Alkylated Benzimidazole 2-Thione Derivatives. Molecules.

[29] Alanazi M.M., Elkady H., Alsaif N.A., Obaidullah A.J., Alkahtani H.M., Alanazi M.M., Alharbi M.A., Eissa I.H., Dahab M.A. (2021). New quinoxaline-based VEGFR-2 inhibitors: Design, synthesis, and antiproliferative evaluation with in silico docking, ADMET, toxicity, and DFT studies. RSC Adv..

[30] Suleimen Y.M., Jose R.A., Suleimen R.N., Arenz C., Ishmuratova M.Y., Toppet S., Dehaen W., Alsfouk B.A., Elkaeed E.B., Eissa I.H. (2022). Jusanin, a New Flavonoid from Artemisia commutata with an In Silico Inhibitory Potential against the SARS-CoV-2 Main Protease. Molecules.

[31] Alanazi M.M., Elkady H., Alsaif N.A., Obaidullah A.J., Alanazi W.A., Al-Hossaini A.M., Alharbi M.A., Eissa I.H., Dahab M.A. (2021). Discovery of new quinoxaline-based derivatives as anticancer agents and potent VEGFR-2 inhibitors: Design, synthesis, and in silico study. J. Mol. Struct..

[32] Suleimen Y.M., Jose R.A., Suleimen R.N., Ishmuratova M.Y., Toppet S., Dehaen W., Alsfouk A.A., Elkaeed E.B., Eissa I.H., Metwaly A.M. (2022). Isolation and In Silico SARS-CoV-2 Main Protease Inhibition Potential of Jusan Coumarin, a New Dicoumarin from Artemisia glauca. Molecules.

[33] Eissa I.H., Khalifa M.M., Elkaeed E.B., Hafez E.E., Alsfouk A.A., Metwaly A.M. (2021). In Silico Exploration of Potential Natural Inhibitors against SARS-CoV-2 nsp10. Molecules.

[34] Elkaeed E.B., Elkady H., Belal A., Alsfouk B.A., Ibrahim T.H., Abdelmoaty M., Arafa R.K., Metwaly A.M., Eissa I.H. (2022). Multi-Phase In Silico Discovery of Potential SARS-CoV-2 RNA-Dependent RNA Polymerase Inhibitors among 3009 Clinical and FDA-Approved Related Drugs. Processes.

[35] Suleimen Y.M., Jose R.A., Suleimen R.N., Arenz C., Ishmuratova M., Toppet S., Dehaen W., Alsfouk A.A., Elkaeed E.B., Eissa I.H. (2022). Isolation and In Silico Anti-SARS-CoV-2 Papain-Like Protease Potentialities of Two Rare 2-Phenoxychromone Derivatives from Artemisia spp.. Molecules.

[36] Alesawy M.S., Elkaeed E.B., Alsfouk A.A., Metwaly A.M., Eissa I.H. (2021). In silico screening of semi-synthesized compounds as potential inhibitors for SARS-CoV-2 papain-like protease: Pharmacophoric features, molecular docking, ADMET, toxicity and DFT studies. Molecules.

[37] Bonomi P. (2003). Erlotinib: A new therapeutic approach for non-small cell lung cancer. Expert Opin. Investig. Drugs.

[38] Pao W., Miller V.A., Politi K.A., Riely G.J., Somwar R., Zakowski M.F., Kris M.G., Varmus H. (2005). Acquired resistance of lung adenocarcinomas to gefitinib or erlotinib is associated with a second mutation in the EGFR kinase domain. PLoS Med..

[39] Muhsin M., Graham J., Kirkpatrick P. (2003). Fresh from the pipeline: Gefitinib. Nat. Rev. Cancer.

[40] Singh H., Walker A.J., Amiri-Kordestani L., Cheng J., Tang S., Balcazar P., Barnett-Ringgold K., Palmby T.R., Cao X., Zheng N. (2018). US Food and Drug Administration Approval: Neratinib for the Extended Adjuvant Treatment of Early-Stage HER2-Positive Breast CancerFDA Approval Summary: Neratinib. Clin. Cancer Res..

[41] Sequist L.V., Besse B., Lynch T.J., Miller V.A., Wong K.K., Gitlitz B., Eaton K., Zacharchuk C., Freyman A., Powell C. (2010). Neratinib, an irreversible pan-ErbB receptor tyrosine kinase inhibitor: Results of a phase II trial in patients with advanced non–small-cell lung cancer. J. Clin. Oncol..

[42] Kim Y., Ko J., Cui Z., Abolhoda A., Ahn J.S., Ou S.-H., Ahn M.-J., Park K. (2012). The EGFR T790M mutation in acquired resistance to an irreversible second-generation EGFR inhibitor. Mol. Cancer Ther..

[43] Carroll J. Following Lethal Tox Report, Boehringer Scraps Plans for High-Speed Development, Kills $730M Hanmi Deal. https://endpts.com/following-lethal-tox-report-boehringer-scraps-plans-for-high-speed-development-kills-730m-hanmi-deal/.

[44] Traxler P., Bold G., Frei J., Lang M., Lydon N., Mett H., Buchdunger E., Meyer T., Mueller M., Furet P. (1997). Use of a pharmacophore model for the design of EGF-R tyrosine kinase inhibitors: 4-(phenylamino) pyrazolo [3, 4-d] pyrimidines. J. Med. Chem..

[45] Ducray R., Ballard P., Barlaam B.C., Hickinson M.D., Kettle J.G., Ogilvie D.J., Trigwell C.B. (2008). Novel 3-alkoxy-1H-pyrazolo [3, 4-d] pyrimidines as EGFR and erbB2 receptor tyrosine kinase inhibitors. Bioorganic Med. Chem. Lett..

[46] Gaber A.A., Bayoumi A.H., El-Morsy A.M., Sherbiny F.F., Mehany A.B., Eissa I.H. (2018). Design, synthesis and anticancer evaluation of 1H-pyrazolo [3, 4-d] pyrimidine derivatives as potent EGFRWT and EGFRT790M inhibitors and apoptosis inducers. Bioorganic Chem..

[47] Gandin V., Ferrarese A., Dalla Via M., Marzano C., Chilin A., Marzaro G. (2015). Targeting kinases with anilinopyrimidines: Discovery of N-phenyl-N’-[4-(pyrimidin-4-ylamino) phenyl] urea derivatives as selective inhibitors of class III receptor tyrosine kinase subfamily. Sci. Rep..

[48] Traxler P., Furet P. (1999). Strategies toward the design of novel and selective protein tyrosine kinase inhibitors. Pharmacol. Ther..

[49] Zhang J., Yang P.L., Gray N.S. (2009). Targeting cancer with small molecule kinase inhibitors. Nat. Rev. Cancer.

[50] Eldehna W.M., El Hassab M.A., Elsayed Z.M., Al-Warhi T., Elkady H., Abo-Ashour M.F., Abourehab M.A.S., Eissa I.H., Abdel-Aziz H.A. (2022). Design, synthesis, in vitro biological assessment and molecular modeling insights for novel 3-(naphthalen-1-yl)-4,5-dihydropyrazoles as anticancer agents with potential EGFR inhibitory activity. Sci. Rep..

[51] Mowafy S., Galanis A., Doctor Z.M., Paranal R.M., Lasheen D.S., Farag N.A., Jänne P.A., Abouzid K.A. (2016). Toward discovery of mutant EGFR inhibitors; Design, synthesis and in vitro biological evaluation of potent 4-arylamino-6-ureido and thioureido-quinazoline derivatives. Biorg. Med. Chem..

[52] Zhao Z., Wu H., Wang L., Liu Y., Knapp S., Liu Q., Gray N.S. (2014). Exploration of type II binding mode: A privileged approach for kinase inhibitor focused drug discovery?. ACS Chem. Biol..

[53] Furet P., Caravatti G., Lydon N., Priestle J.P., Sowadski J.M., Trinks U., Traxler P. (1995). Modelling study of protein kinase inhibitors: Binding mode of staurosporine and origin of the selectivity of CGP 52411. J. Comput.-Aided Mol. Des..

[54] Liu Y., Gray N.S. (2006). Rational design of inhibitors that bind to inactive kinase conformations. Nat. Chem. Biol..

[55] Nasser A.A., Eissa I.H., Oun M.R., El-Zahabi M.A., Taghour M.S., Belal A., Saleh A.M., Mehany A.B., Luesch H., Mostafa A.E. (2020). Discovery of new pyrimidine-5-carbonitrile derivatives as anticancer agents targeting EGFR WT and EGFR T790M. Org. Biomol. Chem..

[56] Elzahabi H.S., Nossier E.S., Alasfoury R.A., El-Manawaty M., Sayed S.M., Elkaeed E.B., Metwaly A.M., Hagras M., Eissa I.H. (2022). Design, synthesis, and anti-cancer evaluation of new pyrido [2, 3-d] pyrimidin-4 (3H)-one derivatives as potential EGFRWT and EGFRT790M inhibitors and apoptosis inducers. J. Enzym. Inhib. Med. Chem..

[57] Elmetwally S.A., Saied K.F., Eissa I.H., Elkaeed E.B. (2019). Design, synthesis and anticancer evaluation of thieno [2, 3-d] pyrimidine derivatives as dual EGFR/HER2 inhibitors and apoptosis inducers. Bioorganic Chem..

[58] Belal A., Abdel Gawad N.M., Mehany A.B., Abourehab M.A., Elkady H., Al-Karmalawy A.A., Ismael A.S. (2022). Design, synthesis and molecular docking of new fused 1 H-pyrroles, pyrrolo [3, 2-d] pyrimidines and pyrrolo [3, 2-e][1,4] diazepine derivatives as potent EGFR/CDK2 inhibitors. J. Enzym. Inhib. Med. Chem..

[59] Belal A., Elanany M.A., Santali E.Y., Al-Karmalawy A.A., Aboelez M.O., Amin A.H., Abdellattif M.H., Mehany A.B., Elkady H. (2022). Screening a Panel of Topical Ophthalmic Medications against MMP-2 and MMP-9 to Investigate Their Potential in Keratoconus Management. Molecules.

[60] Abdallah A.E., Alesawy M.S., Eissa S.I., El-Fakharany E.M., Kalaba M.H., Sharaf M.H., Shama N.M.A., Mahmoud S.H., Mostafa A., Al-Karmalawy A.A. (2021). Design and synthesis of new 4-(2-nitrophenoxy) benzamide derivatives as potential antiviral agents: Molecular modeling and in vitro antiviral screening. New J. Chem..

[61] Park J.H., Liu Y., Lemmon M.A., Radhakrishnan R. (2012). Erlotinib binds both inactive and active conformations of the EGFR tyrosine kinase domain. Biochem. J.

[62] Sogabe S., Kawakita Y., Igaki S., Iwata H., Miki H., Cary D.R., Takagi T., Takagi S., Ohta Y., Ishikawa T. (2012). Structure-based approach for the discovery of pyrrolo [3, 2-d] pyrimidine-based EGFR T790M/L858R mutant inhibitors. ACS Med. Chem. Lett..

[63] Husein D.Z., Hassanien R., Khamis M. (2021). Cadmium oxide nanoparticles/graphene composite: Synthesis, theoretical insights into reactivity and adsorption study. RSC Adv..

[64] Wang T., Husein D.Z. (2022). Novel synthesis of multicomponent porous nano-hybrid composite, theoretical investigation using DFT and dye adsorption applications: Disposing of waste with waste. Environ. Sci. Pollut. Res..

[65] Illustrated Glossary of Organic Chemistry. Electrostatic Potential Map. http://www.chem.ucla.edu/~harding/IGOC/E/electrostatic_potential_map.html.

[66] Norinder U., Bergström C.A. (2006). Prediction of ADMET properties. ChemMedChem Chem. Enabling Drug Discov..

[67] Dearden J.C. (2003). In silico prediction of drug toxicity. J. Comput.-Aided Mol. Des..

[68] Idakwo G., Luttrell J., Chen M., Hong H., Zhou Z., Gong P., Zhang C. (2018). A review on machine learning methods for in silico toxicity prediction. J. Environ. Sci. Health Part C.

[69] Kruhlak N., Benz R., Zhou H., Colatsky T. (2012). (Q) SAR modeling and safety assessment in regulatory review. Clin. Pharmacol. Ther..

[70] Zygmunt M., Zmudzki P., Chlon-Rzepa G., Sapa J., Pawlowski M. (2014). Synthesis and Analgesic Activity of 3, 7-dimethylpurine-2, 6-dion-1-yl Derivatives of Acetic and Butanoic Acid. Lett. Drug Des. Discov..

[71] Elwan A., Abdallah A.E., Mahdy H.A., Dahab M.A., Taghour M.S., Elkaeed E.B., Mehany A.B., Nabeeh A., Adel M., Alsfouk A.A. (2022). Modified Benzoxazole-Based VEGFR-2 Inhibitors and Apoptosis Inducers: Design, Synthesis, and Anti-Proliferative Evaluation. Molecules.

[72] Nossier E.S., Alasfoury R.A., Hagras M., El-Manawaty M., Sayed S.M., Ibrahim I.M., Elkady H., Eissa I.H., Elzahabi H.S.A. (2022). Modified pyrido[2,3-d]pyrimidin-4(3H)-one derivatives as EGFRWT and EGFRT790M inhibitors: Design, synthesis, and anti-cancer evaluation. J. Mol. Struct..

[73] Alsaif N.A., Taghour M.S., Alanazi M.M., Obaidullah A.J., Al-Mehizia A.A., Alanazi M.M., Aldawas S., Elwan A., Elkady H. (2021). Discovery of new VEGFR-2 inhibitors based on bis ([1, 2, 4] triazolo)[4, 3-a: 3’, 4’-c] quinoxaline derivatives as anticancer agents and apoptosis inducers. J. Enzym. Inhib. Med. Chem..

[74] Alanazi M.M., Eissa I.H., Alsaif N.A., Obaidullah A.J., Alanazi W.A., Alasmari A.F., Albassam H., Elkady H., Elwan A. (2021). Design, synthesis, docking, ADMET studies, and anticancer evaluation of new 3-methylquinoxaline derivatives as VEGFR-2 inhibitors and apoptosis inducers. J. Enzym. Inhib. Med. Chem..

[75] Taghour M.S., Mahdy H.A., Gomaa M.H., Aglan A., Eldeib M.G., Elwan A., Dahab M.A., Elkaeed E.B., Alsfouk A.A., Khalifa M.M. (2022). Benzoxazole derivatives as new VEGFR-2 inhibitors and apoptosis inducers: Design, synthesis, in silico studies, and antiproliferative evaluation. J. Enzym. Inhib. Med. Chem..

[76] Elkaeed E.B., Eissa I.H., Elkady H., Abdelalim A., Alqaisi A.M., Alsfouk A.A., Elwan A., Metwaly A.M. (2022). A Multistage In Silico Study of Natural Potential Inhibitors Targeting SARS-CoV-2 Main Protease. Int. J. Mol. Sci..

[77] Elkaeed E.B., Youssef F.S., Eissa I.H., Elkady H., Alsfouk A.A., Ashour M.L., El Hassab M.A., Abou-Seri S.M., Metwaly A.M. (2022). Multi-step in silico discovery of natural drugs against COVID-19 targeting main protease. Int. J. Mol. Sci..

[78] Elkaeed E.B., Yousef R.G., Elkady H., Gobaara I.M.M., Alsfouk B.A., Husein D.Z., Ibrahim I.M., Metwaly A.M., Eissa I.H. (2022). Design, Synthesis, Docking, DFT, MD Simulation Studies of a New Nicotinamide-Based Derivative: In Vitro Anticancer and VEGFR-2 Inhibitory Effects. Molecules.

[79] Gaussian. https://gaussian.com/citation/.

[80] Frisch M., Trucks G.W., Schlegel H.B., Scuseria G.E., Robb M.A., Cheeseman J.R., Scalmani G., Barone V., Mennucci B., Petersson G. (2009). Gaussian 09.

[81] Taghour M.S., Elkady H., Eldehna W.M., El-Deeb N.M., Kenawy A.M., Elkaeed E.B., Alsfouk A.A., Alesawy M.S., Metwaly A.M., Eissa I.H. (2022). Design and synthesis of thiazolidine-2, 4-diones hybrids with 1, 2-dihydroquinolones and 2-oxindoles as potential VEGFR-2 inhibitors: In-vitro anticancer evaluation and in-silico studies. J. Enzym. Inhib. Med. Chem..

