# Supplementary material for: New Anticancer Theobromine Derivative Targeting EGFRWT and EGFRT790M: Design, Semi-Synthesis, In Silico, and In Vitro Anticancer Studies
Source: Molecules. 2022 Sep 9;27(18):5859. doi: 10.3390/molecules27185859 (PMC9500845; doi:10.3390/molecules27185859)
Supplement: Supplementary file 1 [file molecules-27-05859-s001.zip › molecules-1868986-supplementary.pdf]

Supporting data

New anticancer theobromine derivative targeting $EGFR^{WT}$ and $EGFR^{T790M}$: design, semi-synthesis, *in silico* and *in vitro* anticancer studies

Eslam B. Elkaeed[a], Reda G.Yousef [b], Hazem Elkady[b], Aisha A. Alsfouk[c], Dalal Z. Husein[d], Ibrahim M. Ibrahim[e], Ahmed M. Metwaly[f,g]* Ibrahim H. Eissa[b]*

[a] Department of Pharmaceutical Sciences, College of Pharmacy, AlMaarefa University, Riyadh 13713, Saudi Arabia.

[b] Pharmaceutical Medicinal Chemistry & Drug Design Department, Faculty of Pharmacy (Boys), Al-Azhar University, Cairo11884, Egypt.

[c] Department of Pharmaceutical Sciences, College of Pharmacy, Princess Nourah bint Abdulrahman University, P.O. Box 84428, Riyadh 11671, Saudi Arabia

[d] Chemistry Department, Faculty of Science, New Valley University, El-Kharja 72511, Egypt.

[e] Biophysics Department, Faculty of Science, Cairo University. Cairo 12613, Egypt.

[f] Pharmacognosy and Medicinal Plants Department, Faculty of Pharmacy (Boys), Al-Azhar University, Cairo 11884, Egypt.

[g] Biopharmaceutical Products Research Department, Genetic Engineering and Biotechnology Research Institute, City of Scientific Research and Technological Applications (SRTA-City), Alexandria, Egypt.

Content

**Method**	**Molecular Docking**
	**MD Simulations**
	**MM-GBSA**
	**DFT**
	**ADMET studies**
	**Toxicity studies**
	**Synthesis**
	***In vitro* assays**
**Spectral Data**	**IR of compound 5**
	**^{1}H and ^{13}C NMR of compound 5**
**Toxicity report**	

Method

- **Molecular Docking studies**

Crystal structure of wild ($EGFR^{WT}$; PDB: 4HJO) and mutant ($EGFR^{T790M}$; PDB: 3W2O) types of EGFR-TK were obtained from Protein Data Bank. The docking investigation was accomplished using MOE2014 software. At first, the crystal structure of EGFR was prepared by removing water molecules. Only one chain was retained besides the co-crystallized ligand (erlotinib). Then, the selected chain was protonated and subjected to the minimization of the energy process. Next, the active site of the target protein was defined.

Structures of the synthesized compound and erlotinib were drawn using ChemBioDraw Ultra 14.0 and saved as MDL-SD format. Such a file was opened using MOE to display the 3D structures which were protonated and subjected to energy minimization. Formerly, validation of the docking process was performed by docking the co-crystallized ligand against the isolated pocket of the active site. The produced RMSD value indicated the validity of the process. Finally, docking of the tested compounds was done through the dock option inserted in compute window. For each docked molecule, 30 docked poses were produced using ASE for scoring function and force field for refinement. The results of the docking process were then visualized using Discovery Studio 4.0 software.

- **Molecular Dynamic Simulation**

Molecular Dynamic Simulation:

In this study, we performed a classical molecular dynamic (MD) simulation to study the stability and the binding affinity of the protein-COMPOUND 5 complex. To prepare the system, the solution builder module in the CHARMM-GUI web server was utilized to generate the necessary files (1–4). First, the complex was uploaded as a PDB file, solvated using the transferable intermolecular potential 3P (TIP3P) water model in a cubic box with a padding of 1 nm, and neutralized with Na^+ and Cl^- ions to the physiological concentration of 0.154 M of salt concentration. The CHARMM36m force field was used to parameterize the amino acids of the protein, neutralizing ions, and water molecules while the CHARMM general force field (CGenFF) tool implemented in CHARMM-GUI was utilized to parameterize the compound

COMPOUND 5. GROMACS 2021 (5) was utilized as an MD engine to perform the simulation with periodic boundary conditions (PBC) applied to the system. Before the production run, the system must be minimized and equilibrated therefore, a minimization step using the steepest descent algorithm with a max force set to 100 $KJ.mol^{-1}.nm^{-1}$ as a convergence criterion was initiated followed by two equilibration steps. The first equilibration step was in a constant number of atoms, constant volume, and constant temperature (NVT) ensemble while the second step was in a constant number of atoms, constant pressure, and constant temperature (NPT) ensemble. The temperature was set to 310 K and maintained using the V-rescale algorithm (6) during the equilibration. On the other hand, the pressure was set to 1 atmospheric pressure and maintained using the Berendsen barostat. Finally, the production run was in an NVT ensemble for 100 ns and the temperature was maintained using the Nose-Hoover thermostat (7). In each step, the bond lengths of hydrogen-bonded atoms were constrained using LINear Constraint Solver (LINCS) algorithm (8). The calculation of the electrostatics was performed using Particle Mesh Ewald (PME) (9) algorithm with a cutoff of 1.2 nm. To integrate the Newtonian equations of motion, the leap-frog algorithm was used with a time step of 1 femtosecond for the equilibration steps and 2 femtoseconds for the production step. The production run was saved for every 100 picoseconds with a total of 1000 frames. Before analyzing the trajectory, the PBC was removed using the trjconv tool in GROMACS. The analysis of the production trajectory was performed using VMD TK scripts (10). Root mean square deviation (RMSD) for the protein alone, the compound COMPOUND 5, and the complex were measured. In addition, the root mean square fluctuation (RMSF), the solvent accessible surface area (SASA), the radius of gyration (RoG), the number of hydrogen bonds, and the distance between the center of mass of the compound COMPOUND 5 and the center of mass of the protein were measured. Afterward,

the trajectory was clustered using TTClust (11) to get a representative frame for each cluster. First, backbone alignment was performed before determining the optimum number of clusters using the elbow method. For each representative frame, protein Ligand interaction profiler (PLIP) was used to detect the number and types of interactions (12).

Binding free energy calculation using MM-GBSA:

To find the binding affinity, the gmx_MMPBSA package was used with Molecular Mechanics Generalized Born Surface Area (MM-GBSA) algorithm. In addition, decomposition analysis was calculated to get the binding energies of amino acids within 1 nm around the compound COMPOUND 5 (13,14). The salt concentration and the method of solvation (igb) were set to 0.154 M and 5, respectively. The internal and external dielectric constants were set to 1.0 and 80.0, respectively, and other options were set as default. MM-GBSA approach is depicted in Equation 1.

$$\Delta G = < G_{complex} - G_{receptor} - G_{compound} > \qquad \text{Equation 1}$$

Where < > represents the average of the enclosed free energies of the complex, he receptor, and compound over the frames used in calculation. In our approach, we used the whole trajectory (a total of 1000 frames). Different energy terms can be calculated according to Equations 2 to 6 as follows:

$$\Delta G_{binding} = \Delta H - T\Delta S \qquad \text{Equation 2}$$

$$\Delta H = \Delta E_{gas} + \Delta E_{sol} \qquad \text{Equation 3}$$

$$\Delta E_{gas} = \Delta E_{ele} + \Delta E_{vdW} \qquad \text{Equation 4}$$

$$\Delta E_{solv} = E_{GB} + E_{SA} \qquad \text{Equation 5}$$

$$E_{SA} = \gamma . SASA \qquad \text{Equation 6}$$

Where:

ΔH is the enthalpy which can be calculated from gas-phase energy (E_{gas}) and solvation-free energy (E_{sol}). TΔS is the entropy contribution to the free binding energy. E_{gas} is composed of electrostatic and van der Waals terms; E_{ele}, E_{vdW}, respectively. E_{sol} can be calculated from the polar solvation energy (E_{GB}) and nonpolar solvation energy (E_{SA}) which is estimated from the solvent-accessible surface area (15,16).

- **Density Function Theory (DFT) calculations**

The Gaussian 09 program was used to perform the quantum chemistry calculations using the DFT method. GaussianView5 was used to display all of the data files. The density function theory (DFT) at 6-311G++(d,p) basis set/B3LYP approach was utilized to optimize organic chemical structure of the compound under investigation and Chem3D 15.0 software was used to create the original chemical structures. Both the Total Electron Density (TED) and the Electrostatic Surface (ESP) maps were examined at the same theoretical level. GaussSum3.0 software was used to compute and evaluate the total density of state (TDOS) for the optimized log file.

Equations of Koopmans' theory: The chemical potential (μ), maximal charge acceptance (ΔN_{max}), global hardness (η), energy change (ΔE), electronegativity (χ), the global softness (σ), electrophilicity index (ω), ionization potential (IP) and electron affinity (EA)

$IP = -E_{HOMO}$

$EA = -E_{LUMO}$

μ=

(IP+EA)/2

η= (IP-EA)

χ= - η

$\omega = \mu^2/(2$

$\eta)$ σ= 1/

η

ΔN= -(μ/η)

$\Delta E = -\omega$

$E_{gap} = E_{LUMO} – E_{HOMO}$

- ***In silico* ADMET studies**

ADMET descriptors (absorption, distribution, metabolism, excretion and toxicity) of the compounds were determined using Discovery studio 4.0. At first, the CHARMM force field was applied then the compounds were prepared and minimized according to the preparation of small molecule protocol. Then ADMET descriptors protocol was applied to carry out these studies

- ***In silico* toxicity studies**

The toxicity parameters of the synthesized compounds were calculated using Discovery studio 4.0. erlotinib was used as a reference drug. At first, the CHARMM force field was applied then the compounds were prepared and minimized according to the preparation of small molecule protocol. Then different parameters were calculated from the toxicity prediction (extensible) protocol.

- **Chemistry**

The melting point were carried out by open capillary method on a Gallen kamp Melting point apparatus. The infrared spectra were recorded on pye Unicam SP 1000 IR spectrophotometer using potassium bromide disc technique. Proton magnetic resonance ^{1}HNMR spectra were recorded on a Bruker 400 Megahertz-nuclear magnetic resonance (400 MHZ-NMR) spectrophotometer. Carbon-13 (C13) nuclear magnetic resonance (^{13}CNMR) spectra were recorded on a Bruker 100 Megahertz-nuclear magnetic resonance (100 MHZ-NMR) spectrophotometer. Tetramethylsilane (TMS) was used as internal standard and chemical shifts were measured in δ scale one part per million (ppm). The reactions were monitored by thin-layer chromatography (TLC) using TLC sheets precoated with UV fluorescent silica gel Merck 60 F254 plates and were visualized using ultraviolet (UV) lamp and different solvents as mobile phases.

General procedure for the synthesis of compound 5

To a solution of the potassium salt of 3,7-dimethyl-3,7-dihydro-1*H*-purine-2,6-dione **2** (0.001 mol, 0.25g) in dry DMF (10 mL), *N*-benzyl-2-chloroacetamide **4** (0.001 mol, 0.21g) was added and the mixture was heated using water-bath for 5 h. The reaction mixture was then poured onto ice-water (200 mL) and stirred for 30 min. The formed precipitate was filtered, washed with water and crystallized from ethanol to get the final target compound **5**.

***N*-Benzyl-2-(3,7-dimethyl-2,6-dioxo-2,3,6,7-tetrahydro-1*H*-purin-1-yl)acetamide (5)**

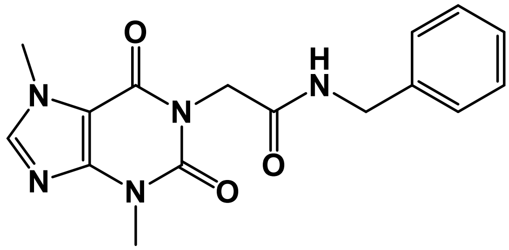

Off-white crystal **(**yield, 84 %); m. p. = 257-259 °C; IR (KBr) *v* cm^{-1}: 3282, 3108 (NH), 2929 (CH aliphatic), 1711, 1656 (C=O); ^{1}H NMR (400 MHz, DMSO-d_6) δ 8.61 (t, *J* = 5.9 Hz, 1H), 8.07 (s, 1H), 7.37 – 7.31 (m, 2H), 7.26 (d, *J* = 7.5 Hz, 3H), 4.52 (s, 2H), 4.30 (d, *J* = 5.9 Hz, 2H), 3.90 (s, 3H), 3.44 (s, 3H); ^{13}C NMR (101 MHz, DMSO-d_6) δ 167.50, 154.73, 151.39, 148.97, 143.53, 139.72, 128.75 (2C), 127.57 (2C), 127.25, 107.18, 43.44, 42.52, 33.67, 29.91. For $C_{16}H_{17}N_5O_3$ (327.34).

- **Biological evaluations**

1- Mammalian cell lines culture

A549 and HCT 116 cell lines were cultured on DMEM media. The cultured media were supplemented with 200 mM L-glutamine, 10.0% fetal bovine serum (Lonza), and 1.0% penicillin/streptomycin. Cells were seeded into 25.0 cm tissue culture flasks and incubated at 37°C in a 5.0% CO_2 incubator for 24 h or till confluency.

2- Safety assay

The safety profiles of the tested compounds were checked on one non-cancerous cell line (W138) to determine the treatments concentrations that do not depict toxic effects against the

tested cells. A portion of 100.0 µl of 6×10^4 cell/ml cells was seeded into each well of a 96-well plate and then the plates were incubated at 37°C in a humidified 5.0% CO_2 incubator for 24 h. At the end of incubation period, the exhausted medium was replaced with 100.0 µl of different concentrations of the designated treatment (prepared in RPMI medium starting from 1.0 mM). The inoculated plates were incubated at the same growth conditions for another 24 h. At the end of incubation, cellular viability was assessed using MTS assay kit (Promega) according to the manual instruction

3- ***In-vitro* anticancer activity**

Anticancer activities of the tested compounds against A549 and HCT 116 cell lines were quantified using MTS assay kit (Promega) as described by the Manufacturer.

4- Selectivity index (SI)

The selectivity index values of the tested compounds on cancer cells were calculated as described by Koch et al., with slight modifications; SI=IC_{50}nc/IC_{50}cc, where IC50nc: the IC50 value of the tested compound on normal cells and IC_{50}cc: IC_{50} of the tested compound on cancer cell line**.**

5- *In vitro* EGFR kinase assay

The synthesized compound was estimated for their *in vitro* inhibition on human EGFR in **MCF- 7** cell line; using ELISA kit. Firstly, a plate was used for the assay had been coated by an antibody specific for human EGFR enzyme, Erlotinib was nominated as a standard EGFR inhibitor. Both standard and sample were added to the wells and incubated overnight at 4 ◦C, then washed. The biotinylated antibody was supplemented and further incubated for 1 h at room temperature. The unreacted, liberated antibody was then washed; followed by addition of HRP-conjugated streptavidin and incubated for 45 min at room temperature. Wells were washed and a TMB substrate solution was added and kept at room temperature for 30 min. Finally, the stop solution was added, and the intensity of the color produced was measured at 450 nm. Concentration-inhibition response curve was established by GraphPad Prism 5.0. The IC_{50} value was calculated as the concentration at which 50% of the cells could survive in comparison to erlotinib.

6- Flow cytometry analysis for cell cycle

To determine the role of the synthesized compound in cell cycle distribution, cell cycle analysis was performed using propidium iodide (PI) staining and flow cytometry analysis for compound **XI**. Flow Cytometry Kit for Cell Cycle Analysis (ab139418_Propidium Iodide Flow Cytometry Kit/BD) was used in this test. A549 cells were treated with compound **XI** (21.99 µM) for 72 h. Then, the cells were fixed in 70% ethanol at 4 °C for 12 h. After that, the cells were washed with cold PBS, incubated with 100 µl RNase A at 37 °C for 30 min, and stained with 400 µl PI in the dark at room temperature for further 30 min. The stained cells were measured using Epics XL-MCL™ Flow Cytometer (Beckman Coulter), and the data were analyzed using Flowing software (version 2.5.1, Turku Centre for Biotechnology, Turku, Finland).

7- Flow cytometry analysis for apoptosis

Flow cytometry cell apoptosis analysis was used to investigate the apoptotic effect of the synthesized compound. A549 cells were treated with compound **XI** (21.99 µM) for 72h, collected by trypsin, centrifuged, washed two successive times with PBS, suspended in 500 µl binding buffer, and double stained with 5 µl Annexin V-FITC and 5 µl PI in the dark at room temperature for 15 min. The stained cells were measured using Epics XL-MCL™ Flow Cytometer and analyzed using Flowing software.

Spectral data

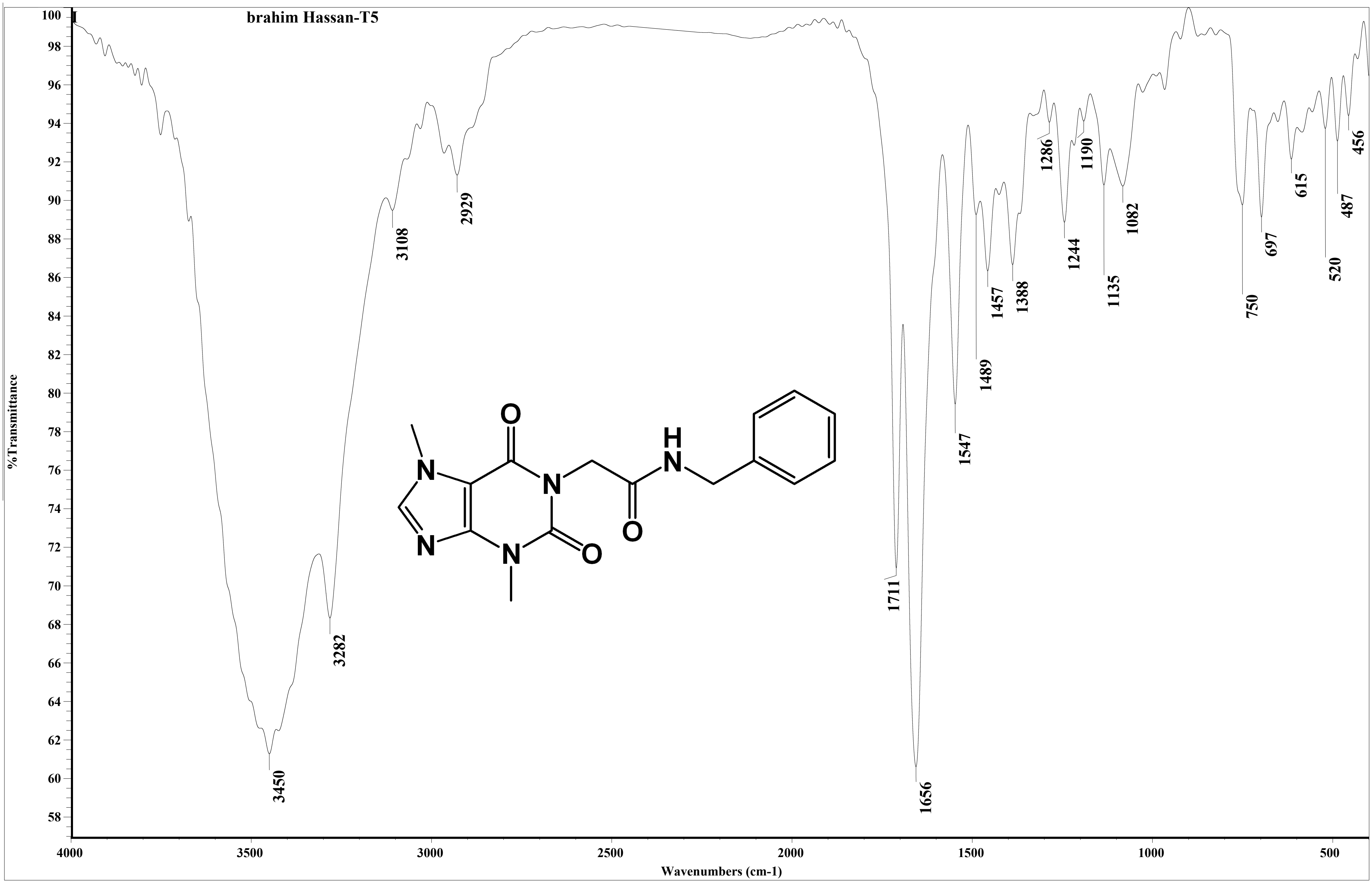

Number of sample scans: 32
Number of background scans: 32
Resolution: 8.000
Sample gain: 1.0
Optical velocity: 0.4747
Aperture: 150.00

ThermoFisher SCIENTIFIC
Mon Jun 20 11:57:37 2022 (GMT+02:00)

Mansoura University
Faculty of Science
Spectral Analysis Unit
unitofspectra@gmail.com

1H NMR of 5

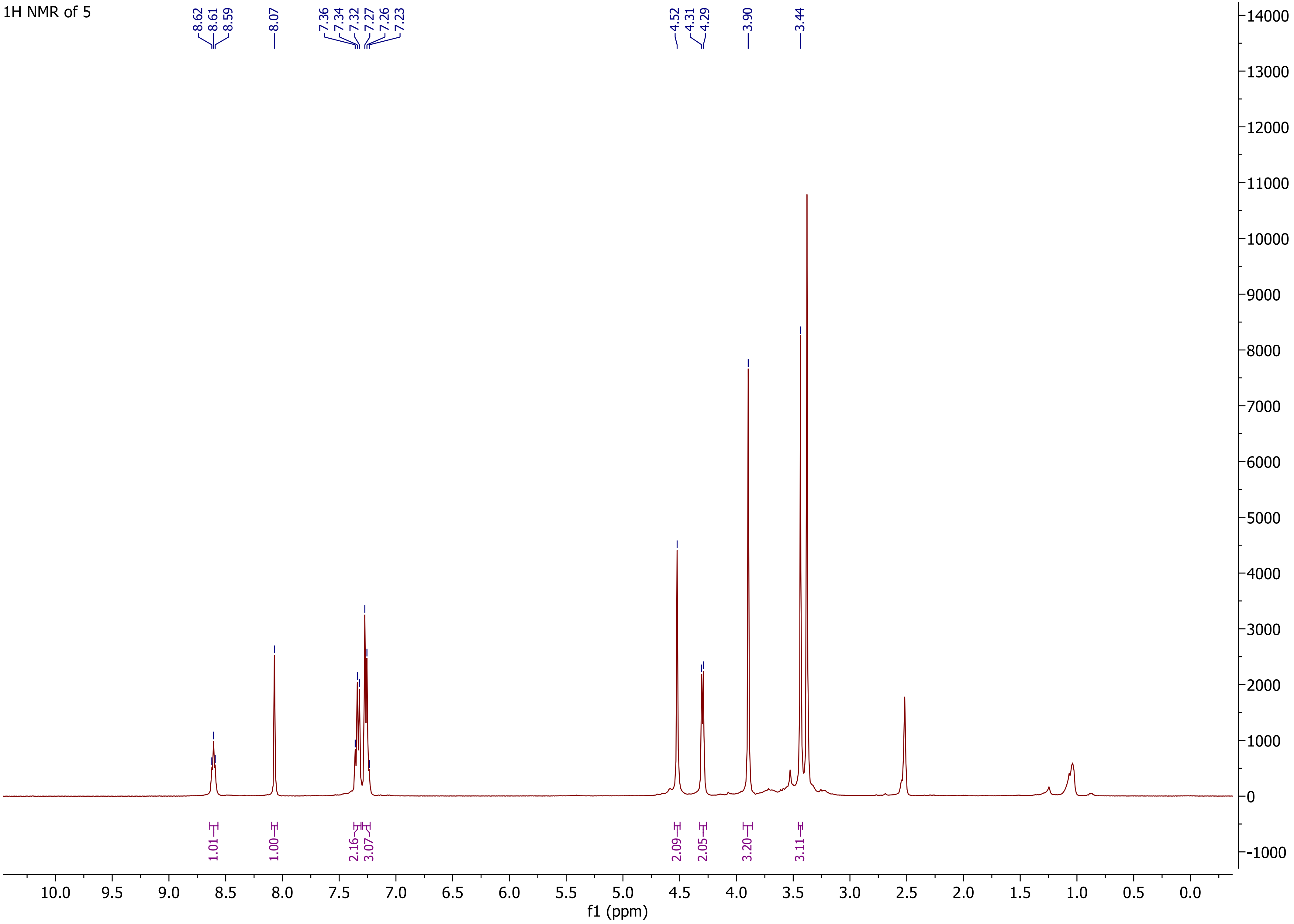

8.62
8.61
8.59
8.07
7.36
7.34
7.32
7.27
7.26
7.23
4.52
4.31
4.29
3.90
3.44
1.01
1.00
2.16
3.07
2.09
2.05
3.20
3.11
14000
13000
12000
11000
10000
9000
8000
7000
6000
5000
4000
3000
2000
1000
0
-1000
10.0
9.5
9.0
8.5
8.0
7.5
7.0
6.5
6.0
5.5
5.0
4.5
4.0
3.5
3.0
2.5
2.0
1.5
1.0
0.5
0.0
f1 (ppm)

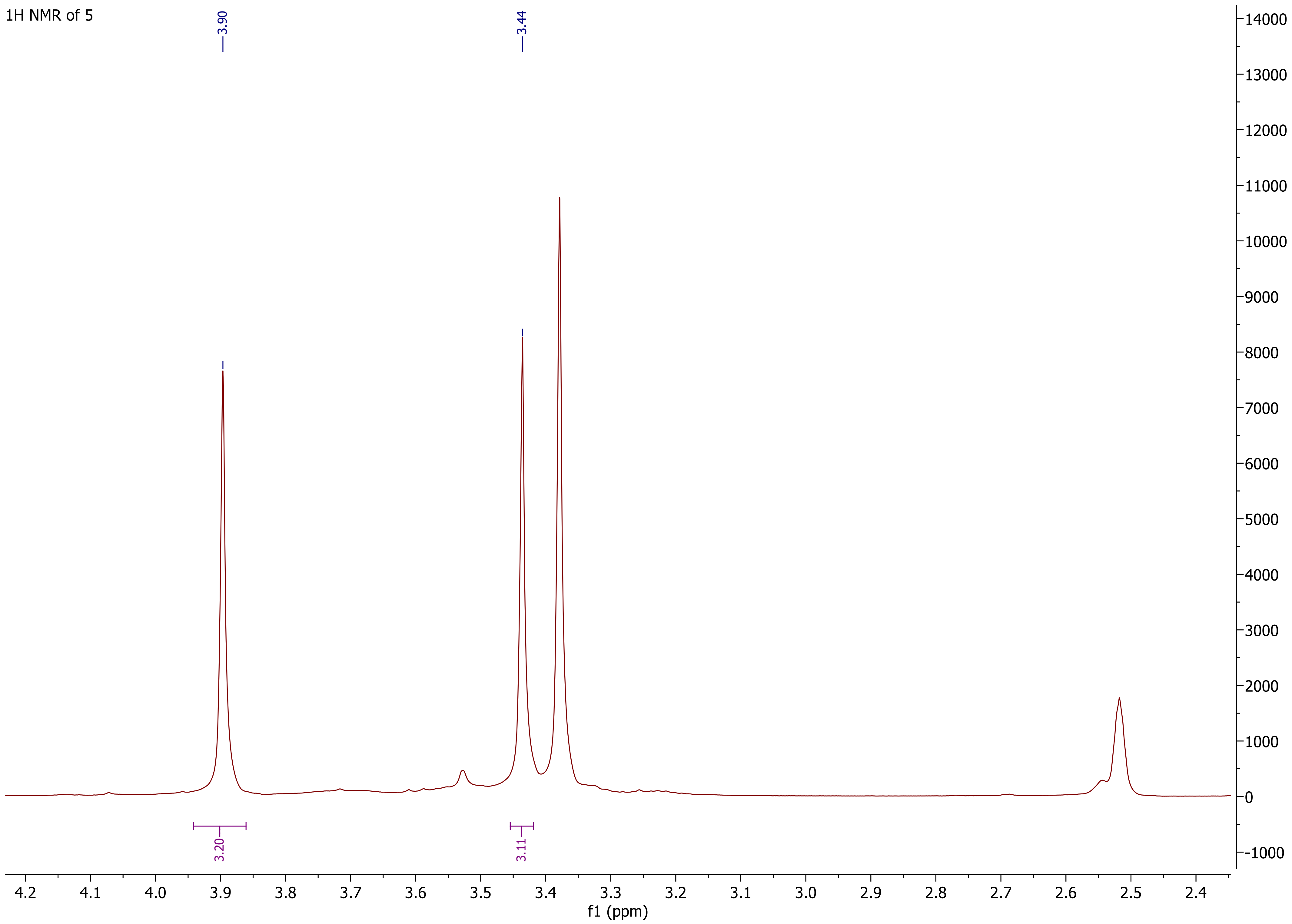
1H NMR of 5
3.90
3.44
3.20
3.11
f1 (ppm)

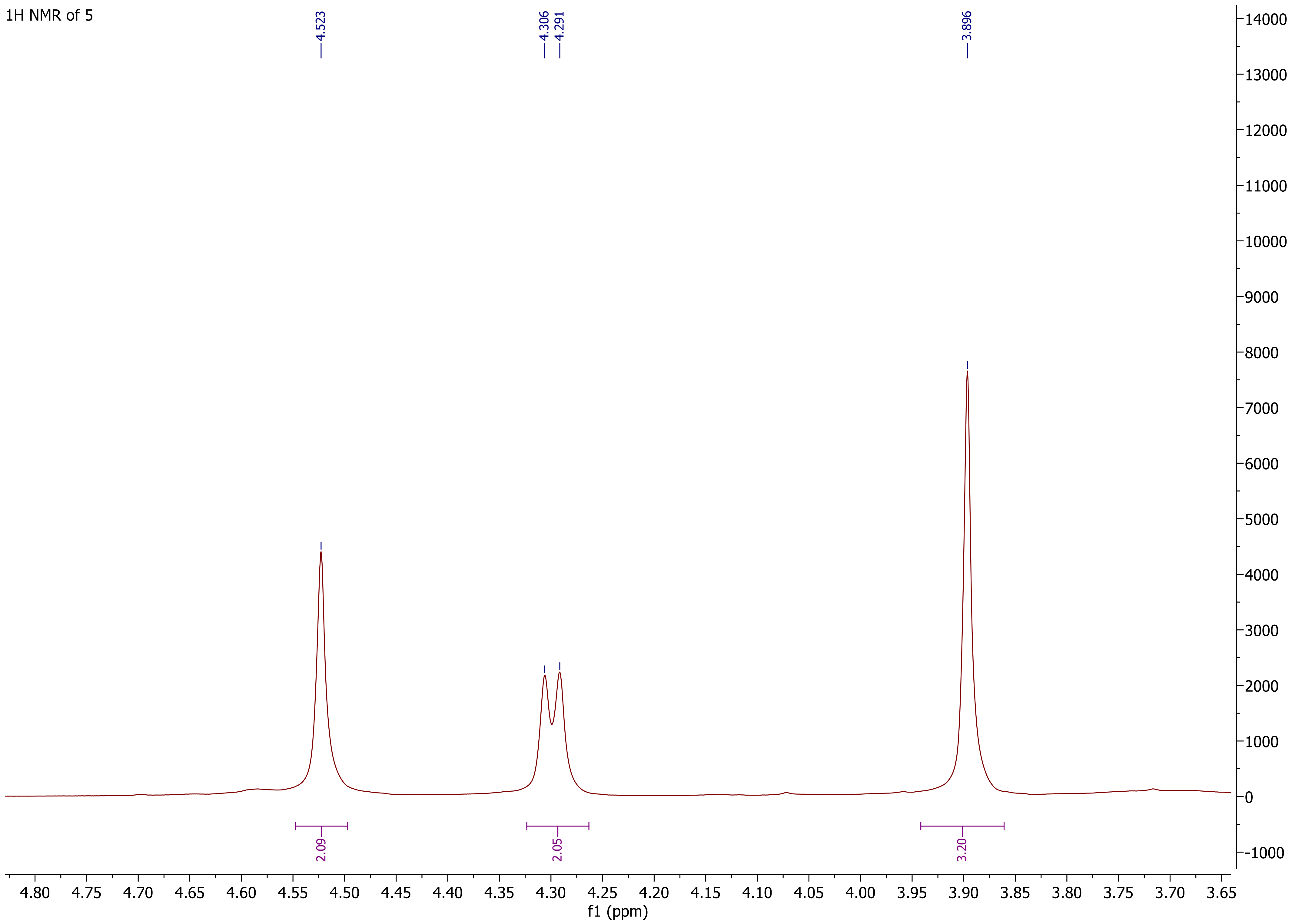
1H NMR of 5
4.523
4.306
4.291
3.896
2.09
2.05
3.20
f1 (ppm)

1H NMR of 5

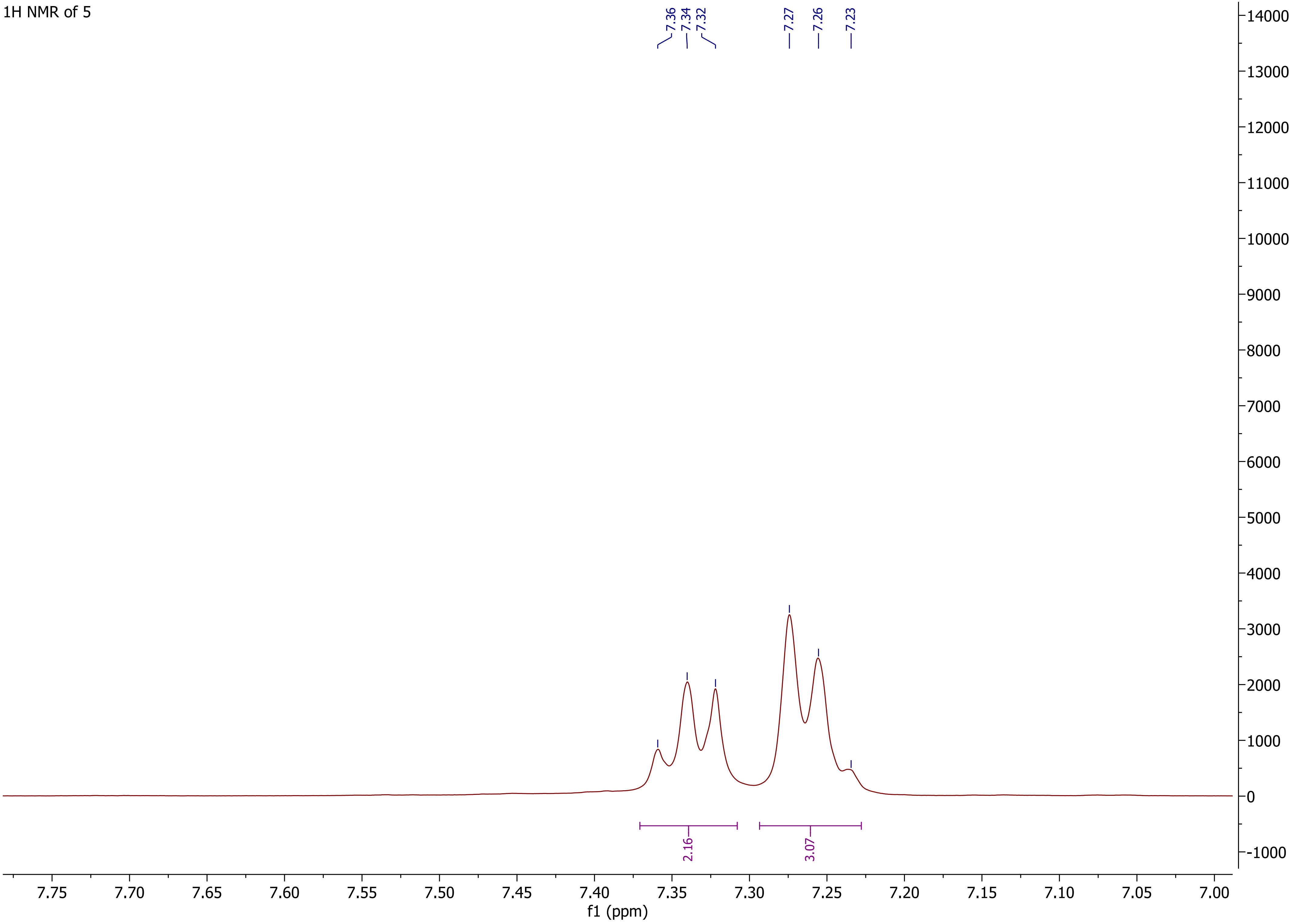
7.36
7.34
7.32
7.27
7.26
7.23
2.16
3.07
14000
13000
12000
11000
10000
9000
8000
7000
6000
5000
4000
3000
2000
1000
0
-1000
7.75
7.70
7.65
7.60
7.55
7.50
7.45
7.40
7.35
7.30
7.25
7.20
7.15
7.10
7.05
7.00
f1 (ppm)

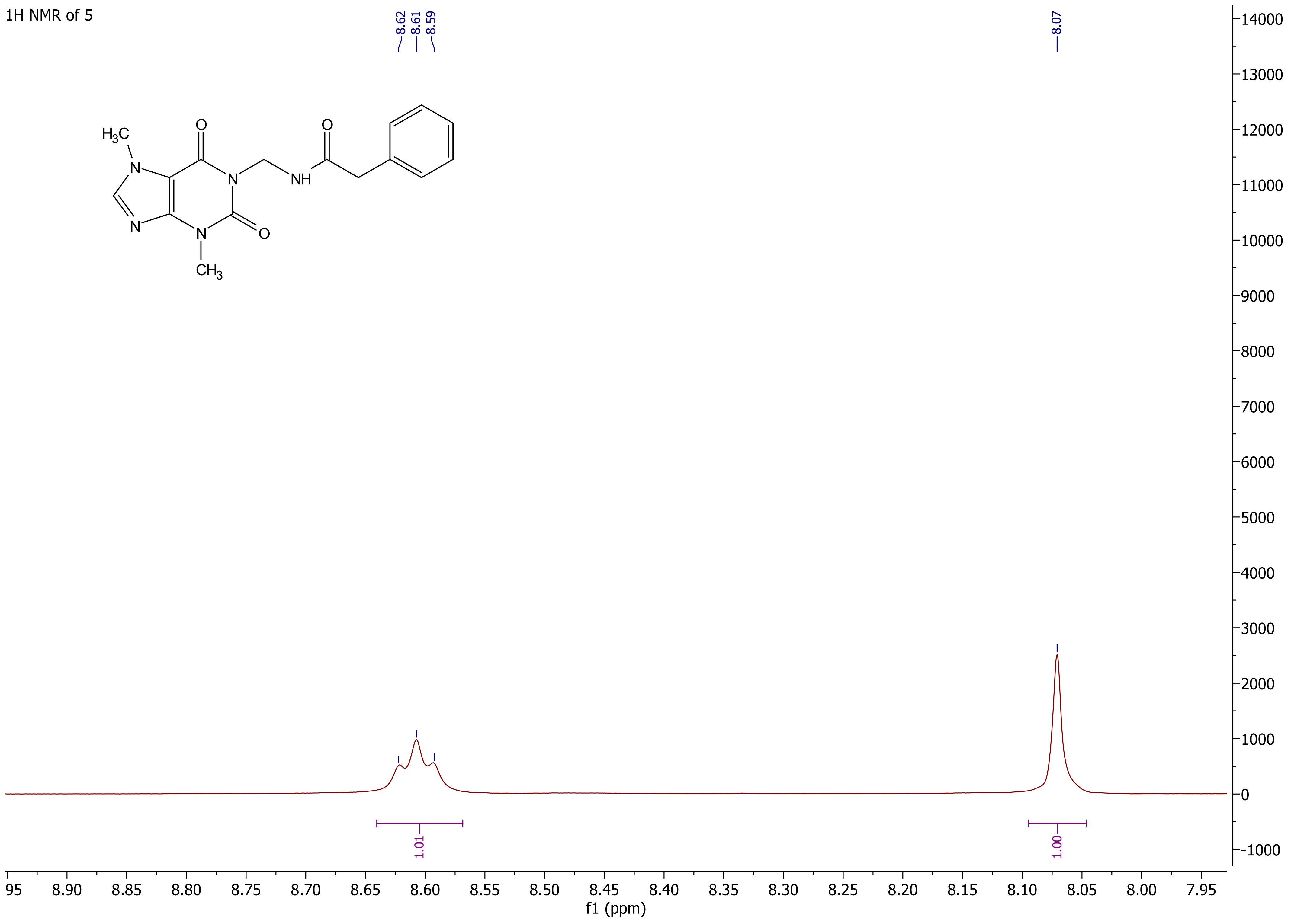
1H NMR of 5
8.62
8.61
8.59
8.07
H3C
O
O
N
N
NH
N
N
O
CH3
1.01
1.00
95 8.90 8.85 8.80 8.75 8.70 8.65 8.60 8.55 8.50 8.45 8.40 8.35 8.30 8.25 8.20 8.15 8.10 8.05 8.00 7.95
f1 (ppm)
14000 13000 12000 11000 10000 9000 8000 7000 6000 5000 4000 3000 2000 1000 0 -1000

13C NMR of 5

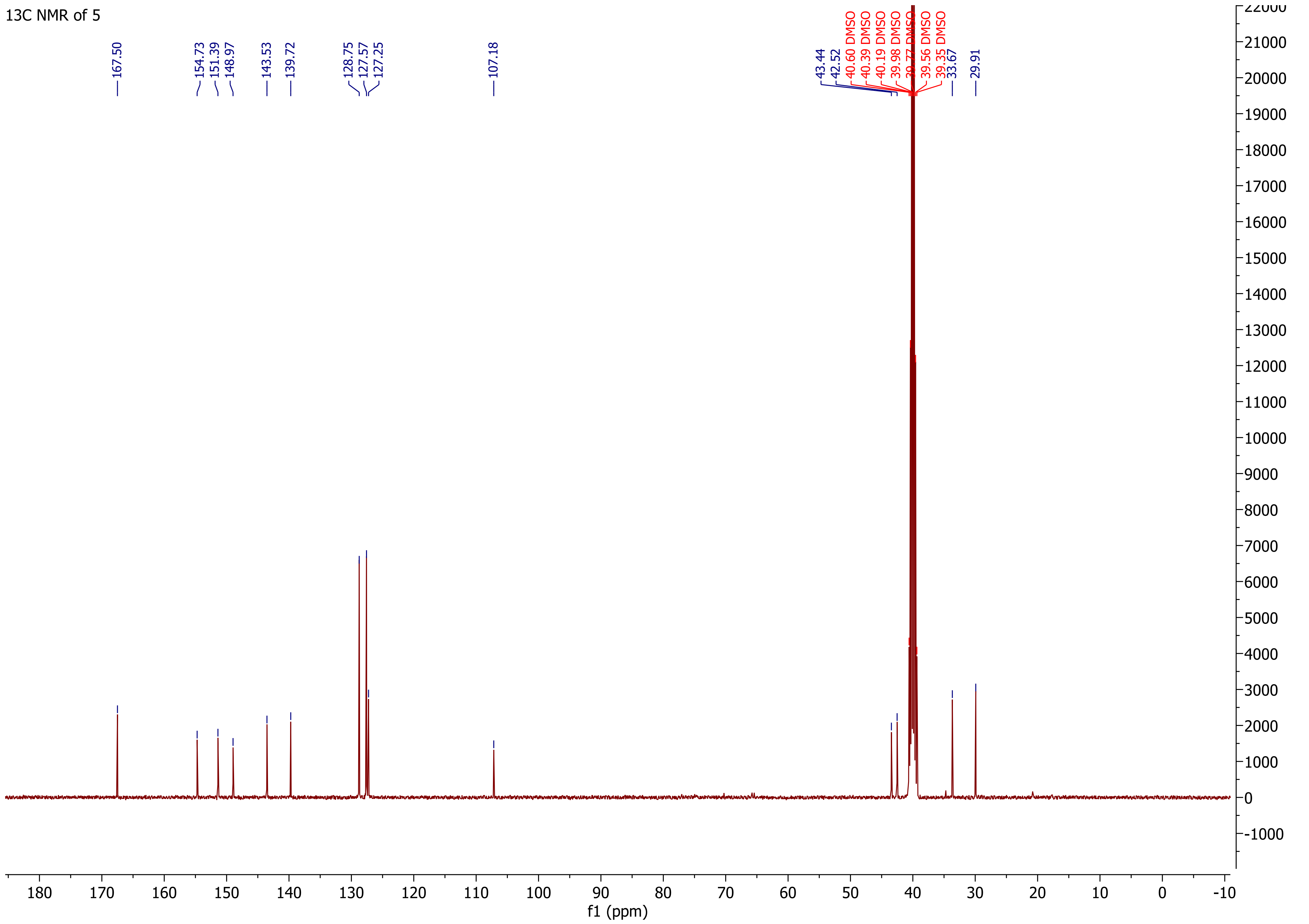
167.50
154.73
151.39
148.97
143.53
139.72
128.75
127.57
127.25
107.18
43.44
42.52
40.60 DMSO
40.39 DMSO
40.19 DMSO
39.98 DMSO
39.77 DMSO
39.56 DMSO
39.35 DMSO
33.67
29.91
f1 (ppm)

13C NMR of 5

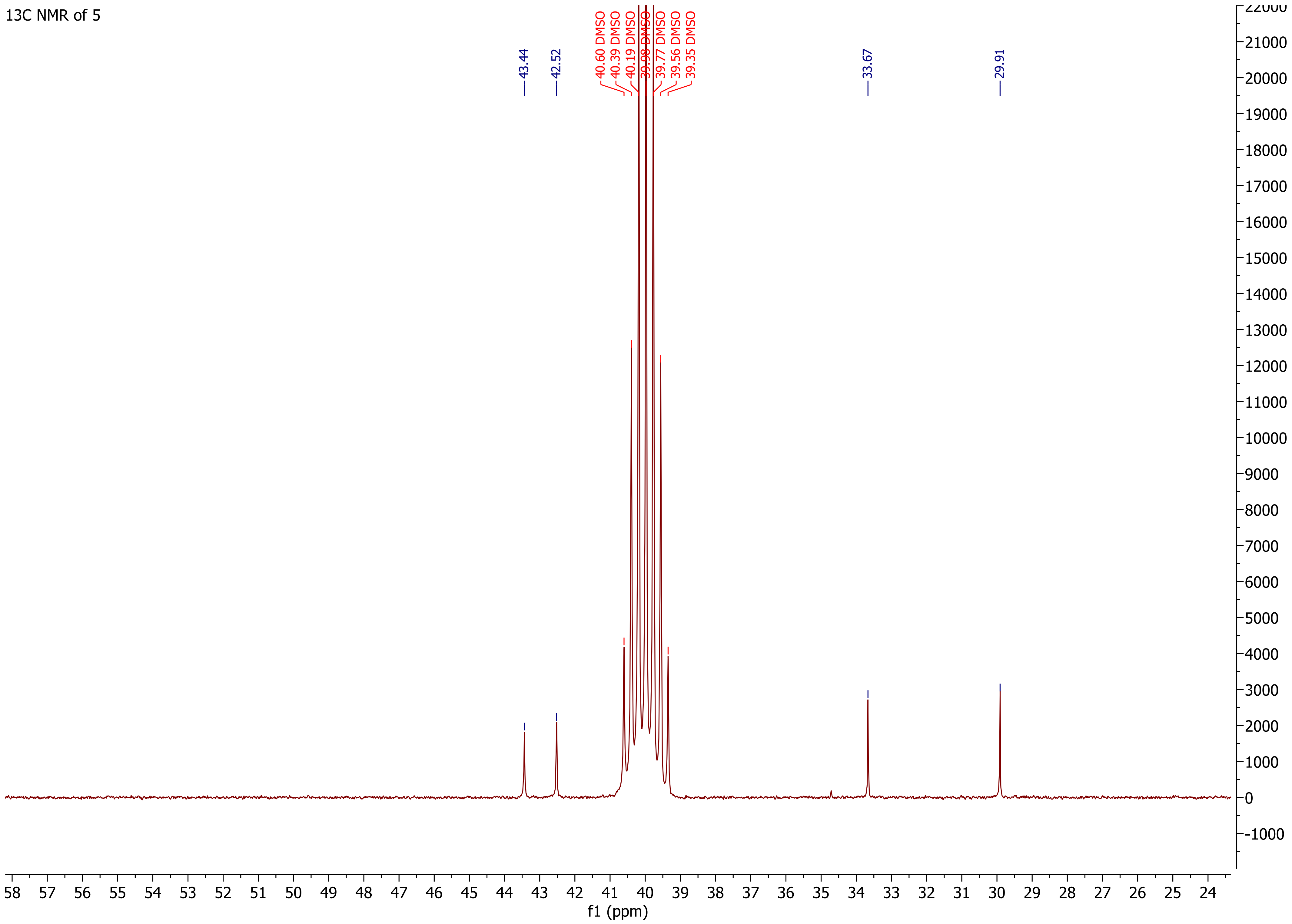

43.44
42.52
40.60 DMSO
40.39 DMSO
40.19 DMSO
39.98 DMSO
39.77 DMSO
39.56 DMSO
39.35 DMSO
33.67
29.91
f1 (ppm)

13C NMR of 5

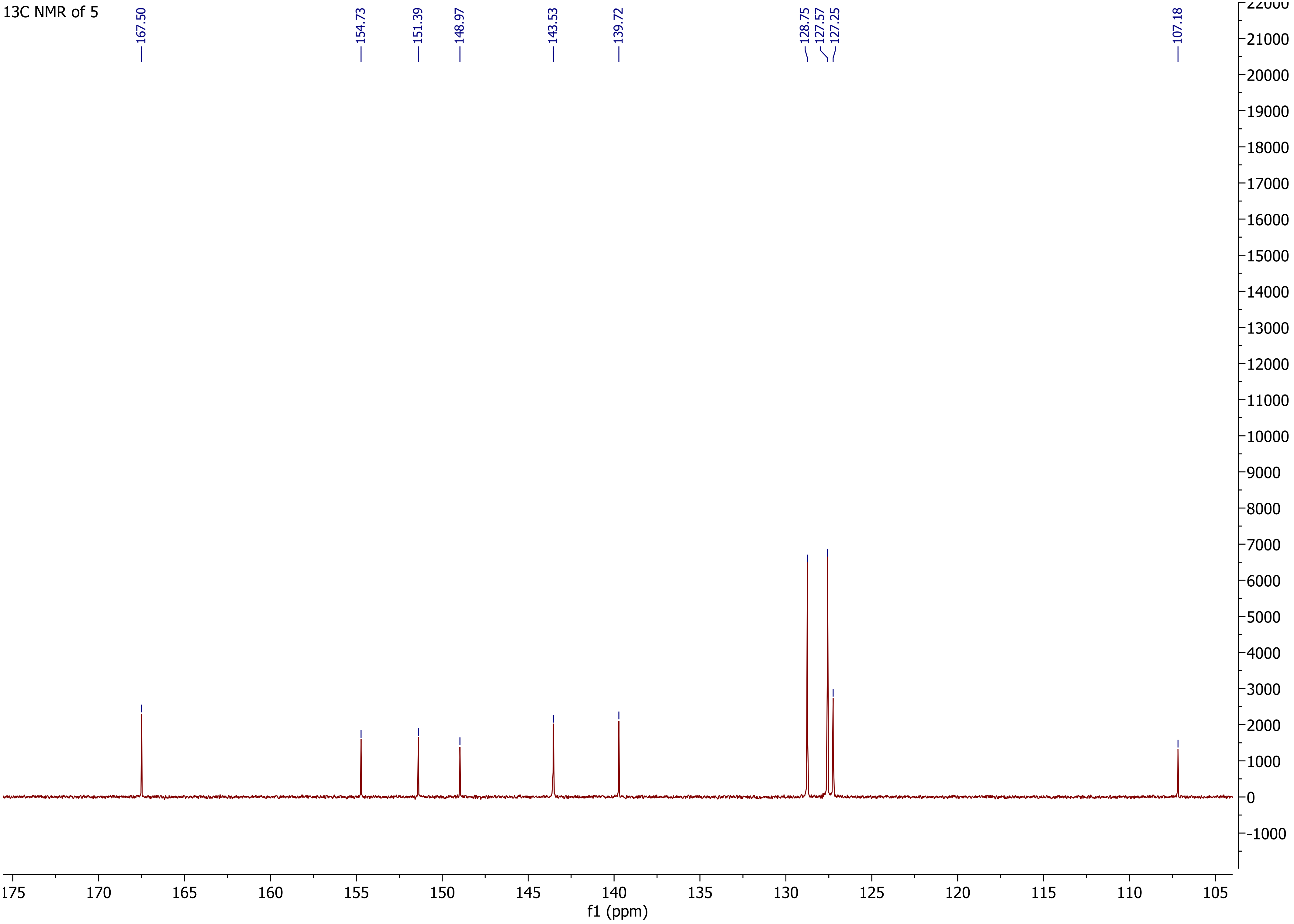

167.50
154.73
151.39
148.97
143.53
139.72
128.75
127.57
127.25
107.18
f1 (ppm)

Toxicity Report

Compound 5

TOPKAT_Ames_Mutagenicity

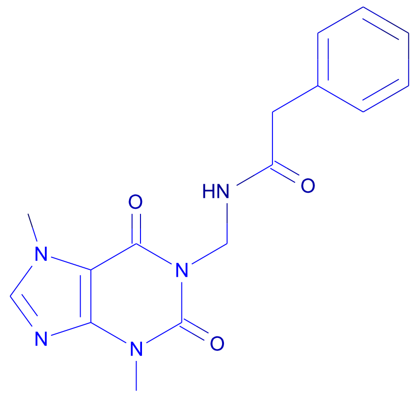

$C_{16}H_{17}N_5O_3$

Molecular Weight: 327.33788

ALogP: 0.773

Rotatable Bonds: 4

Acceptors: 4

Donors: 1

Model Prediction

Prediction: Non-Mutagen

Probability: 0.173

Enrichment: 0.31

Bayesian Score: -14.9

Mahalanobis Distance: 12.1

Mahalanobis Distance p-value: 0.000525

Prediction: Positive if the Bayesian score is above the estimated best cutoff value from minimizing the false positive and false negative rate.
Probability: The esimated probability that the sample is in the positive category. This assumes that the Bayesian score follows a normal distribution and is different from the prediction using a cutoff.
Enrichment: An estimate of enrichment, that is, the increased likelihood (versus random) of this sample being in the category.
Bayesian Score: The standard Laplacian-modified Bayesian score.
Mahalanobis Distance: The Mahalanobis distance (MD) is the distance to the center of the training data. The larger the MD, the less trustworthy the prediction.
Mahalanobis Distance p-value: The p-value gives the fraction of training data with an MD greater than or equal to the one for the given sample, assuming normally distributed data. The smaller the p-value, the less trustworthy the prediciton. For highly non-normal X properties (e.g., fingerprints), the MD p-value is wildly inaccurate.

Structural Similar Compounds

**Name**	**112022-09-0**	**FLUCONAZOLE**	**Scopalamine .HBr .3H2O**
Structure	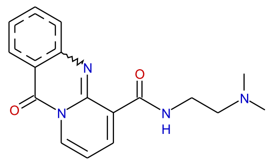	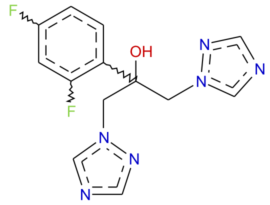	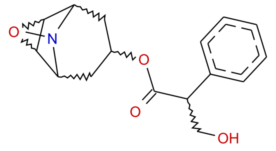
Actual Endpoint	Mutagen	Non-Mutagen	Non-Mutagen
Predicted Endpoint	Mutagen	Non-Mutagen	Non-Mutagen
Distance	0.535	0.554	0.556
Reference	Kazius et. al., J. Med. Chem. (2005) 48, 312-320	NDA # 19-949	Environ. Mol. Mut. 19(21):1992

Model Applicability

Unknown features are fingerprint features in the query molecule, but not found or appearing too infrequently in the training set.

1. All properties and OPS components are within expected ranges.

Feature Contribution

Top features for positive contribution

**Fingerprint**	**Bit/Smiles**	**Feature Structure**	**Score**	**Mutagen in training set**
SCFP_12	136358998	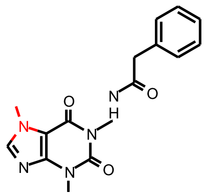 [*]:n(:[*])C	0.455	142 out of 157

SCFP_12	-587777653	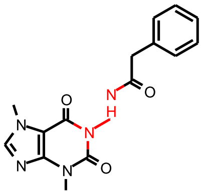 [*]NCN([*])[*]	0.42	4 out of 4
SCFP_12	-443505090	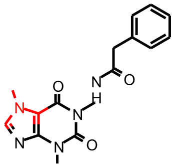 [*][c]1:[*]:[*]:[cH]:n:1C	0.343	176 out of 218

Top Features for negative contribution

Fingerprint	Bit/Smiles	Feature Structure	Score	Mutagen in training set
SCFP_12	1205795299	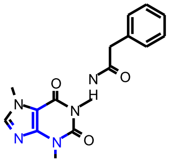 [*]N([*])[c]1:n:[*]:[*]:[c]:1[*]	-1.22	2 out of 16
SCFP_12	1445006032	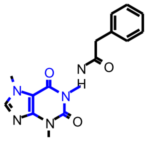 [*]CN1C(=[*])[*][c]2:[*]:[*]:n([*]):[c]:2C1=O	-1.19	0 out of 4
SCFP_12	1731225349	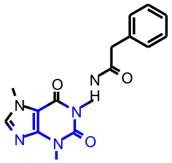 [*]N1[*][c]2:[*]:[*]:n:[c]:2N(C)C1=O	-1.19	0 out of 4

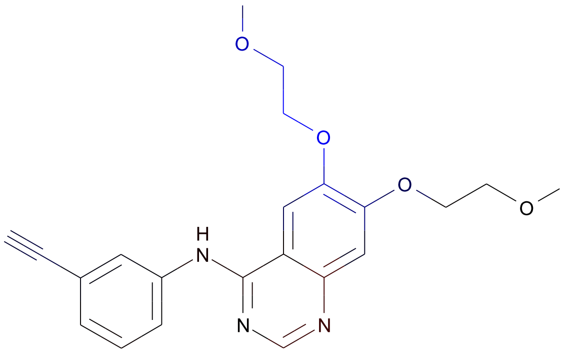

$C_{22}H_{23}N_3O_4$
Molecular Weight: 393.43572
ALogP: 4.309
Rotatable Bonds: 10
Acceptors: 7
Donors: 1

Structural Similar Compounds

Name	Carvedilol	99522-79-9	HYCANTHONE FUROATE
Structure	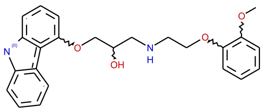	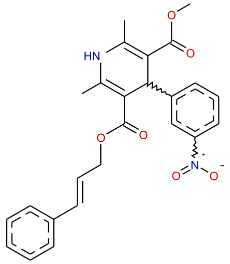	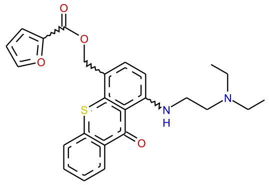
Actual Endpoint	Non-Mutagen	Non-Mutagen	Mutagen
Predicted Endpoint	Non-Mutagen	Non-Mutagen	Mutagen
Distance	0.594	0.598	0.606
Reference	Contrera, J.F., Matthews, E.J., Kruhlak, N.L., and Benz, R.D., Regulatory Toxicology and Pharmacology 2005, 313-323.	Kazius et. al., J. Med. Chem. (2005) 48, 312-320	EMIC

Model Prediction

Prediction: Non-Mutagen
Probability: 0.6
Enrichment: 1.07
Bayesian Score: -5.04
Mahalanobis Distance: 14.6
Mahalanobis Distance p-value: 6.74e-011

Prediction: Positive if the Bayesian score is above the estimated best cutoff value from minimizing the false positive and false negative rate.
Probability: The esimated probability that the sample is in the positive category. This assumes that the Bayesian score follows a normal distribution and is different from the prediction using a cutoff.
Enrichment: An estimate of enrichment, that is, the increased likelihood (versus random) of this sample being in the category.
Bayesian Score: The standard Laplacian-modified Bayesian score.
Mahalanobis Distance: The Mahalanobis distance (MD) is the distance to the center of the training data. The larger the MD, the less trustworthy the prediction.
Mahalanobis Distance p-value: The p-value gives the fraction of training data with an MD greater than or equal to the one for the given sample, assuming normally distributed data. The smaller the p-value, the less trustworthy the prediciton. For highly non-normal X properties (e.g., fingerprints), the MD p-value is wildly inaccurate.

Model Applicability

Unknown features are fingerprint features in the query molecule, but not found or appearing too infrequently in the training set.

1. All properties and OPS components are within expected ranges.

Feature Contribution

Top features for positive contribution

Fingerprint	Bit/Smiles	Feature Structure	Score	Mutagen in training set
SCFP_12	112346096	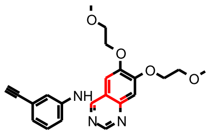 [*][c](:[*]):[c](:[cH]:[*]):[c](:[*]):[*]	0.36	1035 out of 1263

SCFP_12	10	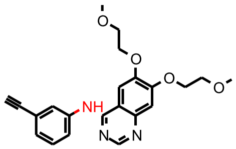 [*]N[*]	0.306	1774 out of 2287
SCFP_12	-1380909229	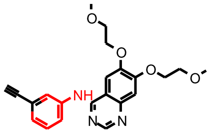 [*]N[c]1:[cH]:[*]:[cH]:[cH]:[cH]:1	0.304	957 out of 1235

Top Features for negative contribution

Fingerprint	Bit/Smiles	Feature Structure	Score	Mutagen in training set
SCFP_12	-1099149596	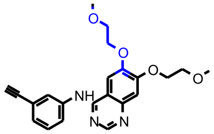 [*]OCCO[c](:[*]):[*]	-1.12	1 out of 9
SCFP_12	-677502852	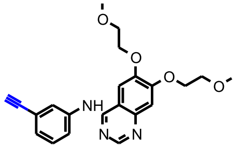 [*]C#C	-0.863	4 out of 19
SCFP_12	-417738003	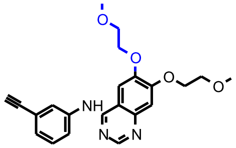 [*]OCCOC	-0.782	12 out of 48

Compound 5

TOPKAT_Developmental_Toxicity_Potential

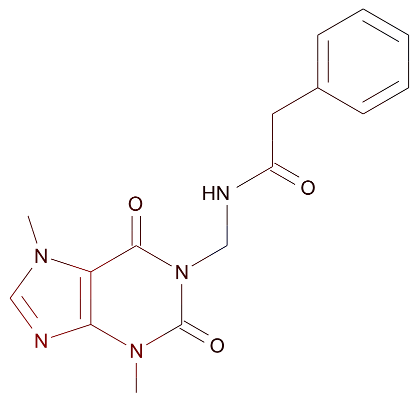

$C_{16}H_{17}N_5O_3$

Molecular Weight: 327.33788

ALogP: 0.773

Rotatable Bonds: 4

Acceptors: 4

Donors: 1

Model Prediction

Prediction: Toxic

Probability: 0.639

Enrichment: 1.21

Bayesian Score: 2.35

Mahalanobis Distance: 8.22

Mahalanobis Distance p-value: 0.498

Prediction: Positive if the Bayesian score is above the estimated best cutoff value from minimizing the false positive and false negative rate.
Probability: The esimated probability that the sample is in the positive category. This assumes that the Bayesian score follows a normal distribution and is different from the prediction using a cutoff.
Enrichment: An estimate of enrichment, that is, the increased likelihood (versus random) of this sample being in the category.
Bayesian Score: The standard Laplacian-modified Bayesian score.
Mahalanobis Distance: The Mahalanobis distance (MD) is the distance to the center of the training data. The larger the MD, the less trustworthy the prediction.
Mahalanobis Distance p-value: The p-value gives the fraction of training data with an MD greater than or equal to the one for the given sample, assuming normally distributed data. The smaller the p-value, the less trustworthy the prediciton. For highly non-normal X properties (e.g., fingerprints), the MD p-value is wildly inaccurate.

Structural Similar Compounds

Name	Tiaramide .HCl (Free base form)	Pirenzepine	N-(3-Piperidyl)3;4;5-trimethoxybenzamide
Structure	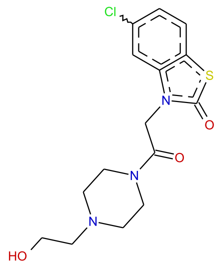	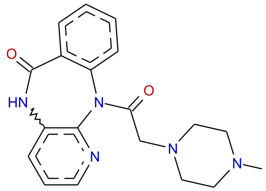	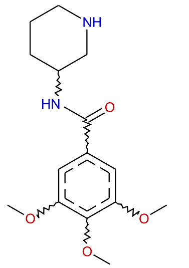
Actual Endpoint	Toxic	Non-Toxic	Toxic
Predicted Endpoint	Toxic	Non-Toxic	Toxic
Distance	0.556	0.601	0.622
Reference	Arzneimittelforschung 23(4):504-8; 1973	Iyakuhin Kenkyu 11:424-436; 1980	Kiso to Rinsho 18:91-101; 1984

Model Applicability

Unknown features are fingerprint features in the query molecule, but not found or appearing too infrequently in the training set.

1. All properties and OPS components are within expected ranges.

Feature Contribution

Top features for positive contribution				
**Fingerprint**	**Bit/Smiles**	**Feature Structure**	**Score**	**Toxic in training set**
SCFP_6	1100167450	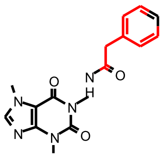 [*]C(=[*])C[c]1:[cH]:[cH]:[*]:[cH]:[cH]:1	0.441	3 out of 3

SCFP_6	-394704229	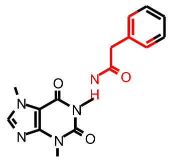 [*]NC(=O)C[c](:[cH]:[*]):[cH]:[*]	0.381	2 out of 2
SCFP_6	1394899299	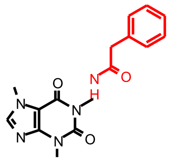 [*]NC(=O)C[c]1:[cH]:[cH]:[cH]:[cH]:[cH]:1	0.381	2 out of 2
**Top Features for negative contribution**				
**Fingerprint**	**Bit/Smiles**	**Feature Structure**	**Score**	**Toxic in training set**
SCFP_6	136358998	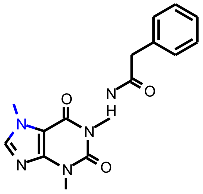 [*]:n(:[*])C	-0.55	2 out of 8
SCFP_6	399659969	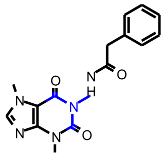 [*]CN(C(=[*])[*])C(=[*])[*]	-0.526	3 out of 11
SCFP_6	-1379591900	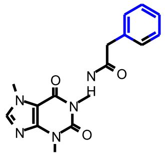 [*][c]1:[*]:[cH]:[cH]:[cH]:[cH]:1	-0.282	33 out of 84

Erlotinib

TOPKAT_Developmental_Toxicity_Potential

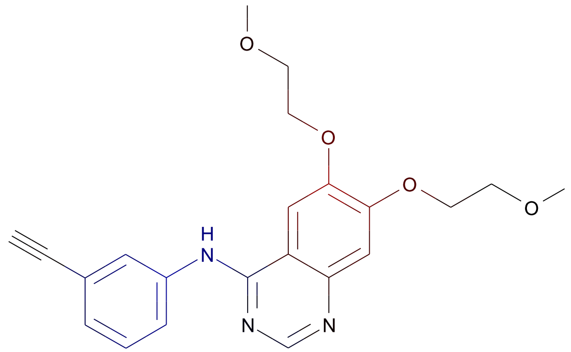

$C_{22}H_{23}N_3O_4$
Molecular Weight: 393.43572
ALogP: 4.309
Rotatable Bonds: 10
Acceptors: 7
Donors: 1

Model Prediction

Prediction: Non-Toxic
Probability: 0.504
Enrichment: 0.959
Bayesian Score: -1.17
Mahalanobis Distance: 8.76
Mahalanobis Distance p-value: 0.258

Prediction: Positive if the Bayesian score is above the estimated best cutoff value from minimizing the false positive and false negative rate.
Probability: The esimated probability that the sample is in the positive category. This assumes that the Bayesian score follows a normal distribution and is different from the prediction using a cutoff.
Enrichment: An estimate of enrichment, that is, the increased likelihood (versus random) of this sample being in the category.
Bayesian Score: The standard Laplacian-modified Bayesian score.
Mahalanobis Distance: The Mahalanobis distance (MD) is the distance to the center of the training data. The larger the MD, the less trustworthy the prediction.
Mahalanobis Distance p-value: The p-value gives the fraction of training data with an MD greater than or equal to the one for the given sample, assuming normally distributed data. The smaller the p-value, the less trustworthy the prediciton. For highly non-normal X properties (e.g., fingerprints), the MD p-value is wildly inaccurate.

Structural Similar Compounds

Name	Nicardipine	Suxibuzone	Etofenamate
Structure	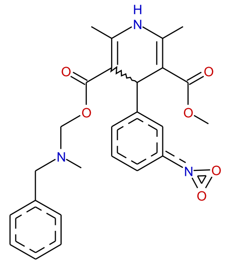	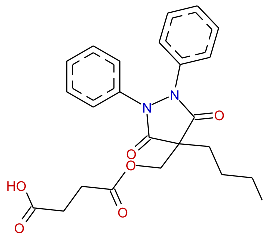	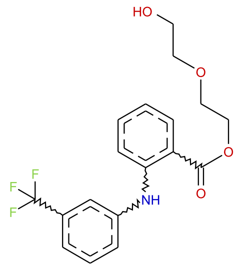
Actual Endpoint	Non-Toxic	Toxic	Non-Toxic
Predicted Endpoint	Non-Toxic	Toxic	Non-Toxic
Distance	0.608	0.620	0.635
Reference	Kiso to Rinsho 13:1149-1159; 1979	Oyo Yakuri 20:377-386; 1980	Iyakuhin Kenkyu 13(4):896-909; 1982

Model Applicability

Unknown features are fingerprint features in the query molecule, but not found or appearing too infrequently in the training set.

1. All properties and OPS components are within expected ranges.

Feature Contribution

Top features for positive contribution

Fingerprint	Bit/Smiles	Feature Structure	Score	Toxic in training set
SCFP_6	123285475	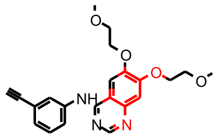 [*]O[c]1:[cH]:[c](:n:[*]):[c](:[*]):[*]:[c]:1[*]	0.478	4 out of 4

SCFP_6	-1814968949	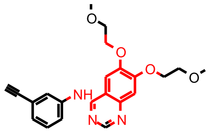 [*]CO[c]1:[cH]:[c]2:[c](N[*]):n:[*]:n:[c]:2:[cH]:[c]:1O[*]	0.381	2 out of 2
SCFP_6	446954673	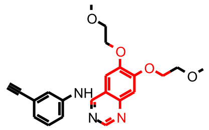 [*]CO[c]1:[cH]:[c]2:n:[cH]:[*]:[c]([*]):[c]:2:[cH]:[c]:1O[*]	0.381	2 out of 2

Top Features for negative contribution

Fingerprint	Bit/Smiles	Feature Structure	Score	Toxic in training set
SCFP_6	2142015375	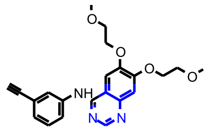 [*]:[cH]:[c]1:n:[cH]:n:[*]:[c]:1:[*]	-0.718	0 out of 2
SCFP_6	-2020651081	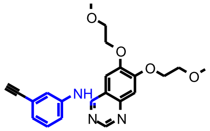 [*][c]1:[cH]:[cH]:[cH]:[c](N[c](:[*]):[*]):[cH]:1	-0.718	0 out of 2
SCFP_6	-300914917	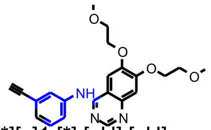 [*][c]1:[*]:[cH]:[cH]:[c](N[c](:[*]):[*]):[cH]:1	-0.718	0 out of 2

Compound 5

TOPKAT_Mouse_Female_FDA_None_vs_Carcinogen

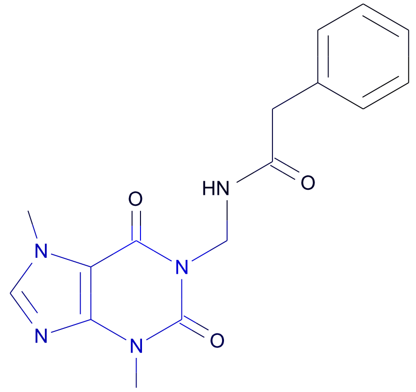

$C_{16}H_{17}N_5O_3$
Molecular Weight: 327.33788
ALogP: 0.773
Rotatable Bonds: 4
Acceptors: 4
Donors: 1

Structural Similar Compounds

Name	Pentoxifylline	Scopolamine	Fluconazole
Structure	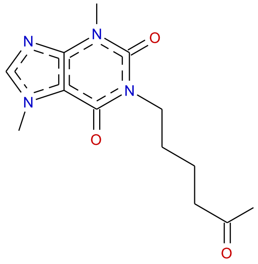	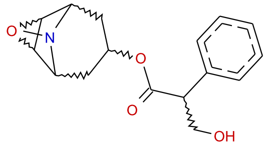	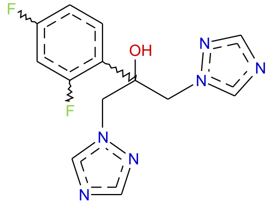
Actual Endpoint	Non-Carcinogen	Non-Carcinogen	Non-Carcinogen
Predicted Endpoint	Non-Carcinogen	Non-Carcinogen	Non-Carcinogen
Distance	0.458	0.557	0.576
Reference	US FDA (Centre for Drug Eval.& Res./Off. Testing & Res.) Sept. 1997	US FDA (Centre for Drug Eval.& Res./Off. Testing & Res.) Sept. 1997	US FDA (Centre for Drug Eval.& Res./Off. Testing & Res.) Sept. 1997

Model Prediction

Prediction: Non-Carcinogen
Probability: 0.211
Enrichment: 0.657
Bayesian Score: -8.39
Mahalanobis Distance: 12.5
Mahalanobis Distance p-value: 0.00234

Prediction: Positive if the Bayesian score is above the estimated best cutoff value from minimizing the false positive and false negative rate.
Probability: The esimated probability that the sample is in the positive category. This assumes that the Bayesian score follows a normal distribution and is different from the prediction using a cutoff.
Enrichment: An estimate of enrichment, that is, the increased likelihood (versus random) of this sample being in the category.
Bayesian Score: The standard Laplacian-modified Bayesian score.
Mahalanobis Distance: The Mahalanobis distance (MD) is the distance to the center of the training data. The larger the MD, the less trustworthy the prediction.
Mahalanobis Distance p-value: The p-value gives the fraction of training data with an MD greater than or equal to the one for the given sample, assuming normally distributed data. The smaller the p-value, the less trustworthy the prediciton. For highly non-normal X properties (e.g., fingerprints), the MD p-value is wildly inaccurate.

Model Applicability

Unknown features are fingerprint features in the query molecule, but not found or appearing too infrequently in the training set.

1. All properties and OPS components are within expected ranges.
2. Unknown ECFP_2 feature: -39630146: [*]NCN([*])[*]

Feature Contribution

Top features for positive contribution

Fingerprint	Bit/Smiles	Feature Structure	Score	Carcinogen in training set
ECFP_6	-407983022	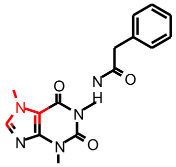 [*][c]1:[*]:[*]:[cH]:n:1C	0.442	2 out of 3

ECFP_6	2106656448	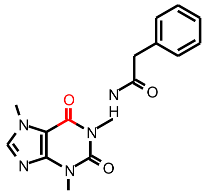 [*]C(=O)[*]	0.254	31 out of 77
ECFP_6	777558239	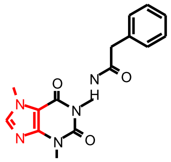 [*][c]1:n:[cH]:n(C):[c]:1[*]	0.212	1 out of 2

Top Features for negative contribution

Fingerprint	Bit/Smiles	Feature Structure	Score	Carcinogen in training set
ECFP_6	-661097313	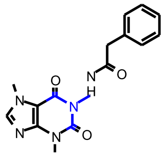 [*]CN(C(=[*])[*])C(=[*])[*]	-1.55	0 out of 12
ECFP_6	1731843802	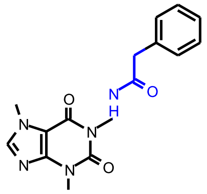 [*]CC(=O)N[*]	-0.657	0 out of 3
ECFP_6	-1677427181	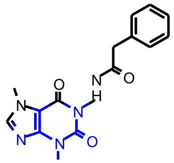 [*]N1[*][c]2:[*]:[*]: n:[c]:2N(C)C1=O	-0.482	0 out of 2

Erlotinib

TOPKAT_Mouse_Female_FDA_None_vs_Carcinogen

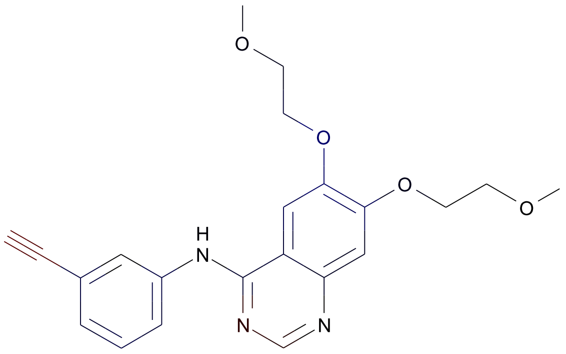

$C_{22}H_{23}N_3O_4$

Molecular Weight: 393.43572

ALogP: 4.309

Rotatable Bonds: 10

Acceptors: 7

Donors: 1

Model Prediction

Prediction: Non-Carcinogen

Probability: 0.235

Enrichment: 0.733

Bayesian Score: -1.68

Mahalanobis Distance: 15.3

Mahalanobis Distance p-value: 7.99e-008

Prediction: Positive if the Bayesian score is above the estimated best cutoff value from minimizing the false positive and false negative rate.
Probability: The esimated probability that the sample is in the positive category. This assumes that the Bayesian score follows a normal distribution and is different from the prediction using a cutoff.
Enrichment: An estimate of enrichment, that is, the increased likelihood (versus random) of this sample being in the category.
Bayesian Score: The standard Laplacian-modified Bayesian score.
Mahalanobis Distance: The Mahalanobis distance (MD) is the distance to the center of the training data. The larger the MD, the less trustworthy the prediction.
Mahalanobis Distance p-value: The p-value gives the fraction of training data with an MD greater than or equal to the one for the given sample, assuming normally distributed data. The smaller the p-value, the less trustworthy the prediciton. For highly non-normal X properties (e.g., fingerprints), the MD p-value is wildly inaccurate.

Structural Similar Compounds

Name	Mycophenolate	Nicardipine	Nimodipine
Structure	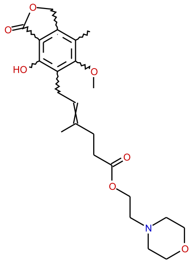	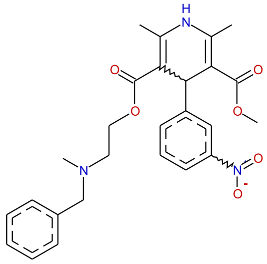	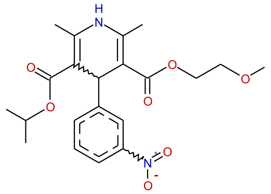
Actual Endpoint	Non-Carcinogen	Non-Carcinogen	Non-Carcinogen
Predicted Endpoint	Non-Carcinogen	Non-Carcinogen	Non-Carcinogen
Distance	0.632	0.664	0.675
Reference	US FDA (Centre for Drug Eval.& Res./Off. Testing & Res.) Sept. 1997	US FDA (Centre for Drug Eval.& Res./Off. Testing & Res.) Sept. 1997	US FDA (Centre for Drug Eval.& Res./Off. Testing & Res.) Sept. 1997

Model Applicability

Unknown features are fingerprint features in the query molecule, but not found or appearing too infrequently in the training set.

1. All properties and OPS components are within expected ranges.
2. Unknown ECFP_2 feature: -182178874: [*]#C[c](:c:[*]):c:[*]
3. Unknown ECFP_2 feature: 1139738044: [*]:[c](:[*])C#C

Feature Contribution

Top features for positive contribution

Fingerprint	Bit/Smiles	Feature Structure	Score	Carcinogen in training set
ECFP_6	-1939823063	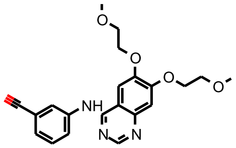 [*]#C	0.866	8 out of 9

ECFP_6	-1545539812	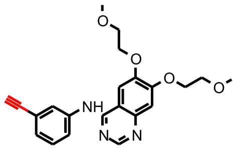<br>[*]C#C	0.866	8 out of 9
ECFP_6	-1114776580	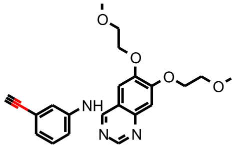<br>[*]C#[*]	0.755	11 out of 15

Top Features for negative contribution

**Fingerprint**	**Bit/Smiles**	**Feature Structure**	**Score**	**Carcinogen in training set**
ECFP_6	2007300961	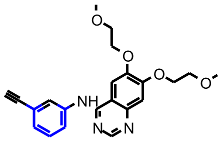<br>[*][c]1:[*]:[c]([*]):<br>[cH]:[cH]:[cH]:1	-0.652	5 out of 34
ECFP_6	-2063202154	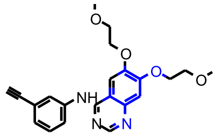<br>[*]O[c]1:[cH]:[c](:n:<br>[*]):[c](:[*]):[*]:[<br>c]:1[*]	-0.482	0 out of 2
ECFP_6	-1242906247	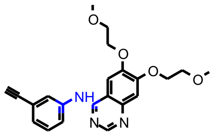<br>[*]:[c](:[*])N[c](:[*<br>]):[*]	-0.482	0 out of 2

Compound 5

TOPKAT_Mouse_Male_FDA_None_vs_Carcinogen

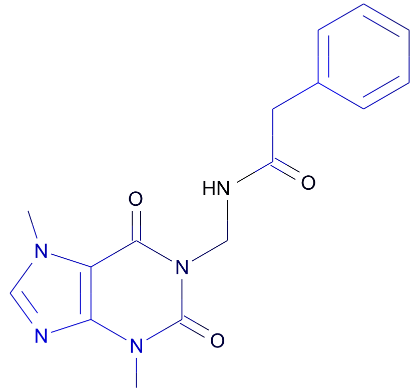

$C_{16}H_{17}N_5O_3$
Molecular Weight: 327.33788
ALogP: 0.773
Rotatable Bonds: 4
Acceptors: 4
Donors: 1

Structural Similar Compounds

Name	Pentoxifylline	Fluconazole	Scopolamine
Structure	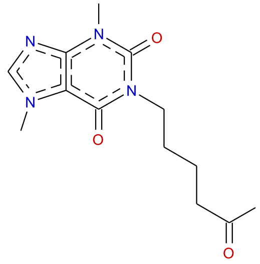	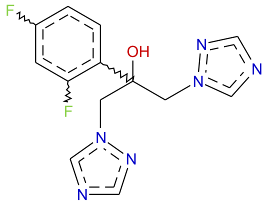	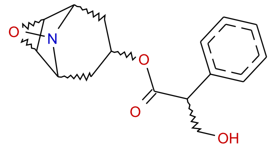
Actual Endpoint	Non-Carcinogen	Non-Carcinogen	Non-Carcinogen
Predicted Endpoint	Non-Carcinogen	Non-Carcinogen	Non-Carcinogen
Distance	0.472	0.559	0.561
Reference	US FDA (Centre for Drug Eval.& Res./Off. Testing & Res.) Sept. 1997	US FDA (Centre for Drug Eval.& Res./Off. Testing & Res.) Sept. 1997	US FDA (Centre for Drug Eval.& Res./Off. Testing & Res.) Sept. 1997

Model Prediction

Prediction: Non-Carcinogen
Probability: 0.135
Enrichment: 0.459
Bayesian Score: -10.7
Mahalanobis Distance: 11.8
Mahalanobis Distance p-value: 0.0103

Prediction: Positive if the Bayesian score is above the estimated best cutoff value from minimizing the false positive and false negative rate.
Probability: The esimated probability that the sample is in the positive category. This assumes that the Bayesian score follows a normal distribution and is different from the prediction using a cutoff.
Enrichment: An estimate of enrichment, that is, the increased likelihood (versus random) of this sample being in the category.
Bayesian Score: The standard Laplacian-modified Bayesian score.
Mahalanobis Distance: The Mahalanobis distance (MD) is the distance to the center of the training data. The larger the MD, the less trustworthy the prediction.
Mahalanobis Distance p-value: The p-value gives the fraction of training data with an MD greater than or equal to the one for the given sample, assuming normally distributed data. The smaller the p-value, the less trustworthy the prediciton. For highly non-normal X properties (e.g., fingerprints), the MD p-value is wildly inaccurate.

Model Applicability

Unknown features are fingerprint features in the query molecule, but not found or appearing too infrequently in the training set.

1. All properties and OPS components are within expected ranges.

Feature Contribution

Top features for positive contribution

Fingerprint	Bit/Smiles	Feature Structure	Score	Carcinogen in training set
FCFP_6	470101049	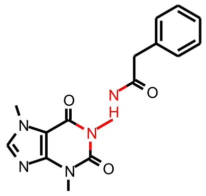 [*]NCN([*])[*]	0.46	1 out of 1

FCFP_6	566058135	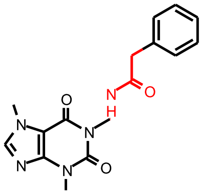 [*]CC(=O)N[*]	0.447	17 out of 40
FCFP_6	-1539132615	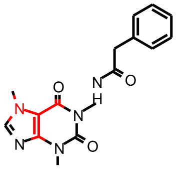 [*]n1:[*]:[*]:[c]([*]):[c]:1C(=[*])[*]	0.328	19 out of 51
**Top Features for negative contribution**				
**Fingerprint**	**Bit/Smiles**	**Feature Structure**	**Score**	**Carcinogen in training set**
FCFP_6	1981711554	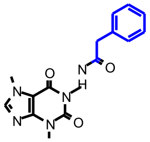 [*]C(=[*])C[c]1:[cH]: [cH]:[cH]:[cH]:[cH]: 1	-1.42	0 out of 12
FCFP_6	-497728148	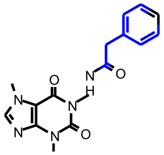 [*]C(=[*])C[c]1:[cH]: [cH]:[*]:[cH]:[cH]:1	-0.96	2 out of 26
FCFP_6	-124685461	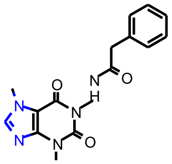 [*]n1:[*]:[*]:n:[cH]: 1	-0.731	1 out of 12

Erlotinib

TOPKAT_Mouse_Male_FDA_None_vs_Carcinogen

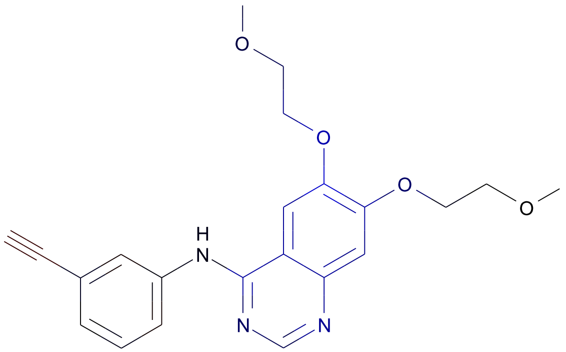

$C_{22}H_{23}N_3O_4$

Molecular Weight: 393.43572

ALogP: 4.309

Rotatable Bonds: 10

Acceptors: 7

Donors: 1

Structural Similar Compounds

Name	Mycophenolate	Nicardipine	Nimodipine
Structure	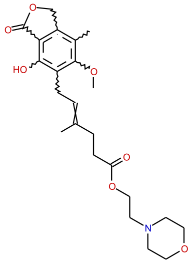	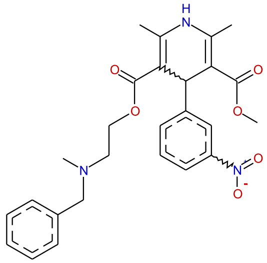	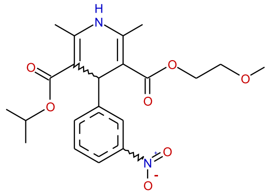
Actual Endpoint	Non-Carcinogen	Non-Carcinogen	Non-Carcinogen
Predicted Endpoint	Non-Carcinogen	Non-Carcinogen	Non-Carcinogen
Distance	0.615	0.655	0.668
Reference	US FDA (Centre for Drug Eval.& Res./Off. Testing & Res.) Sept. 1997	US FDA (Centre for Drug Eval.& Res./Off. Testing & Res.) Sept. 1997	US FDA (Centre for Drug Eval.& Res./Off. Testing & Res.) Sept. 1997

Model Prediction

Prediction: Non-Carcinogen

Probability: 0.181

Enrichment: 0.614

Bayesian Score: -6.29

Mahalanobis Distance: 16

Mahalanobis Distance p-value: 1.07e-009

Prediction: Positive if the Bayesian score is above the estimated best cutoff value from minimizing the false positive and false negative rate.
Probability: The esimated probability that the sample is in the positive category. This assumes that the Bayesian score follows a normal distribution and is different from the prediction using a cutoff.
Enrichment: An estimate of enrichment, that is, the increased likelihood (versus random) of this sample being in the category.
Bayesian Score: The standard Laplacian-modified Bayesian score.
Mahalanobis Distance: The Mahalanobis distance (MD) is the distance to the center of the training data. The larger the MD, the less trustworthy the prediction.
Mahalanobis Distance p-value: The p-value gives the fraction of training data with an MD greater than or equal to the one for the given sample, assuming normally distributed data. The smaller the p-value, the less trustworthy the prediciton. For highly non-normal X properties (e.g., fingerprints), the MD p-value is wildly inaccurate.

Model Applicability

Unknown features are fingerprint features in the query molecule, but not found or appearing too infrequently in the training set.

1. All properties and OPS components are within expected ranges.
2. Unknown FCFP_2 feature: 902193919: [*]:[c](:[*])C#C

Feature Contribution

Top features for positive contribution

Fingerprint	Bit/Smiles	Feature Structure	Score	Carcinogen in training set
FCFP_6	131784192	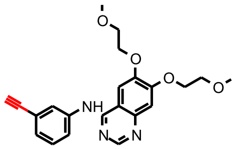 [*]C#C	0.983	8 out of 9

FCFP_6	-773983804	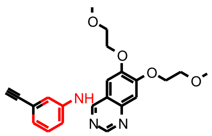<br>[*]N[c]1:[cH]:[*]:[cH]:[cH]:[cH]:1	0.409	10 out of 24
FCFP_6	-771557733	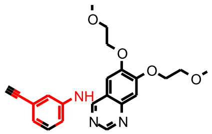<br>[*]N[c]1:[cH]:[*]:[cH]:[c](:[cH]:1)C#[*]	0.38	2 out of 4
**Top Features for negative contribution**				
**Fingerprint**	**Bit/Smiles**	**Feature Structure**	**Score**	**Carcinogen in training set**
FCFP_6	-124685461	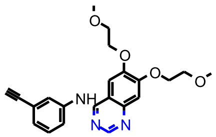<br>[*]n1:[*]:[*]:n:[cH]:1	-0.731	1 out of 12
FCFP_6	1674955425	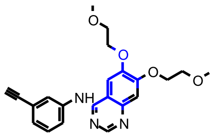<br>[*]O[c]1:[cH]:[c](:[c]([*]):[*]):[c](:[*]):[*]:[c]:1[*]	-0.719	0 out of 4
FCFP_6	1293778554	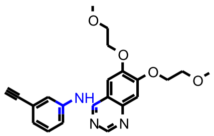<br>[*]:[c](:[*])N[c](:[*]):[*]	-0.719	0 out of 4

Compound 5

TOPKAT_Ocular_Irritancy_Mild_vs_Moderate_Severe

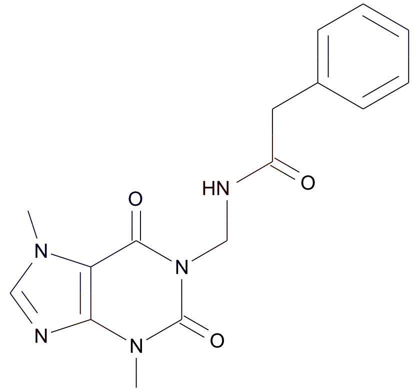

$C_{16}H_{17}N_5O_3$
Molecular Weight: 327.33788
ALogP: 0.773
Rotatable Bonds: 4
Acceptors: 4
Donors: 1

Model Prediction

Prediction: Mild

Probability: 0.811

Enrichment: 1.18

Bayesian Score: -0.533

Mahalanobis Distance: 10.6

Mahalanobis Distance p-value: 0.018

Prediction: Positive if the Bayesian score is above the estimated best cutoff value from minimizing the false positive and false negative rate.
Probability: The esimated probability that the sample is in the positive category. This assumes that the Bayesian score follows a normal distribution and is different from the prediction using a cutoff.
Enrichment: An estimate of enrichment, that is, the increased likelihood (versus random) of this sample being in the category.
Bayesian Score: The standard Laplacian-modified Bayesian score.
Mahalanobis Distance: The Mahalanobis distance (MD) is the distance to the center of the training data. The larger the MD, the less trustworthy the prediction.
Mahalanobis Distance p-value: The p-value gives the fraction of training data with an MD greater than or equal to the one for the given sample, assuming normally distributed data. The smaller the p-value, the less trustworthy the prediciton. For highly non-normal X properties (e.g., fingerprints), the MD p-value is wildly inaccurate.

Structural Similar Compounds

Name	2;5-DICHLORO-4(3'-METHYL-5' PYRAZOLON-1'-YL)BENZENE SULFONIC ACID	0-ANISIDINE;ACETOACETYL	3(2H)-Pyridazinone; 5-amino-4-chloro-2-phenyl-
Structure	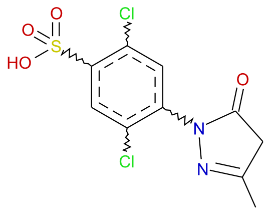	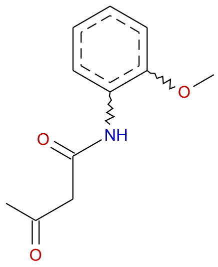	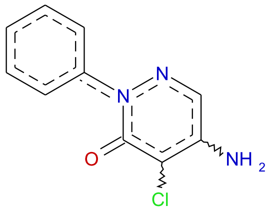
Actual Endpoint	Mild	Mild	Mild
Predicted Endpoint	Mild	Mild	Mild
Distance	0.665	0.683	0.686
Reference	28ZPAK-;186;72	28ZPAK-;116;72	Prehled Prumyslove Toxikologie; Organicke Latky; Marhold; J. pp 868;86

Model Applicability

Unknown features are fingerprint features in the query molecule, but not found or appearing too infrequently in the training set.

1. All properties and OPS components are within expected ranges.
2. Unknown FCFP_2 feature: -124685461: [*]n1:[*]:[*]:n:[cH]:1
3. Unknown FCFP_2 feature: 136150461: [*]:n(:[*])C

Feature Contribution

Top features for positive contribution				
**Fingerprint**	**Bit/Smiles**	**Feature Structure**	**Score**	**Moderate_Severe in training set**

FCFP_10	-497728148	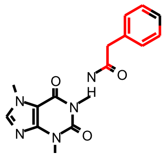<br>[*]C(=[*])C[c]1:[cH]:<br>[cH]:[*]:[cH]:[cH]:1	0.356	24 out of 25
FCFP_10	-1410049896	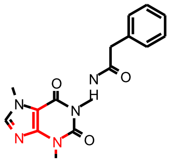<br>[*]N([*])[c]1:n:[*]:[<br>*]:[c]:1[*]	0.256	2 out of 2
FCFP_10	470101049	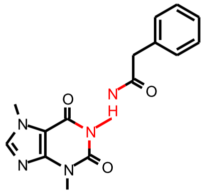<br>[*]NCN([*])[*]	0.256	2 out of 2
**Top Features for negative contribution**				
**Fingerprint**	**Bit/Smiles**	**Feature Structure**	**Score**	**Moderate_Severe in training set**
FCFP_10	-306856457	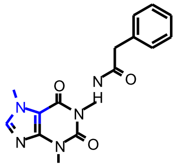<br>[*][c]1:[*]:[*]:[*]:[cH]:<br>n:1C	-0.842	0 out of 2
FCFP_10	-1549163031	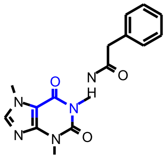<br>[*]N([*])C(=O)[c](:[*<br>]):[*]	-0.657	5 out of 16

FCFP_10	136627117	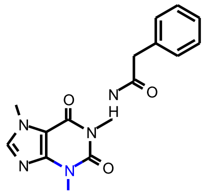 [*]N([*])C	-0.316	46 out of 96

Erlotinib

TOPKAT_Ocular_Irritancy_Mild_vs_Moderate_Severe

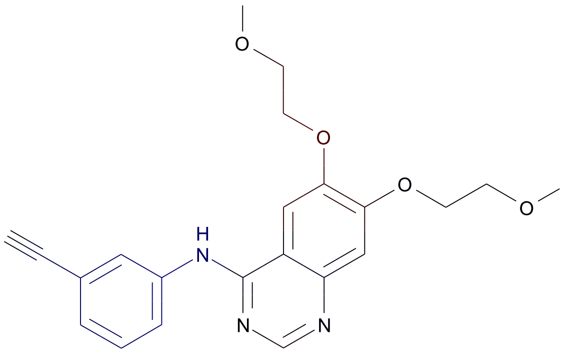

$C_{22}H_{23}N_3O_4$

Molecular Weight: 393.43572

ALogP: 4.309

Rotatable Bonds: 10

Acceptors: 7

Donors: 1

Model Prediction

Prediction: Mild

Probability: 0.766

Enrichment: 1.11

Bayesian Score: -2.1

Mahalanobis Distance: 10.4

Mahalanobis Distance p-value: 0.0368

Prediction: Positive if the Bayesian score is above the estimated best cutoff value from minimizing the false positive and false negative rate.
Probability: The esimated probability that the sample is in the positive category. This assumes that the Bayesian score follows a normal distribution and is different from the prediction using a cutoff.
Enrichment: An estimate of enrichment, that is, the increased likelihood (versus random) of this sample being in the category.
Bayesian Score: The standard Laplacian-modified Bayesian score.
Mahalanobis Distance: The Mahalanobis distance (MD) is the distance to the center of the training data. The larger the MD, the less trustworthy the prediction.
Mahalanobis Distance p-value: The p-value gives the fraction of training data with an MD greater than or equal to the one for the given sample, assuming normally distributed data. The smaller the p-value, the less trustworthy the prediciton. For highly non-normal X properties (e.g., fingerprints), the MD p-value is wildly inaccurate.

Structural Similar Compounds

Name	Benzoic acid; p-(N-butyl-2-(butylamino)acetamido)-; butyl ester;	COLCHICINE	Benzoic acid; p-(N-butyl-2-(piperidino)acetamido)-; butyl ester;
Structure	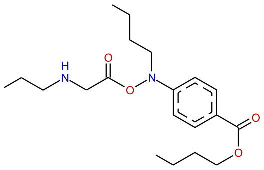	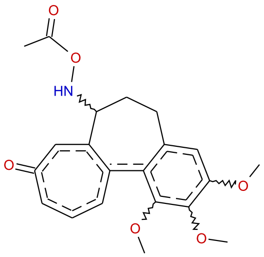	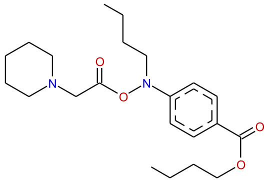
Actual Endpoint	Moderate_Severe	Moderate_Severe	Moderate_Severe
Predicted Endpoint	Moderate_Severe	Moderate_Severe	Moderate_Severe
Distance	0.648	0.707	0.750
Reference	Arzneimittel-Forschung 8;609;58	AJOPAA 31;837;48	Arzneimittel-Forschung 8;609;58

Model Applicability

Unknown features are fingerprint features in the query molecule, but not found or appearing too infrequently in the training set.

1. All properties and OPS components are within expected ranges.
2. Unknown FCFP_2 feature: -1151884458: [*]N[c](:n:[*]):[c](:[*]):[*]
3. Unknown FCFP_2 feature: -124685461: [*]n1:[*]:[*]:n:[cH]:1
4. Unknown FCFP_2 feature: 902193919: [*]:[c](:[*])C#C

Feature Contribution

Top features for positive contribution				
Fingerprint	Bit/Smiles	Feature Structure	Score	Moderate_Severe in training set
FCFP_10	365650923	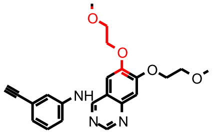 [*]OCCO[c](:[*]):[*]	0.386	17 out of 17

FCFP_10	-1059904848	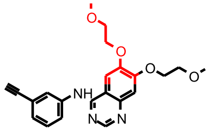 [*][c](:[*]):[c](OCCO C):[cH]:[*]	0.386	17 out of 17
FCFP_10	-1716224640	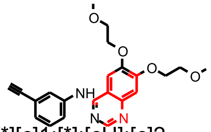 [*][c]1:[*]:[cH]:[c]2 :[c]([*]):[*]:[cH]:n :[c]:2:[cH]:1	0.294	3 out of 3
**Top Features for negative contribution**				
**Fingerprint**	**Bit/Smiles**	**Feature Structure**	**Score**	**Moderate_Severe in training set**
FCFP_10	-1699003333	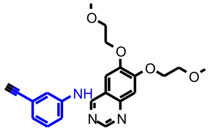 [*]N[c]1:[cH]:[cH]:[c H]:[c](:[cH]:1)C#[*]	-1.09	2 out of 12
FCFP_10	1679603620	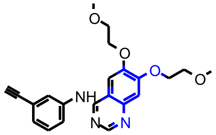 [*]O[c]1:[cH]:[c](:n: [*]):[c](:[*]):[*]:[c]:1[*]	-0.507	0 out of 1
FCFP_10	341504799	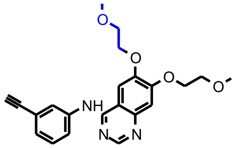 [*]CCOC	-0.425	7 out of 17

Compound 5

TOPKAT_Ocular_Irritancy_None_vs_Irritant

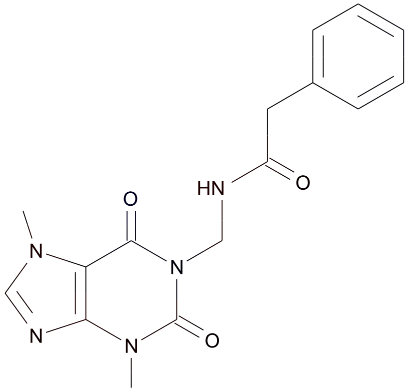

$C_{16}H_{17}N_5O_3$
Molecular Weight: 327.33788
ALogP: 0.773
Rotatable Bonds: 4
Acceptors: 4
Donors: 1

Structural Similar Compounds

Name	2;5-DICHLORO-4(3'-METHYL-5' PYRAZOLON-1'-YL)BENZENE SULFONIC ACID	Ammonium; ((N-anthraquinon-2-yl)aminomethylene)dimethyl-; chloride	0-ANISIDINE;ACETOACETYL
Structure	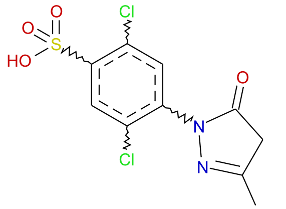	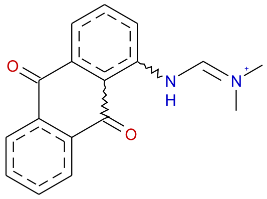	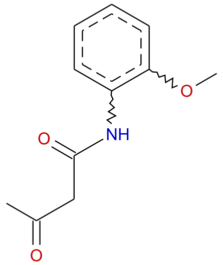
Actual Endpoint	Irritant	Irritant	Irritant
Predicted Endpoint	Irritant	Irritant	Irritant
Distance	0.657	0.674	0.674
Reference	28ZPAK-;186;72	Prehled Prumyslove Toxikologie; Organicke Latky; Marhold; J. -;732;86	28ZPAK-;116;72

Model Prediction

Prediction: Irritant
Probability: 1
Enrichment: 1.18
Bayesian Score: 1.1
Mahalanobis Distance: 6.16
Mahalanobis Distance p-value: 1

Prediction: Positive if the Bayesian score is above the estimated best cutoff value from minimizing the false positive and false negative rate.
Probability: The esimated probability that the sample is in the positive category. This assumes that the Bayesian score follows a normal distribution and is different from the prediction using a cutoff.
Enrichment: An estimate of enrichment, that is, the increased likelihood (versus random) of this sample being in the category.
Bayesian Score: The standard Laplacian-modified Bayesian score.
Mahalanobis Distance: The Mahalanobis distance (MD) is the distance to the center of the training data. The larger the MD, the less trustworthy the prediction.
Mahalanobis Distance p-value: The p-value gives the fraction of training data with an MD greater than or equal to the one for the given sample, assuming normally distributed data. The smaller the p-value, the less trustworthy the prediciton. For highly non-normal X properties (e.g., fingerprints), the MD p-value is wildly inaccurate.

Model Applicability

Unknown features are fingerprint features in the query molecule, but not found or appearing too infrequently in the training set.

1. All properties and OPS components are within expected ranges.
2. Unknown FCFP_2 feature: -124685461: [*]n1:[*]:[*]:n:[cH]:1
3. Unknown FCFP_2 feature: 136150461: [*]:n(:[*])C

Feature Contribution

Top features for positive contribution

Fingerprint	Bit/Smiles	Feature Structure	Score	Irritant in training set
FCFP_12	1747237384	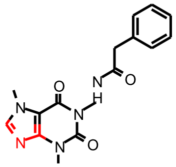 [*][c]1:[*]:[*]:[cH]:n:1	0.208	44 out of 44

FCFP_12	-1539132615	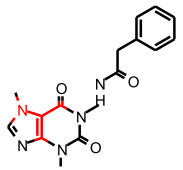 [*]n1:[*]:[*]:[c]([*]):[c]:1C(=[*])[*]	0.197	13 out of 13
FCFP_12	17	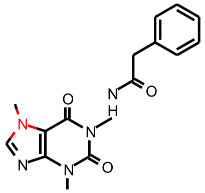 [*]n(:[*]):[*]	0.189	48 out of 49

Top Features for negative contribution

Fingerprint	Bit/Smiles	Feature Structure	Score	Irritant in training set
FCFP_12	-1549163031	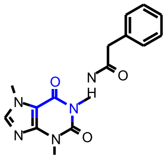 [*]N([*])C(=O)[c](:[*]):[*]	-0.623	16 out of 38
FCFP_12	1981711554	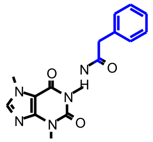 [*]C(=[*])C[c]1:[cH]: [cH]:[cH]:[cH]:[cH]: 1	-0.103	5 out of 7
FCFP_12	-1698724694	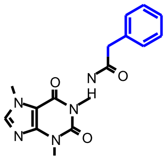 [*]C[c]1:[cH]:[cH]:[c H]:[cH]:[cH]:1	-0.0964	107 out of 146

Erlotinib

TOPKAT_Ocular_Irritancy_None_vs_Irritant

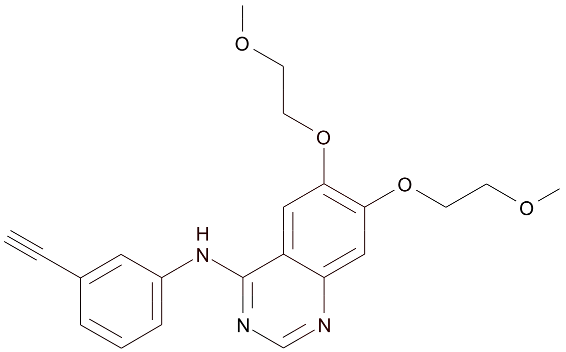

$C_{22}H_{23}N_3O_4$

Molecular Weight: 393.43572

ALogP: 4.309

Rotatable Bonds: 10

Acceptors: 7

Donors: 1

Structural Similar Compounds

Name	Benzoic acid; p-(N-butyl-2-(butylamino)acetamido)-; butyl ester;	COLCHICINE	Cinchoninamide; 2-butoxy-N-(2-(diethylamino)ethyl)-; monohydrochloride
Structure	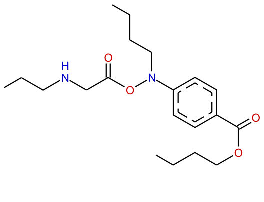	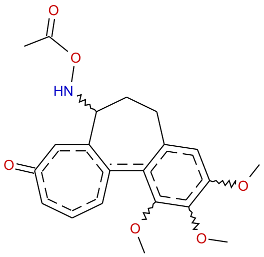	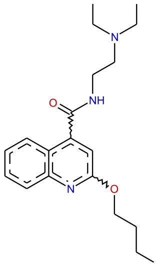
Actual Endpoint	Irritant	Irritant	Irritant
Predicted Endpoint	Non-Irritant	Irritant	Irritant
Distance	0.637	0.682	0.747
Reference	Arzneimittel-Forschung 8;609;58	AJOPAA 31;837;48	Arzneimittel-Forschung 8;181;58

Model Prediction

Prediction: Irritant

Probability: 1

Enrichment: 1.18

Bayesian Score: 2.13

Mahalanobis Distance: 9.75

Mahalanobis Distance p-value: 0.161

Prediction: Positive if the Bayesian score is above the estimated best cutoff value from minimizing the false positive and false negative rate.
Probability: The esimated probability that the sample is in the positive category. This assumes that the Bayesian score follows a normal distribution and is different from the prediction using a cutoff.
Enrichment: An estimate of enrichment, that is, the increased likelihood (versus random) of this sample being in the category.
Bayesian Score: The standard Laplacian-modified Bayesian score.
Mahalanobis Distance: The Mahalanobis distance (MD) is the distance to the center of the training data. The larger the MD, the less trustworthy the prediction.
Mahalanobis Distance p-value: The p-value gives the fraction of training data with an MD greater than or equal to the one for the given sample, assuming normally distributed data. The smaller the p-value, the less trustworthy the prediciton. For highly non-normal X properties (e.g., fingerprints), the MD p-value is wildly inaccurate.

Model Applicability

Unknown features are fingerprint features in the query molecule, but not found or appearing too infrequently in the training set.

1. All properties and OPS components are within expected ranges.
2. Unknown FCFP_2 feature: -1151884458: [*]N[c](:n:[*]):[c](:[*]):[*]
3. Unknown FCFP_2 feature: -124685461: [*]n1:[*]:[*]:n:[cH]:1
4. Unknown FCFP_2 feature: 902193919: [*]:[c](:[*])C#C

Feature Contribution

Top features for positive contribution				
Fingerprint	Bit/Smiles	Feature Structure	Score	Irritant in training set
FCFP_12	1747237384	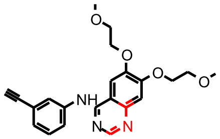 [*][c]1:[*]:[*]:[cH]:n:1	0.208	44 out of 44

FCFP_12	178336375	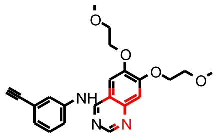 [*]:[cH]:[c](:n:[*]):[c](:[*]):[*]	0.202	19 out of 19
FCFP_12	17	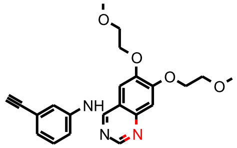 [*]n(:[*]):[*]	0.189	48 out of 49

Top Features for negative contribution

Fingerprint	Bit/Smiles	Feature Structure	Score	Irritant in training set
FCFP_12	-453677277	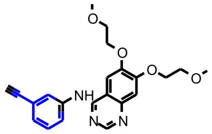 [*]C[c]1:[cH]:[*]:[cH]:[cH]:[cH]:1	0	264 out of 323
FCFP_12	991735244	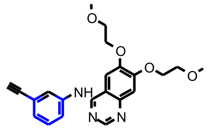 [*][c]1:[*]:[cH]:[cH]:[cH]:[cH]:1	0	237 out of 291
FCFP_12	-773983804	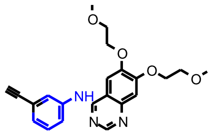 [*]N[c]1:[cH]:[*]:[cH]:[cH]:[cH]:1	0	102 out of 121

Compound 5

TOPKAT_Rat_Female_FDA_None_vs_Carcinogen

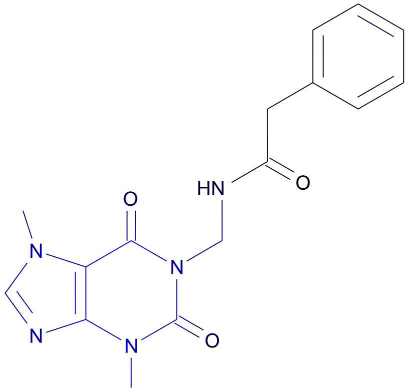

$C_{16}H_{17}N_5O_3$

Molecular Weight: 327.33788

ALogP: 0.773

Rotatable Bonds: 4

Acceptors: 4

Donors: 1

Structural Similar Compounds

Name	Pentoxifylline	Scopolamine	Fluconazole
Structure	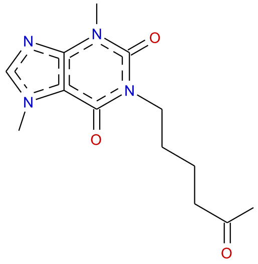	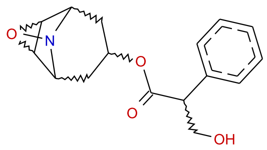	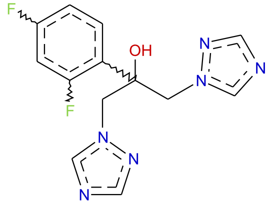
Actual Endpoint	Non-Carcinogen	Non-Carcinogen	Non-Carcinogen
Predicted Endpoint	Non-Carcinogen	Non-Carcinogen	Non-Carcinogen
Distance	0.514	0.576	0.588
Reference	US FDA (Centre for Drug Eval.& Res./Off. Testing & Res.) Sept. 1997	US FDA (Centre for Drug Eval.& Res./Off. Testing & Res.) Sept. 1997	US FDA (Centre for Drug Eval.& Res./Off. Testing & Res.) Sept. 1997

Model Prediction

Prediction: Non-Carcinogen

Probability: 0.199

Enrichment: 0.618

Bayesian Score: -7.3

Mahalanobis Distance: 12.4

Mahalanobis Distance p-value: 0.0011

Prediction: Positive if the Bayesian score is above the estimated best cutoff value from minimizing the false positive and false negative rate.
Probability: The esimated probability that the sample is in the positive category. This assumes that the Bayesian score follows a normal distribution and is different from the prediction using a cutoff.
Enrichment: An estimate of enrichment, that is, the increased likelihood (versus random) of this sample being in the category.
Bayesian Score: The standard Laplacian-modified Bayesian score.
Mahalanobis Distance: The Mahalanobis distance (MD) is the distance to the center of the training data. The larger the MD, the less trustworthy the prediction.
Mahalanobis Distance p-value: The p-value gives the fraction of training data with an MD greater than or equal to the one for the given sample, assuming normally distributed data. The smaller the p-value, the less trustworthy the prediciton. For highly non-normal X properties (e.g., fingerprints), the MD p-value is wildly inaccurate.

Model Applicability

Unknown features are fingerprint features in the query molecule, but not found or appearing too infrequently in the training set.

1. All properties and OPS components are within expected ranges.
2. Unknown ECFP_2 feature: -39630146: [*]NCN([*])[*]

Feature Contribution

Top features for positive contribution				
**Fingerprint**	**Bit/Smiles**	**Feature Structure**	**Score**	**Carcinogen in training set**
ECFP_12	1737023319	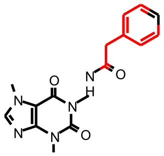 [*]C(=[*])C[c]1:[cH]:[cH]:[*]:[cH]:[cH]:1	0.613	2 out of 2

ECFP_12	-407983022	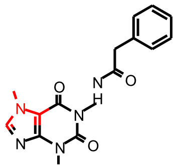 [*][c]1:[*]:[*]:[cH]:n:1C	0.437	2 out of 3
ECFP_12	866450950	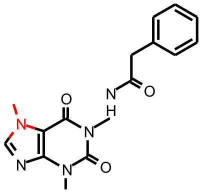 [*]:n(:[*])C	0.33	3 out of 6
**Top Features for negative contribution**				
**Fingerprint**	**Bit/Smiles**	**Feature Structure**	**Score**	**Carcinogen in training set**
ECFP_12	497523368	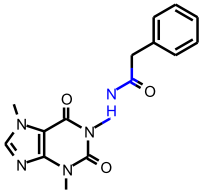 [*]CNC(=[*])[*]	-0.989	1 out of 14
ECFP_12	-281505363	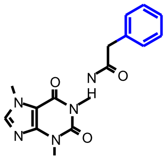 [*][c]1:[cH]:[cH]:[cH]:[cH]:[cH]:1	-0.56	11 out of 64
ECFP_12	1571214559	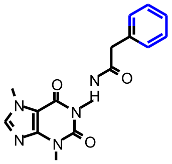 [*]1:[cH]:[cH]:[cH]:[cH]:[cH]:1	-0.56	11 out of 64

Erlotinib

TOPKAT_Rat_Female_FDA_None_vs_Carcinogen

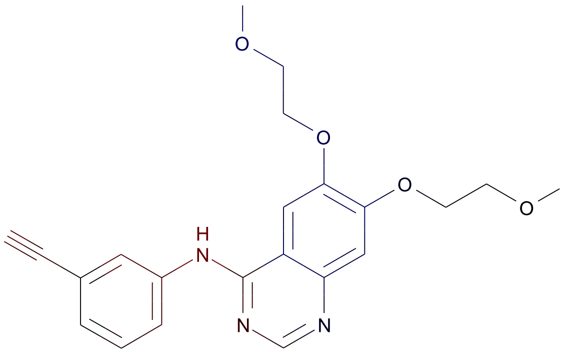

$C_{22}H_{23}N_3O_4$

Molecular Weight: 393.43572

ALogP: 4.309

Rotatable Bonds: 10

Acceptors: 7

Donors: 1

Model Prediction

Prediction: Carcinogen

Probability: 0.285

Enrichment: 0.885

Bayesian Score: -0.692

Mahalanobis Distance: 16.3

Mahalanobis Distance p-value: 1.65e-011

Prediction: Positive if the Bayesian score is above the estimated best cutoff value from minimizing the false positive and false negative rate.
Probability: The esimated probability that the sample is in the positive category. This assumes that the Bayesian score follows a normal distribution and is different from the prediction using a cutoff.
Enrichment: An estimate of enrichment, that is, the increased likelihood (versus random) of this sample being in the category.
Bayesian Score: The standard Laplacian-modified Bayesian score.
Mahalanobis Distance: The Mahalanobis distance (MD) is the distance to the center of the training data. The larger the MD, the less trustworthy the prediction.
Mahalanobis Distance p-value: The p-value gives the fraction of training data with an MD greater than or equal to the one for the given sample, assuming normally distributed data. The smaller the p-value, the less trustworthy the prediciton. For highly non-normal X properties (e.g., fingerprints), the MD p-value is wildly inaccurate.

Structural Similar Compounds

Name	Mycophenolate	Nicardipine	Nimodipine
Structure	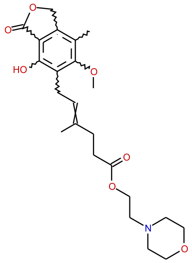	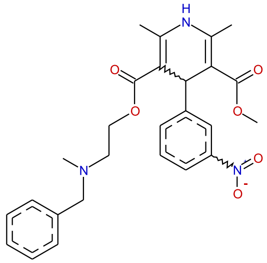	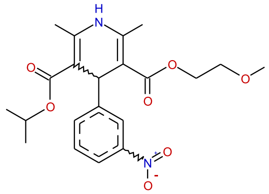
Actual Endpoint	Non-Carcinogen	Carcinogen	Non-Carcinogen
Predicted Endpoint	Non-Carcinogen	Carcinogen	Non-Carcinogen
Distance	0.644	0.660	0.675
Reference	US FDA (Centre for Drug Eval.& Res./Off. Testing & Res.) Sept. 1997	US FDA (Centre for Drug Eval.& Res./Off. Testing & Res.) Sept. 1997	US FDA (Centre for Drug Eval.& Res./Off. Testing & Res.) Sept. 1997

Model Applicability

Unknown features are fingerprint features in the query molecule, but not found or appearing too infrequently in the training set.

1. OPS PC28 out of range. Value: 3.6564. Training min, max, SD, explained variance: -2.8936, 3.5771, 1.028, 0.0111.
2. Unknown ECFP_2 feature: 1139738044: [*]:[c](:[*])C#C

Feature Contribution

Top features for positive contribution

Fingerprint	Bit/Smiles	Feature Structure	Score	Carcinogen in training set
ECFP_12	-1545539812	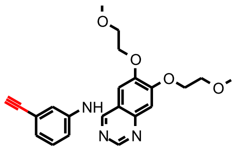 [*]C#C	0.78	8 out of 10

ECFP_12	-1939823063	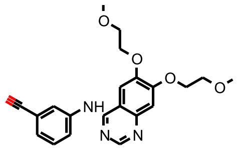 [*]#C	0.78	8 out of 10
ECFP_12	-177077903	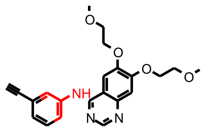 [*]N[c](:[cH]:[*]):[cH]:[*]	0.529	6 out of 10

Top Features for negative contribution

Fingerprint	Bit/Smiles	Feature Structure	Score	Carcinogen in training set
ECFP_12	-2063600634	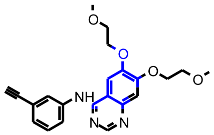 [*]O[c]1:[cH]:[c](:[c]([*]):[*]):[c](:[*]):[*]:[c]:1[*]	-0.661	0 out of 3
ECFP_12	301015111	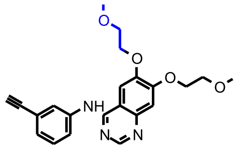 [*]CCOC	-0.661	0 out of 3
ECFP_12	-1253653003	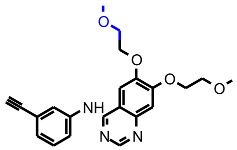 [*]COC	-0.661	0 out of 3

Erlotinib

TOPKAT_Rat_Female_FDA_Single_vs_Multiple

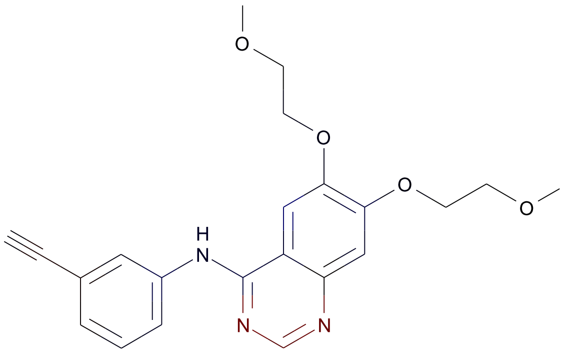

$C_{22}H_{23}N_3O_4$

Molecular Weight: 393.43572

ALogP: 4.309

Rotatable Bonds: 10

Acceptors: 7

Donors: 1

Model Prediction

Prediction: Single-Carcinogen

Probability: 0.423

Enrichment: 1.13

Bayesian Score: -2.63

Mahalanobis Distance: 15.2

Mahalanobis Distance p-value: 2.08e-006

Prediction: Positive if the Bayesian score is above the estimated best cutoff value from minimizing the false positive and false negative rate.
Probability: The esimated probability that the sample is in the positive category. This assumes that the Bayesian score follows a normal distribution and is different from the prediction using a cutoff.
Enrichment: An estimate of enrichment, that is, the increased likelihood (versus random) of this sample being in the category.
Bayesian Score: The standard Laplacian-modified Bayesian score.
Mahalanobis Distance: The Mahalanobis distance (MD) is the distance to the center of the training data. The larger the MD, the less trustworthy the prediction.
Mahalanobis Distance p-value: The p-value gives the fraction of training data with an MD greater than or equal to the one for the given sample, assuming normally distributed data. The smaller the p-value, the less trustworthy the prediciton. For highly non-normal X properties (e.g., fingerprints), the MD p-value is wildly inaccurate.

Structural Similar Compounds

Name	Nicardipine	Diltiazem	Moricizine
Structure	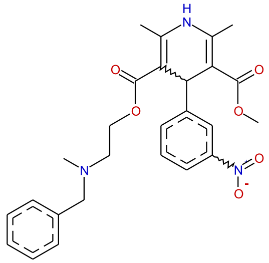	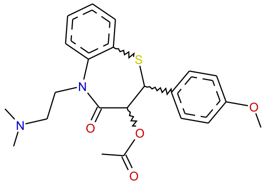	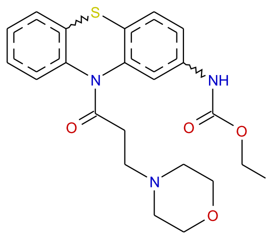
Actual Endpoint	Single-Carcinogen	Multiple-Carcinogen	Single-Carcinogen
Predicted Endpoint	Single-Carcinogen	Multiple-Carcinogen	Single-Carcinogen
Distance	0.632	0.748	0.749
Reference	US FDA (Centre for Drug Eval.& Res./Off. Testing & Res.) Sept. 1997	US FDA (Centre for Drug Eval.& Res./Off. Testing & Res.) Sept. 1997	US FDA (Centre for Drug Eval.& Res./Off. Testing & Res.) Sept. 1997

Model Applicability

Unknown features are fingerprint features in the query molecule, but not found or appearing too infrequently in the training set.

1. All properties and OPS components are within expected ranges.

Feature Contribution

Top features for positive contribution

Fingerprint	Bit/Smiles	Feature Structure	Score	Multiple-Carcinogen in training set
SCFP_4	-1065373877	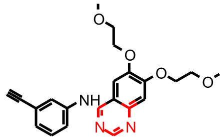 [*][c]1:[*]:[c](:[*]):n:[cH]:n:1	0.721	3 out of 3

SCFP_4	-1181430618	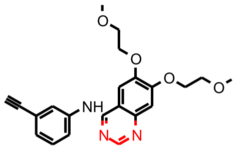 [*]:n:[cH]:n:[*]	0.663	4 out of 5
SCFP_4	2142015375	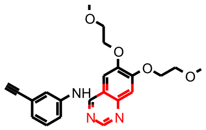 [*]:[cH]:[c]1:n:[cH]: n:[*]:[c]:1:[*]	0.433	2 out of 3

Top Features for negative contribution

Fingerprint	Bit/Smiles	Feature Structure	Score	Multiple-Carcinogen in training set
SCFP_4	622342378	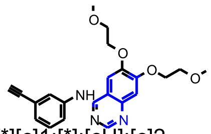 [*][c]1:[*]:[cH]:[c]2 :[c]([*]):[*]:[cH]:n :[c]:2:[cH]:1	-0.816	0 out of 4
SCFP_4	112346096	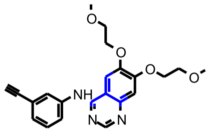 [*][c](:[*]):[c](:[cH]:[*]):[c](:[*]):[*]	-0.73	1 out of 10
SCFP_4	1242547645	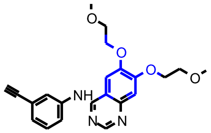 [*]CO[c]1:[cH]:[c](:[*]):[*]:[cH]:[c]:1O[*]	-0.489	0 out of 2

Compound 5

TOPKAT_Rat_Male_FDA_None_vs_Carcinogen

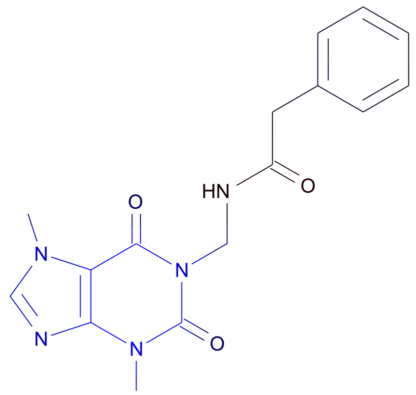

$C_{16}H_{17}N_5O_3$

Molecular Weight: 327.33788

ALogP: 0.773

Rotatable Bonds: 4

Acceptors: 4

Donors: 1

Model Prediction

Prediction: Non-Carcinogen

Probability: 0.192

Enrichment: 0.575

Bayesian Score: -7.89

Mahalanobis Distance: 12.5

Mahalanobis Distance p-value: 0.00654

Prediction: Positive if the Bayesian score is above the estimated best cutoff value from minimizing the false positive and false negative rate.
Probability: The esimated probability that the sample is in the positive category. This assumes that the Bayesian score follows a normal distribution and is different from the prediction using a cutoff.
Enrichment: An estimate of enrichment, that is, the increased likelihood (versus random) of this sample being in the category.
Bayesian Score: The standard Laplacian-modified Bayesian score.
Mahalanobis Distance: The Mahalanobis distance (MD) is the distance to the center of the training data. The larger the MD, the less trustworthy the prediction.
Mahalanobis Distance p-value: The p-value gives the fraction of training data with an MD greater than or equal to the one for the given sample, assuming normally distributed data. The smaller the p-value, the less trustworthy the prediciton. For highly non-normal X properties (e.g., fingerprints), the MD p-value is wildly inaccurate.

Structural Similar Compounds

Name	Pentoxifylline	Scopolamine	Fluconazole
Structure	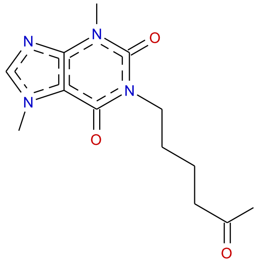	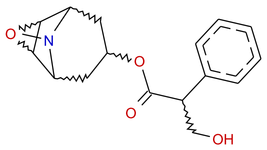	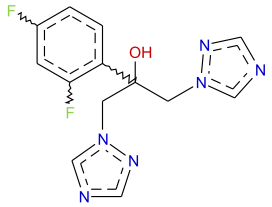
Actual Endpoint	Non-Carcinogen	Non-Carcinogen	Carcinogen
Predicted Endpoint	Non-Carcinogen	Non-Carcinogen	Carcinogen
Distance	0.464	0.565	0.568
Reference	US FDA (Centre for Drug Eval.& Res./Off. Testing & Res.) Sept. 1997	US FDA (Centre for Drug Eval.& Res./Off. Testing & Res.) Sept. 1997	US FDA (Centre for Drug Eval.& Res./Off. Testing & Res.) Sept. 1997

Model Applicability

Unknown features are fingerprint features in the query molecule, but not found or appearing too infrequently in the training set.

1. All properties and OPS components are within expected ranges.

Feature Contribution

Top features for positive contribution

Fingerprint	Bit/Smiles	Feature Structure	Score	Carcinogen in training set
SCFP_6	1394899299	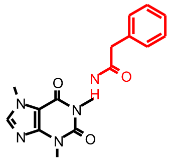 [*]NC(=O)C[c]1:[cH]:[cH]:[cH]:[cH]:[cH]:1	0.415	1 out of 1

SCFP_6	136358998	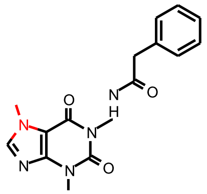<br>[*]:n(:[*])C	0.313	3 out of 6
SCFP_6	8	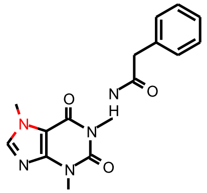<br>[*]n(:[*]):[*]	0.24	39 out of 95
**Top Features for negative contribution**				
**Fingerprint**	**Bit/Smiles**	**Feature Structure**	**Score**	**Carcinogen in training set**
SCFP_6	399659969	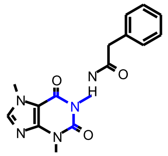<br>[*]CN(C(=[*])[*])C(=[*])[*]	-0.578	1 out of 8
SCFP_6	1653911926	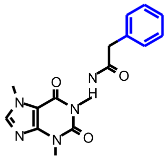<br>[*][c]1:[cH]:[cH]:[cH]:[cH]:[cH]:1	-0.504	12 out of 64
SCFP_6	1445006032	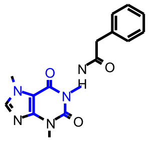<br>[*]CN1C(=[*])[*][c]2:[*]:[*]:n([*]):[c]:2C1=O	-0.496	0 out of 2

Erlotinib

TOPKAT_Rat_Male_FDA_None_vs_Carcinogen

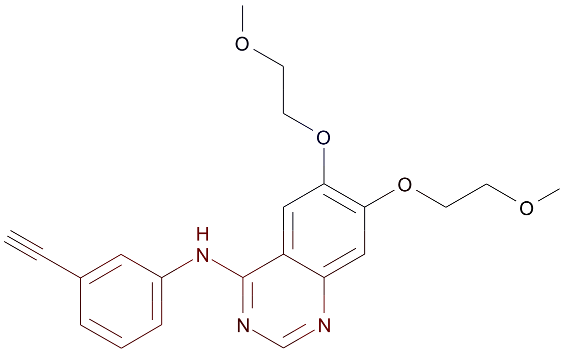

$C_{22}H_{23}N_3O_4$
Molecular Weight: 393.43572
ALogP: 4.309
Rotatable Bonds: 10
Acceptors: 7
Donors: 1

Model Prediction

Prediction: Carcinogen
Probability: 0.52
Enrichment: 1.56
Bayesian Score: 4.91
Mahalanobis Distance: 17.4
Mahalanobis Distance p-value: 2.91e-012

Prediction: Positive if the Bayesian score is above the estimated best cutoff value from minimizing the false positive and false negative rate.
Probability: The esimated probability that the sample is in the positive category. This assumes that the Bayesian score follows a normal distribution and is different from the prediction using a cutoff.
Enrichment: An estimate of enrichment, that is, the increased likelihood (versus random) of this sample being in the category.
Bayesian Score: The standard Laplacian-modified Bayesian score.
Mahalanobis Distance: The Mahalanobis distance (MD) is the distance to the center of the training data. The larger the MD, the less trustworthy the prediction.
Mahalanobis Distance p-value: The p-value gives the fraction of training data with an MD greater than or equal to the one for the given sample, assuming normally distributed data. The smaller the p-value, the less trustworthy the prediciton. For highly non-normal X properties (e.g., fingerprints), the MD p-value is wildly inaccurate.

Structural Similar Compounds

Name	Mycophenolate	Nicardipine	Nimodipine
Structure	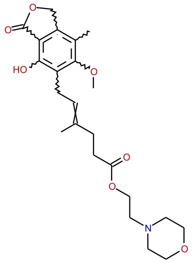	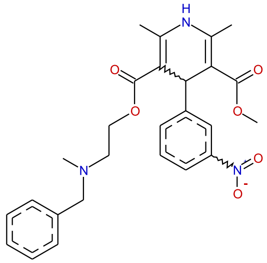	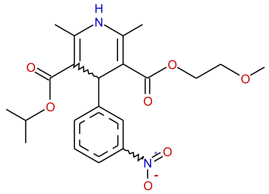
Actual Endpoint	Non-Carcinogen	Carcinogen	Carcinogen
Predicted Endpoint	Non-Carcinogen	Carcinogen	Carcinogen
Distance	0.624	0.667	0.676
Reference	US FDA (Centre for Drug Eval.& Res./Off. Testing & Res.) Sept. 1997	US FDA (Centre for Drug Eval.& Res./Off. Testing & Res.) Sept. 1997	US FDA (Centre for Drug Eval.& Res./Off. Testing & Res.) Sept. 1997

Model Applicability

Unknown features are fingerprint features in the query molecule, but not found or appearing too infrequently in the training set.

1. All properties and OPS components are within expected ranges.

Feature Contribution

Top features for positive contribution

Fingerprint	Bit/Smiles	Feature Structure	Score	Carcinogen in training set
SCFP_6	123285475	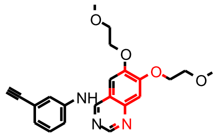 [*]O[c]1:[cH]:[c](:n:[*]):[c](:[*]):[*]:[c]:1[*]	0.429	3 out of 5

SCFP_6	-1065373877	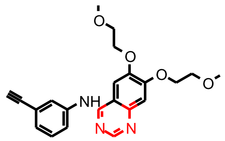 [*][c]1:[*]:[c](:[*]) :n:[cH]:n:1	0.429	3 out of 5
SCFP_6	-2020651081	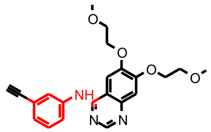 [*][c]1:[cH]:[cH]:[cH]:[c](N[c](:[*]):[*]):[cH]:1	0.415	1 out of 1

Top Features for negative contribution

Fingerprint	Bit/Smiles	Feature Structure	Score	Carcinogen in training set
SCFP_6	125474664	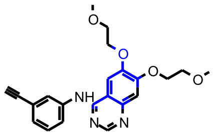 [*]O[c]1:[cH]:[c](:[c]([*]):[*]):[c](:[*]):[*]:[c]:1[*]	-0.484	1 out of 7
SCFP_6	-417738003	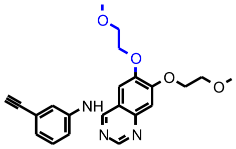 [*]OCCOC	-0.264	1 out of 5
SCFP_6	951581613	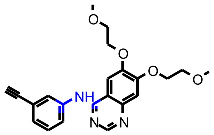 [*]:[c](:[*])N[c](:[*]):[*]	-0.132	1 out of 4

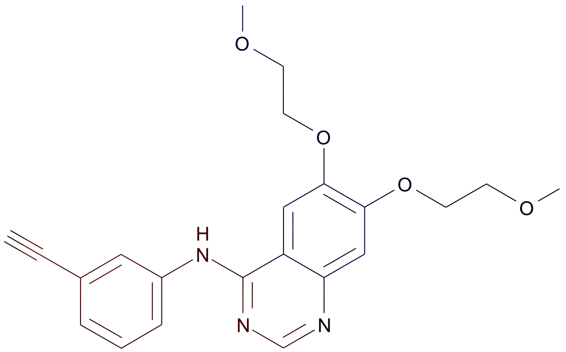

$C_{22}H_{23}N_3O_4$
Molecular Weight: 393.43572
ALogP: 4.309
Rotatable Bonds: 10
Acceptors: 7
Donors: 1

Model Prediction

Prediction: Multiple-Carcinogen
Probability: 0.568
Enrichment: 1.37
Bayesian Score: 0.821
Mahalanobis Distance: 16.5
Mahalanobis Distance p-value: 4.64e-007

Prediction: Positive if the Bayesian score is above the estimated best cutoff value from minimizing the false positive and false negative rate.
Probability: The esimated probability that the sample is in the positive category. This assumes that the Bayesian score follows a normal distribution and is different from the prediction using a cutoff.
Enrichment: An estimate of enrichment, that is, the increased likelihood (versus random) of this sample being in the category.
Bayesian Score: The standard Laplacian-modified Bayesian score.
Mahalanobis Distance: The Mahalanobis distance (MD) is the distance to the center of the training data. The larger the MD, the less trustworthy the prediction.
Mahalanobis Distance p-value: The p-value gives the fraction of training data with an MD greater than or equal to the one for the given sample, assuming normally distributed data. The smaller the p-value, the less trustworthy the prediciton. For highly non-normal X properties (e.g., fingerprints), the MD p-value is wildly inaccurate.

Structural Similar Compounds

Name	Nicardipine	Nimodipine	Felodipine
Structure	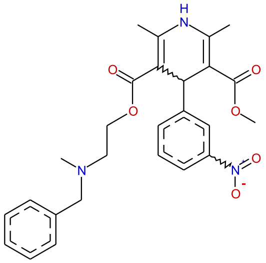	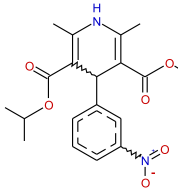	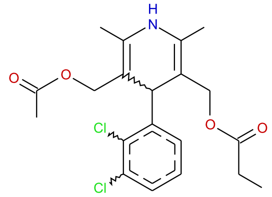
Actual Endpoint	Single-Carcinogen	Single-Carcinogen	Single-Carcinogen
Predicted Endpoint	Single-Carcinogen	Single-Carcinogen	Single-Carcinogen
Distance	0.685	0.700	0.721
Reference	US FDA (Centre for Drug Eval.& Res./Off. Testing & Res.) Sept. 1997	US FDA (Centre for Drug Eval.& Res./Off. Testing & Res.) Sept. 1997	US FDA (Centre for Drug Eval.& Res./Off. Testing & Res.) Sept. 1997

Model Applicability

Unknown features are fingerprint features in the query molecule, but not found or appearing too infrequently in the training set.

1. All properties and OPS components are within expected ranges.

Feature Contribution

Top features for positive contribution

Fingerprint	Bit/Smiles	Feature Structure	Score	Multiple-Carcinogen in training set
SCFP_8	-1065373877	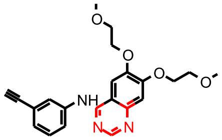 [*][c]1:[*]:[c](:[*]):n:[cH]:n:1	0.649	3 out of 3

SCFP_8	2	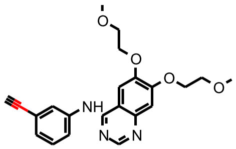 [*]C#[*]	0.584	6 out of 8
SCFP_8	-1181430618	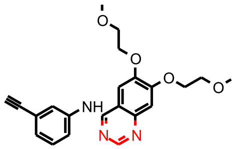 [*]:n:[cH]:n:[*]	0.453	4 out of 6

Top Features for negative contribution

Fingerprint	Bit/Smiles	Feature Structure	Score	Multiple-Carcinogen in training set
SCFP_8	136239834	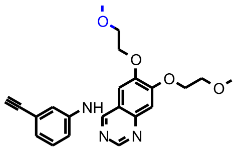 [*]OC	-0.358	3 out of 13
SCFP_8	129215346	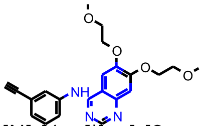 [*]N[c]1:n:[*]:n:[c]2:[cH]:[*]:[c]([*]):[cH]:[c]:1:2	-0.31	0 out of 1
SCFP_8	-417738003	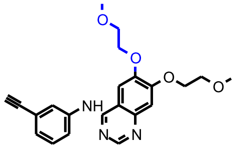 [*]OCCOC	-0.31	0 out of 1

Compound 5

TOPKAT_Skin_Irritancy_None_vs_Irritant

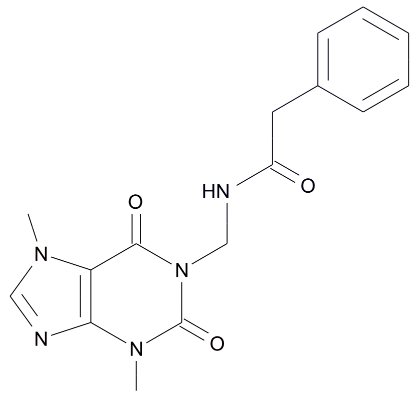

$C_{16}H_{17}N_5O_3$

Molecular Weight: 327.33788

ALogP: 0.773

Rotatable Bonds: 4

Acceptors: 4

Donors: 1

Structural Similar Compounds

Name	1-(Butylsulfonyl)-4-pipecoline	Benzothiazolesulfonamide, N-(1-methylethyl)-	Benzenesulfonic acid, 3-(diethylamino)-, sodium salt
Structure	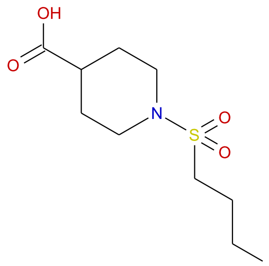	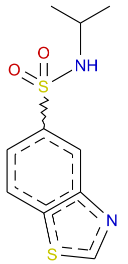	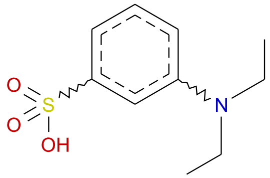
Actual Endpoint	Irritant	Irritant	Irritant
Predicted Endpoint	Non-Irritant	Non-Irritant	Non-Irritant
Distance	0.678	0.710	0.711
Reference	US ARMY	AMIHBC AMA Archives of Industrial Hygiene and Occupational Medicine. (Chicago , IL) V.2-10, 1950-54. For publisher information, see AEHLAU. Volume(issue)/pag e/year: 5,311,1952	85JCAE "Prehled Prumyslove Toxikologie; Organicke Latky," Marhold, J., Prague , Czechoslovakia, Avicenum, 1986 Volume(issue)/page/year: -,1056,1986

Model Prediction

Prediction: Non-Irritant

Probability: 0.971

Enrichment: 1.05

Bayesian Score: -0.948

Mahalanobis Distance: 10.8

Mahalanobis Distance p-value: 0.00577

Prediction: Positive if the Bayesian score is above the estimated best cutoff value from minimizing the false positive and false negative rate.
Probability: The esimated probability that the sample is in the positive category. This assumes that the Bayesian score follows a normal distribution and is different from the prediction using a cutoff.
Enrichment: An estimate of enrichment, that is, the increased likelihood (versus random) of this sample being in the category.
Bayesian Score: The standard Laplacian-modified Bayesian score.
Mahalanobis Distance: The Mahalanobis distance (MD) is the distance to the center of the training data. The larger the MD, the less trustworthy the prediction.
Mahalanobis Distance p-value: The p-value gives the fraction of training data with an MD greater than or equal to the one for the given sample, assuming normally distributed data. The smaller the p-value, the less trustworthy the prediciton. For highly non-normal X properties (e.g., fingerprints), the MD p-value is wildly inaccurate.

Model Applicability

Unknown features are fingerprint features in the query molecule, but not found or appearing too infrequently in the training set.

1. All properties and OPS components are within expected ranges.
2. Unknown FCFP_2 feature: 136150461: [*]:n(:[*])C

Feature Contribution

Top features for positive contribution

Fingerprint	Bit/Smiles	Feature Structure	Score	Irritant in training set

FCFP_12	-1986158408	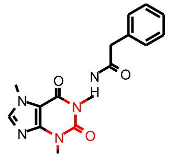 [*]N([*])C(=O)N([*])[*]	0.0821	13 out of 13
FCFP_12	-1539132615	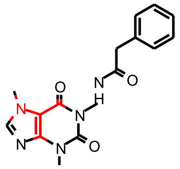 [*]n1:[*]:[*]:[c]([*]):[c]:1C(=[*])[*]	0.0795	9 out of 9
FCFP_12	-1410049896	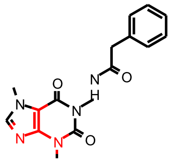 [*]N([*])[c]1:n:[*]:[*]:[c]:1[*]	0.0734	5 out of 5
**Top Features for negative contribution**				
**Fingerprint**	**Bit/Smiles**	**Feature Structure**	**Score**	**Irritant in training set**
FCFP_12	566058135	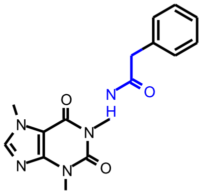 [*]CC(=O)N[*]	-0.367	13 out of 21
FCFP_12	675769755	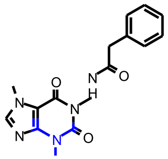 [*]C(=[*])N(C)[c](:[*]):[*]	-0.088	15 out of 18

FCFP_12	1618154665	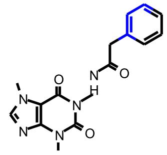 [*][c](:[*]):[cH]:[cH]:[*]	-0.0845	412 out of 490

Erlotinib

TOPKAT_Skin_Irritancy_None_vs_Irritant

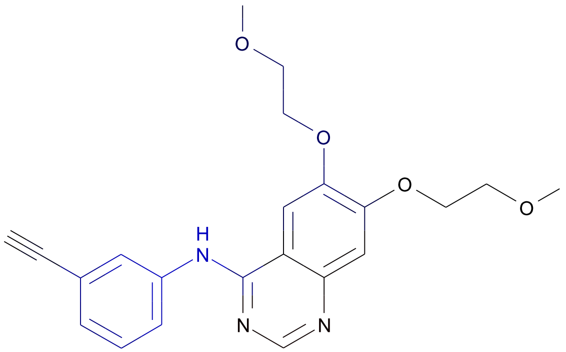

$C_{22}H_{23}N_3O_4$

Molecular Weight: 393.43572

ALogP: 4.309

Rotatable Bonds: 10

Acceptors: 7

Donors: 1

Model Prediction

Prediction: Non-Irritant

Probability: 0.119

Enrichment: 0.13

Bayesian Score: -5.76

Mahalanobis Distance: 10.2

Mahalanobis Distance p-value: 0.0333

Prediction: Positive if the Bayesian score is above the estimated best cutoff value from minimizing the false positive and false negative rate.
Probability: The esimated probability that the sample is in the positive category. This assumes that the Bayesian score follows a normal distribution and is different from the prediction using a cutoff.
Enrichment: An estimate of enrichment, that is, the increased likelihood (versus random) of this sample being in the category.
Bayesian Score: The standard Laplacian-modified Bayesian score.
Mahalanobis Distance: The Mahalanobis distance (MD) is the distance to the center of the training data. The larger the MD, the less trustworthy the prediction.
Mahalanobis Distance p-value: The p-value gives the fraction of training data with an MD greater than or equal to the one for the given sample, assuming normally distributed data. The smaller the p-value, the less trustworthy the prediciton. For highly non-normal X properties (e.g., fingerprints), the MD p-value is wildly inaccurate.

Structural Similar Compounds

Name	Propanoic acid, 2-(4-((5-(trifluoromethyl)-2-pyridinyl)oxy)phenoxy) -, butyl ester	Carbamic acid, ((dibutylamino)thio)methyl-, 2,2-dimethyl-2,3-dihydro-7-benzofuranyl ester	1,4-Pentadien-3-one, 1,5-bis(p-azidophenyl)-
Structure	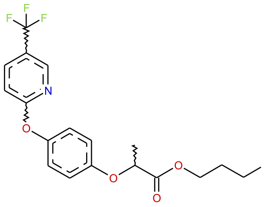	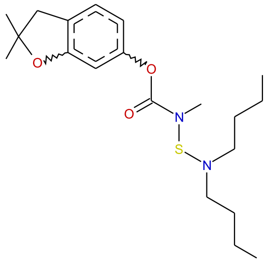	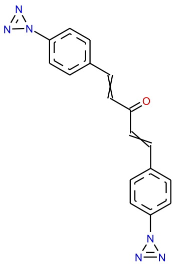
Actual Endpoint	Irritant	Irritant	Irritant
Predicted Endpoint	Irritant	Irritant	Irritant
Distance	0.776	0.777	0.803
Reference	NNGADV Nippon Noyaku Gakkaishi. Journal of the Pesticide Science Society of Japan. (Nippon Noyaku Gakkai, 1-43-11, Komagome, Toshima-ku, Tokyo 170, Japan) V.1- 1976- Volume(issue)/page/year: 15,305,1990	NTIS** National Technical Information Service. (Springfield, VA 22161) Forme rly U.S. Clearinghouse for Scientific & Technical Information. Volume(issue)/pag e/year: OTS0539690	85JCAE "Prehled Prumyslove Toxikologie; Organicke Latky," Marhold, J., Prague , Czechoslovakia, Avicenum, 1986 Volume(issue)/page/year: -,733,1986

Model Applicability

Unknown features are fingerprint features in the query molecule, but not found or appearing too infrequently in the training set.

1. All properties and OPS components are within expected ranges.
2. Unknown FCFP_2 feature: 902193919: [*]:[c](:[*])C#C

Feature Contribution

Top features for positive contribution				
Fingerprint	Bit/Smiles	Feature Structure	Score	Irritant in training set

FCFP_12	-124685461	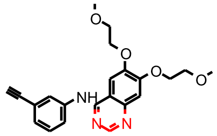 [*]n1:[*]:[*]:n:[cH]:1	0.0734	5 out of 5
FCFP_12	-475316933	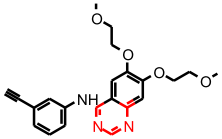 [*][c]1:[*]:[c](:[*]):n:[cH]:n:1	0.0703	4 out of 4
FCFP_12	-1385265413	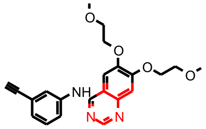 [*]:[cH]:[c]1:n:[cH]:n:[*]:[c]:1:[*]	0.0658	3 out of 3

Top Features for negative contribution

Fingerprint	Bit/Smiles	Feature Structure	Score	Irritant in training set
FCFP_12	411414971	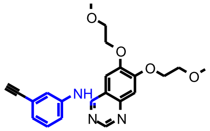 [*][c]1:[cH]:[cH]:[cH]:[c](N[c](:[*]):[*]):[cH]:1	-1.31	1 out of 7
FCFP_12	-1059904848	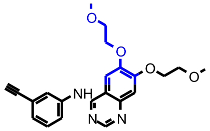 [*][c](:[*]):[c](OCCOC):[cH]:[*]	-1.04	0 out of 2

FCFP_12	839741273	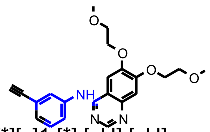 [*][c]1:[*]:[cH]:[cH]:[c](N[c](:[*]):[*]):[cH]:1	-0.708	4 out of 10

Compound 5

TOPKAT_Carcinogenic_Potency_TD50_Mouse

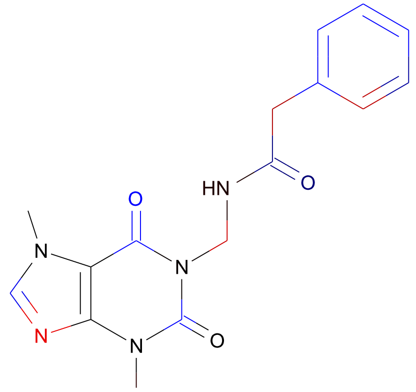

$C_{16}H_{17}N_5O_3$
Molecular Weight: 327.33788
ALogP: 0.773
Rotatable Bonds: 4
Acceptors: 4
Donors: 1

Model Prediction

Prediction: 70.9
Unit: mg/kg_body_weight/day
Mahalanobis Distance: 11
Mahalanobis Distance p-value: 0.00169

Mahalanobis Distance: The Mahalanobis distance (MD) is a generalization of the Euclidean distance that accounts for correlations among the X properties. It is calculated as the distance to the center of the training data. The larger the MD, the less trustworthy the prediction.
Mahalanobis Distance p-value: The p-value gives the fraction of training data with an MD greater than or equal to the one for the given sample, assuming normally distributed data. The smaller the p-value, the less trustworthy the prediciton. For highly non-normal X properties (e.g., fingerprints), the MD p-value is wildly inaccurate.

Structural Similar Compounds

Name	3-Benzylsydnone-4-acetamide	Ripazepam	422
Structure	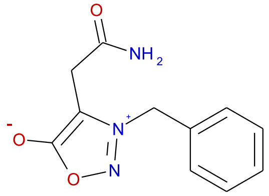	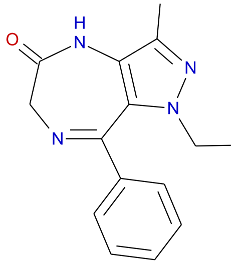	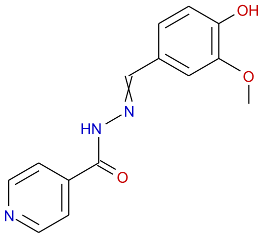
Actual Endpoint (-log C)	4.35917	3.37174	3.99565
Predicted Endpoint (-log C)	3.78539	3.42826	3.22211
Distance	0.615	0.628	0.654
Reference	CPDB	CPDB	CPDB

Model Applicability

Unknown features are fingerprint features in the query molecule, but not found or appearing too infrequently in the training set.

1. All properties and OPS components are within expected ranges.
2. Unknown ECFP_2 feature: 1135573248: [*]N([*])C(=O)N([*])[*]
3. Unknown ECFP_2 feature: -661097313: [*]CN(C(=[*])[*])C(=[*])[*]
4. Unknown ECFP_2 feature: -960717516: [*]C(=[*])N(C)[c](:[*]):[*]
5. Unknown ECFP_2 feature: -39630146: [*]NCN([*])[*]

Feature Contribution

Top features for positive contribution

Fingerprint	Bit/Smiles	Feature Structure	Score
ECFP_6	655739385	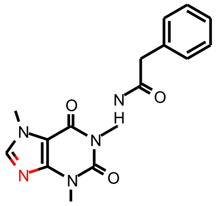 [*]:n:[*]	0.229

ECFP_6	1559650422	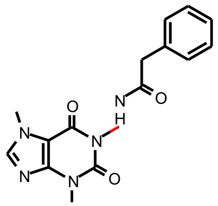 [*]C[*]	0.203
ECFP_6	-2024255407	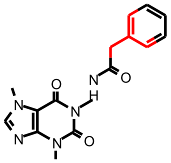 [*]C[c](:[cH]:[*]):[cH]:[*]	0.172
**Top Features for negative contribution**			
**Fingerprint**	**Bit/Smiles**	**Feature Structure**	**Score**
ECFP_6	2106656448	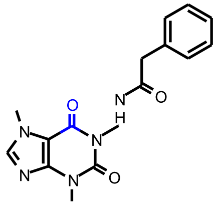 [*]C(=O)[*]	-0.275
ECFP_6	1996767644	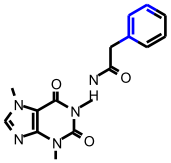 [*][c](:[*]):[cH]:[cH]:[*]	-0.251
ECFP_6	642810091	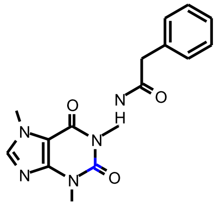 [*]C(=[*])[*]	-0.247

Erlotinib

TOPKAT_Carcinogenic_Potency_TD50_Mouse

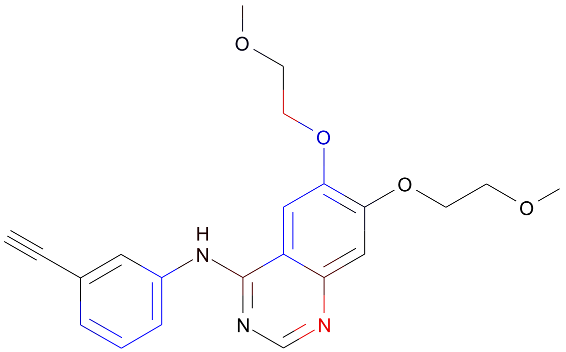

$C_{22}H_{23}N_3O_4$

Molecular Weight: 393.43572

ALogP: 4.309

Rotatable Bonds: 10

Acceptors: 7

Donors: 1

Model Prediction

Prediction: 39.8

Unit: mg/kg_body_weight/day

Mahalanobis Distance: 16.1

Mahalanobis Distance p-value: 1.15e-016

Mahalanobis Distance: The Mahalanobis distance (MD) is a generalization of the Euclidean distance that accounts for correlations among the X properties. It is calculated as the distance to the center of the training data. The larger the MD, the less trustworthy the prediction.
Mahalanobis Distance p-value: The p-value gives the fraction of training data with an MD greater than or equal to the one for the given sample, assuming normally distributed data. The smaller the p-value, the less trustworthy the prediciton. For highly non-normal X properties (e.g., fingerprints), the MD p-value is wildly inaccurate.

Structural Similar Compounds

Name	Compound LY171883	832	5,5´-(1,1´-Biphenyl)-2,5-dyl-bis (oxy)(2,2-dimethylpentanoic acid)
Structure	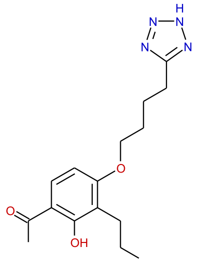	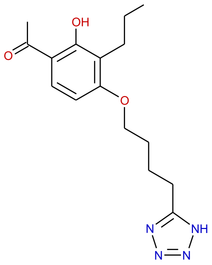	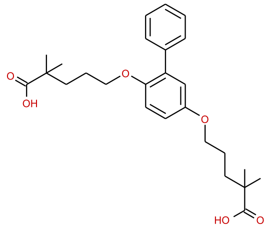
Actual Endpoint (-log C)	3.45372	3.45372	3.90166
Predicted Endpoint (-log C)	2.84749	2.80429	2.75893
Distance	0.772	0.782	0.796
Reference	CPDB	CPDB	CPDB

Model Applicability

Unknown features are fingerprint features in the query molecule, but not found or appearing too infrequently in the training set.

1. OPS PC13 out of range. Value: -3.2209. Training min, max, SD, explained variance: -3.068, 3.6909, 1.329, 0.0220.
2. Unknown ECFP_2 feature: -1253653003: [*]COC
3. Unknown ECFP_2 feature: -182178874: [*]#C[c](:c:[*]):c:[*]
4. Unknown ECFP_2 feature: 1139738044: [*]:[c](:[*])C#C

Feature Contribution

Top features for positive contribution			
**Fingerprint**	**Bit/Smiles**	**Feature Structure**	**Score**
ECFP_6	655739385	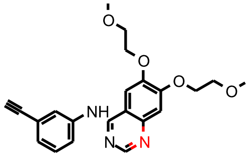 [*]:n:[*]	0.229

ECFP_6	1559650422	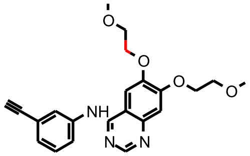 [*]C[*]	0.203
ECFP_6	1333660716	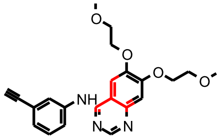 [*][c](:[*]):[c](:[cH]:[*]):[c](:[*]):[*]	0.0746

Top Features for negative contribution

Fingerprint	Bit/Smiles	Feature Structure	Score
ECFP_6	1996767644	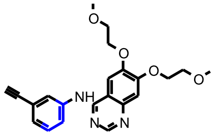 [*][c](:[*]):[cH]:[cH]:[*]	-0.251
ECFP_6	642810091	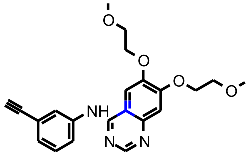 [*]C(=[*])[*]	-0.247
ECFP_6	-182236392	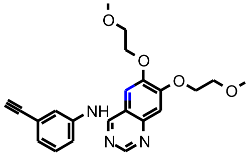 [*]:[cH]:[*]	-0.232

Compound 5

TOPKAT_Carcinogenic_Potency_TD50_Rat

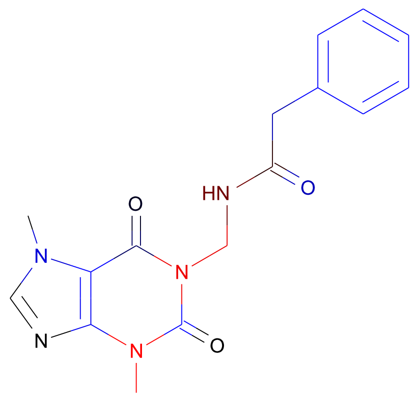

$C_{16}H_{17}N_5O_3$

Molecular Weight: 327.33788

ALogP: 0.773

Rotatable Bonds: 4

Acceptors: 4

Donors: 1

Model Prediction

Prediction: 55.7

Unit: mg/kg_body_weight/day

Mahalanobis Distance: 14.7

Mahalanobis Distance p-value: 2.33e-010

Mahalanobis Distance: The Mahalanobis distance (MD) is a generalization of the Euclidean distance that accounts for correlations among the X properties. It is calculated as the distance to the center of the training data. The larger the MD, the less trustworthy the prediction.
Mahalanobis Distance p-value: The p-value gives the fraction of training data with an MD greater than or equal to the one for the given sample, assuming normally distributed data. The smaller the p-value, the less trustworthy the prediciton. For highly non-normal X properties (e.g., fingerprints), the MD p-value is wildly inaccurate.

Structural Similar Compounds

Name	Fluconazole	4,4´-Sulfonylbisacetanilide	3-Benzylsydnone-4-acetamide
Structure	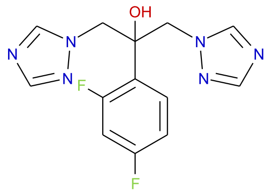	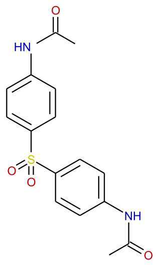	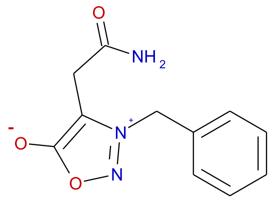
Actual Endpoint (-log C)	3.57923	3.77655	4.74041
Predicted Endpoint (-log C)	4.1264	3.55337	2.85943
Distance	0.604	0.632	0.636
Reference	CPDB	CPDB	CPDB

Model Applicability

Unknown features are fingerprint features in the query molecule, but not found or appearing too infrequently in the training set.

1. All properties and OPS components are within expected ranges.

Feature Contribution

Top features for positive contribution

Fingerprint	Bit/Smiles	Feature Structure	Score
FCFP_6	136627117	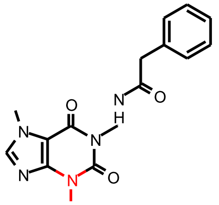 [*]N([*])C	0.69

FCFP_6	1	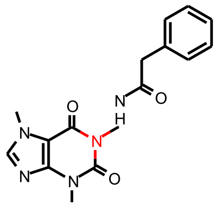 [*]N([*])[*]	0.234
FCFP_6	-885550502	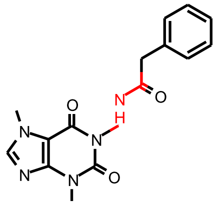 [*]CNC(=[*])[*]	0.229

Top Features for negative contribution

Fingerprint	Bit/Smiles	Feature Structure	Score
FCFP_6	991735244	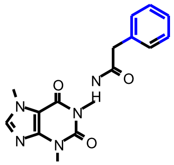 [*][c]1:[*]:[cH]:[cH]:[cH]:[cH]:1	-0.422
FCFP_6	-2093839777	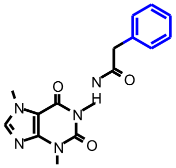 [*][c]1:[cH]:[cH]:[cH]:[cH]:[cH]:1	-0.378
FCFP_6	16	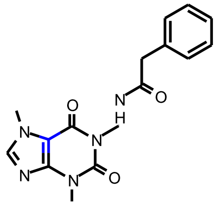 [*][c](:[*]):[*]	-0.354

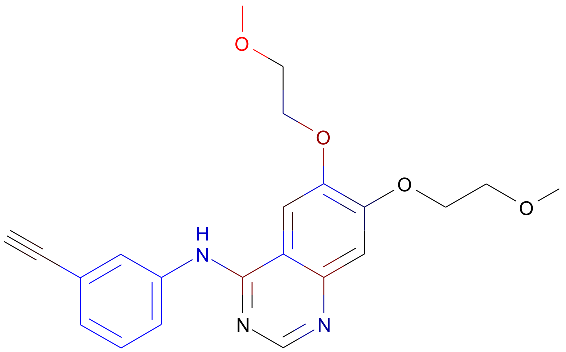

$C_{22}H_{23}N_3O_4$
Molecular Weight: 393.43572
ALogP: 4.309
Rotatable Bonds: 10
Acceptors: 7
Donors: 1

Model Prediction

Prediction: 8.06
Unit: mg/kg_body_weight/day
Mahalanobis Distance: 17.7
Mahalanobis Distance p-value: 3.99e-020

Mahalanobis Distance: The Mahalanobis distance (MD) is a generalization of the Euclidean distance that accounts for correlations among the X properties. It is calculated as the distance to the center of the training data. The larger the MD, the less trustworthy the prediction.
Mahalanobis Distance p-value: The p-value gives the fraction of training data with an MD greater than or equal to the one for the given sample, assuming normally distributed data. The smaller the p-value, the less trustworthy the prediciton. For highly non-normal X properties (e.g., fingerprints), the MD p-value is wildly inaccurate.

Structural Similar Compounds

Name	Loxtidine	C.I. direct brown 95	Omeprazole
Structure	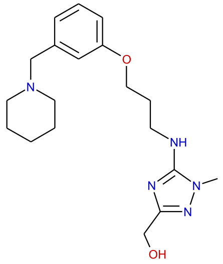	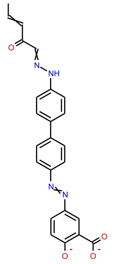	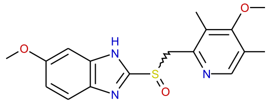
Actual Endpoint (-log C)	2.87532	5.31387	3.4628
Predicted Endpoint (-log C)	3.63996	4.30266	4.7324
Distance	0.685	0.715	0.741
Reference	CPDB	CPDB	CPDB

Model Applicability

Unknown features are fingerprint features in the query molecule, but not found or appearing too infrequently in the training set.

1. All properties and OPS components are within expected ranges.
2. Unknown FCFP_2 feature: 902193919: [*]:[c](:[*])C#C

Feature Contribution

Top features for positive contribution

Fingerprint	Bit/Smiles	Feature Structure	Score
FCFP_6	136627117	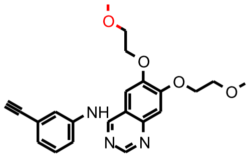 [*]N([*])C	0.69

FCFP_6	1	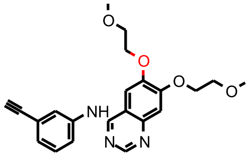 [*]N([*])[*]	0.234
FCFP_6	203677720	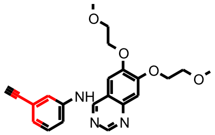 [*]C[c](:[cH]:[*]):[cH]:[*]	0.137
**Top Features for negative contribution**			
**Fingerprint**	**Bit/Smiles**	**Feature Structure**	**Score**
FCFP_6	991735244	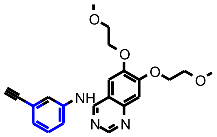 [*][c]1:[*]:[cH]:[cH]:[cH]:[cH]:1	-0.422
FCFP_6	16	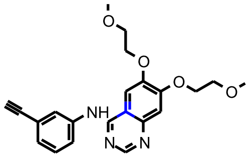 [*][c](:[*]):[*]	-0.354
FCFP_6	590925877	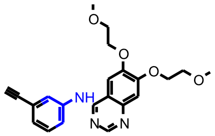 [*]N[c](:[cH]:[*]):[cH]:[*]	-0.323

Compound 5

TOPKAT_Chronic_LOAEL

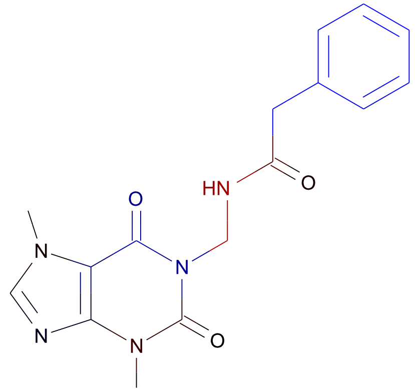

$C_{16}H_{17}N_5O_3$

Molecular Weight: 327.33788

ALogP: 0.773

Rotatable Bonds: 4

Acceptors: 4

Donors: 1

Structural Similar Compounds

Name	PENTOXIFYLLINE	TRIAMIPHOS	DANTROLENE.NA
Structure	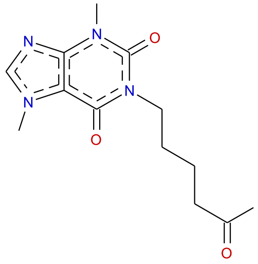	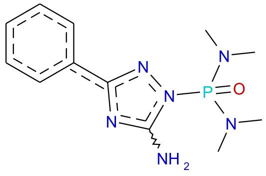	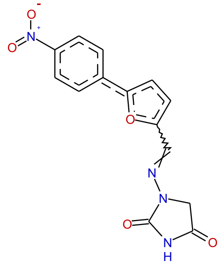
Actual Endpoint (-log C)	3.26844	6.99166	4.19625
Predicted Endpoint (-log C)	3.43031	4.6967	4.62637
Distance	0.468	0.478	0.562
Reference	NDA-18534	RQTOX ECAO CIN R525;8801;(4)	NDA-17443

Model Prediction

Prediction: 0.0392

Unit: g/kg_body_weight

Mahalanobis Distance: 30.7

Mahalanobis Distance p-value: 6.86e-026

Mahalanobis Distance: The Mahalanobis distance (MD) is a generalization of the Euclidean distance that accounts for correlations among the X properties. It is calculated as the distance to the center of the training data. The larger the MD, the less trustworthy the prediction.

Mahalanobis Distance p-value: The p-value gives the fraction of training data with an MD greater than or equal to the one for the given sample, assuming normally distributed data. The smaller the p-value, the less trustworthy the prediciton. For highly non-normal X properties (e.g., fingerprints), the MD p-value is wildly inaccurate.

Model Applicability

Unknown features are fingerprint features in the query molecule, but not found or appearing too infrequently in the training set.

1. All properties and OPS components are within expected ranges.
2. Unknown FCFP_2 feature: 470101049: [*]NCN([*])[*]
3. Unknown ECFP_6 feature: 672362763: [*]n(:[*]):[*]
4. Unknown ECFP_6 feature: 1135573248: [*]N([*])C(=O)N([*])[*]
5. Unknown ECFP_6 feature: -661097313: [*]CN(C(=[*])[*])C(=[*])[*]
6. Unknown ECFP_6 feature: 1945129186: [*]N([*])C(=O)[c](:[*]):[*]
7. Unknown ECFP_6 feature: -813242890: [*]n1:[*]:[*]:[c]([*]):[c]:1C(=[*])[*]
8. Unknown ECFP_6 feature: -509950643: [*]N([*])[c]1:n:[*]:[*]:[c]:1[*]
9. Unknown ECFP_6 feature: -960717516: [*]C(=[*])N(C)[c](:[*]):[*]
10. Unknown ECFP_6 feature: -407983022: [*][c]1:[*]:[*]:[cH]:n:1C
11. Unknown ECFP_6 feature: -708878603: [*]n1:[*]:[*]:n:[cH]:1
12. Unknown ECFP_6 feature: -677309799: [*][c]1:[*]:[*]:[cH]:n:1
13. Unknown ECFP_6 feature: 866343404: [*]N([*])C
14. Unknown ECFP_6 feature: 866450950: [*]:n(:[*])C
15. Unknown ECFP_6 feature: -39630146: [*]NCN([*])[*]
16. Unknown ECFP_6 feature: 497523368: [*]CNC(=[*])[*]
17. Unknown ECFP_6 feature: 1731843802: [*]CC(=O)N[*]
18. Unknown ECFP_6 feature: 771857573: [*]C(=[*])C[c](:[*]):[*]
19. Unknown ECFP_6 feature: 1997021792: [*]:[cH]:[cH]:[cH]:[*]

Feature Contribution

Top features for positive contribution

Fingerprint	Bit/Smiles	Feature Structure	Score
ECFP_6	1559650422	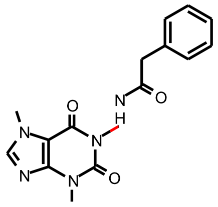 [*]C[*]	0.129
FCFP_6	3	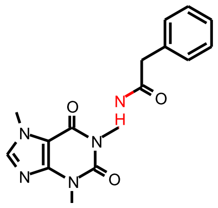 [*]N[*]	0.0924
FCFP_6	-2093839777	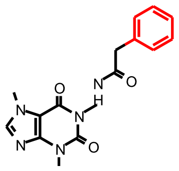 [*][c]1:[cH]:[cH]:[cH]:[cH]:[cH]:1	0.078

Top Features for negative contribution

Fingerprint	Bit/Smiles	Feature Structure	Score
FCFP_6	991735244	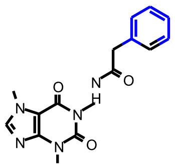 [*][c]1:[*]:[cH]:[cH]:[cH]:[cH]:1	-0.134

ECFP_6	1564392544	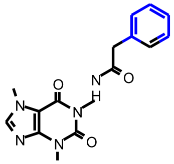 [*][c]1:[*]:[cH]:[cH]:[cH]:[cH]:1	-0.133
ECFP_6	2106656448	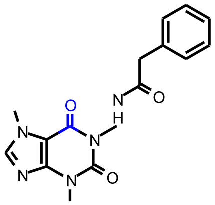 [*]C(=O)[*]	-0.11

Erlotinib

TOPKAT_Chronic_LOAEL

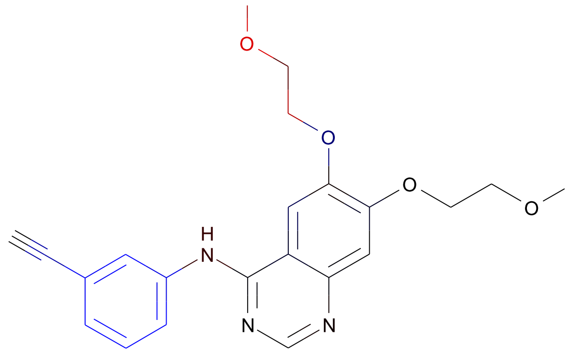

$C_{22}H_{23}N_3O_4$

Molecular Weight: 393.43572

ALogP: 4.309

Rotatable Bonds: 10

Acceptors: 7

Donors: 1

Model Prediction

Prediction: 0.0359

Unit: g/kg_body_weight

Mahalanobis Distance: 34.8

Mahalanobis Distance p-value: 2.91e-033

Mahalanobis Distance: The Mahalanobis distance (MD) is a generalization of the Euclidean distance that accounts for correlations among the X properties. It is calculated as the distance to the center of the training data. The larger the MD, the less trustworthy the prediction.
Mahalanobis Distance p-value: The p-value gives the fraction of training data with an MD greater than or equal to the one for the given sample, assuming normally distributed data. The smaller the p-value, the less trustworthy the prediciton. For highly non-normal X properties (e.g., fingerprints), the MD p-value is wildly inaccurate.

Structural Similar Compounds

Name	ASSURE	DILTIAZEM	RHODAMINE 6G
Structure	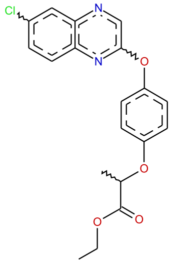	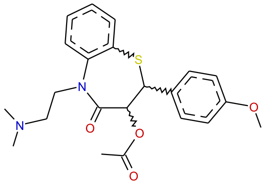	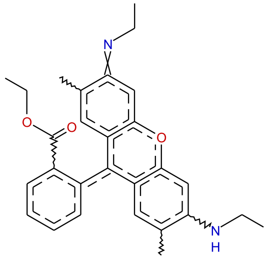
Actual Endpoint (-log C)	5.00328	4.21961	4.54906
Predicted Endpoint (-log C)	4.27671	4.005	4.6787
Distance	0.611	0.676	0.689
Reference	EPA COVER SHEET 0335;891001;(1)	NDA-18602	NTP 364 39

Model Applicability

Unknown features are fingerprint features in the query molecule, but not found or appearing too infrequently in the training set.

1. All properties and OPS components are within expected ranges.
2. Unknown FCFP_2 feature: 902193919: [*]:[c](:[*])C#C
3. Unknown FCFP_2 feature: 131784192: [*]C#C
4. Unknown ECFP_6 feature: -1114776580: [*]C#[*]
5. Unknown ECFP_6 feature: -1939823063: [*]#C
6. Unknown ECFP_6 feature: -1255706725: [*]CO[c](:[*]):[*]
7. Unknown ECFP_6 feature: -1253653003: [*]COC
8. Unknown ECFP_6 feature: -1242906247: [*]:[c](:[*])N[c](:[*]):[*]
9. Unknown ECFP_6 feature: -677309799: [*][c]1:[*]:[*]:[cH]:n:1
10. Unknown ECFP_6 feature: 1333660716: [*][c](:[*]):[c](:[cH]:[*]):[c](:[*]):[*]
11. Unknown ECFP_6 feature: 1410041175: [*]:[cH]:[c](:n:[*]):[c](:[*]):[*]
12. Unknown ECFP_6 feature: 1049768340: [*]N[c](:n:[*]):[c](:[*]):[*]
13. Unknown ECFP_6 feature: -1790412586: [*]CCO[*]
14. Unknown ECFP_6 feature: -177077903: [*]N[c](:[cH]:[*]):[cH]:[*]
15. Unknown ECFP_6 feature: -710237522: [*]:n:[cH]:n:[*]
16. Unknown ECFP_6 feature: -182178874: [*]#C[c](:[cH]:[*]):[cH]:[*]
17. Unknown ECFP_6 feature: 1997021792: [*]:[cH]:[cH]:[cH]:[*]
18. Unknown ECFP_6 feature: 1139738044: [*]:[c](:[*])C#C
19. Unknown ECFP_6 feature: -1545539812: [*]C#C

Feature Contribution

Top features for positive contribution			
**Fingerprint**	**Bit/Smiles**	**Feature Structure**	**Score**
FCFP_6	-1143715940	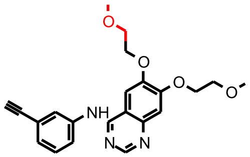 [*]COC	0.13
ECFP_6	1559650422	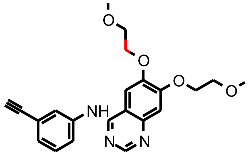 [*]C[*]	0.129
FCFP_6	3	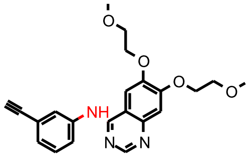 [*]N[*]	0.0924
**Top Features for negative contribution**			
**Fingerprint**	**Bit/Smiles**	**Feature Structure**	**Score**
FCFP_6	991735244	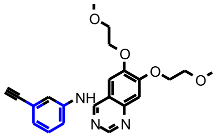 [*][c]1:[*]:[cH]:[cH]:[cH]:[cH]:1	-0.134

FCFP_6	1	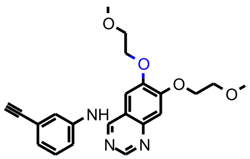<br>[*]N([*])[*]	-0.102
FCFP_6	-453677277	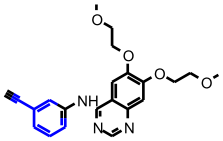<br>[*]C[c]1:[cH]:[*]:[cH<br>]:[cH]:[cH]:1	-0.0906

Compound 5

TOPKAT_Rat_Maximum_Tolerated_Dose_Feed

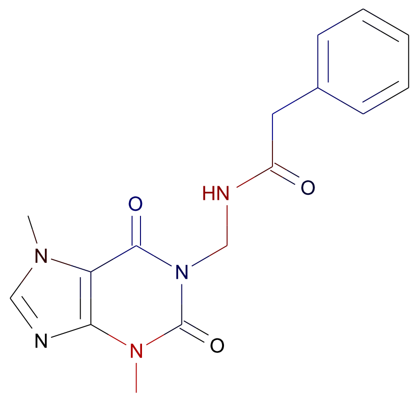

$C_{16}H_{17}N_5O_3$

Molecular Weight: 327.33788

ALogP: 0.773

Rotatable Bonds: 4

Acceptors: 4

Donors: 1

Model Prediction

Prediction: 0.0245

Unit: g/kg_body_weight

Mahalanobis Distance: 7.87

Mahalanobis Distance p-value: 0.0332

Mahalanobis Distance: The Mahalanobis distance (MD) is a generalization of the Euclidean distance that accounts for correlations among the X properties. It is calculated as the distance to the center of the training data. The larger the MD, the less trustworthy the prediction.

Mahalanobis Distance p-value: The p-value gives the fraction of training data with an MD greater than or equal to the one for the given sample, assuming normally distributed data. The smaller the p-value, the less trustworthy the prediciton. For highly non-normal X properties (e.g., fingerprints), the MD p-value is wildly inaccurate.

Structural Similar Compounds

Name	BENZIDINE,3,3'-DIMETHOXY-	NALIXIC ACID	3-NITRO-P-ACETOPHENETIDE
Structure	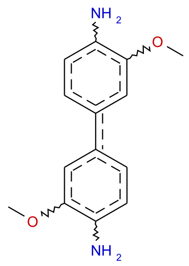	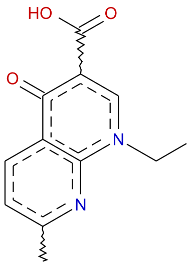	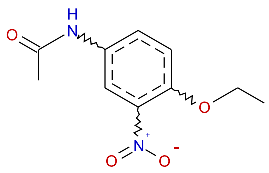
Actual Endpoint (-log C)	4.06569	3.15109	3.11767
Predicted Endpoint (-log C)	3.57405	2.99623	3.42808
Distance	0.633	0.638	0.641
Reference	NCI/NTP Report 10, Nov. 1987	NCI/NTP TR-368	NCI/NTP TR-133

Model Applicability

Unknown features are fingerprint features in the query molecule, but not found or appearing too infrequently in the training set.

1. All properties and OPS components are within expected ranges.
2. Unknown FCFP_2 feature: -306856457: [*][c]1:[*]:[*]:[cH]:n:1C
3. Unknown FCFP_2 feature: -124685461: [*]n1:[*]:[*]:n:[cH]:1
4. Unknown FCFP_2 feature: 136150461: [*]:n(:[*])C
5. Unknown FCFP_2 feature: 470101049: [*]NCN([*])[*]

Feature Contribution

Top features for positive contribution			
Fingerprint	Bit/Smiles	Feature Structure	Score
FCFP_2	136627117	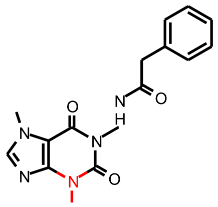 [*]N([*])C	0.173

FCFP_2	-885550502	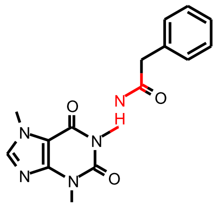 [*]CNC(=[*])[*]	0.115
FCFP_2	3	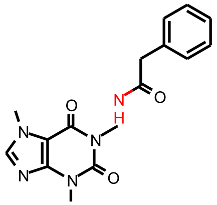 [*]N[*]	0.0737
**Top Features for negative contribution**			
**Fingerprint**	**Bit/Smiles**	**Feature Structure**	**Score**
FCFP_2	1872154524	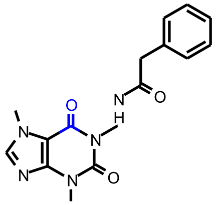 [*]C(=O)[*]	-0.105
FCFP_2	203677720	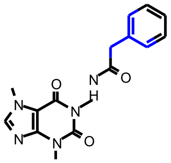 [*]C[c](:[cH]:[*]):[cH]:[*]	-0.0829
FCFP_2	1	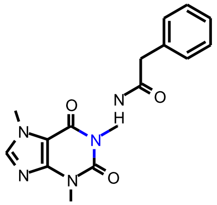 [*]N([*])[*]	-0.0796

Erlotinib

TOPKAT_Rat_Maximum_Tolerated_Dose_Feed

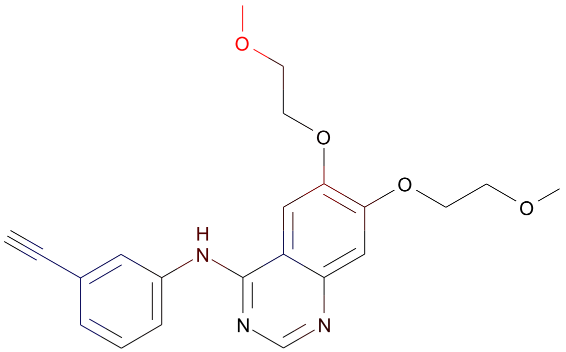

$C_{22}H_{23}N_3O_4$

Molecular Weight: 393.43572

ALogP: 4.309

Rotatable Bonds: 10

Acceptors: 7

Donors: 1

Structural Similar Compounds

Name	BUTYL BENZYL PHTHALATE	3,3'-DIMETHOXYBENZIDINE-4,4'-DIISOCYANATE	PYRILAMINE
Structure	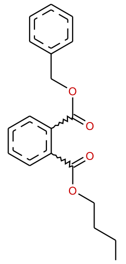	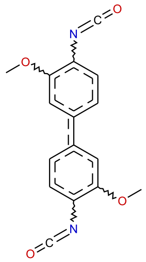	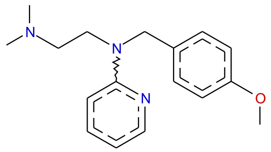
Actual Endpoint (-log C)	2.79569	2.17504	3.32511
Predicted Endpoint (-log C)	3.18498	3.78717	3.65163
Distance	0.758	0.795	0.820
Reference	NCI/NTP TR-458	NCI/NTP TR-128	NCI/NTP Report 10, Nov. 1987

Model Prediction

Prediction: 0.0828

Unit: g/kg_body_weight

Mahalanobis Distance: 9.37

Mahalanobis Distance p-value: 0.000417

Mahalanobis Distance: The Mahalanobis distance (MD) is a generalization of the Euclidean distance that accounts for correlations among the X properties. It is calculated as the distance to the center of the training data. The larger the MD, the less trustworthy the prediction.

Mahalanobis Distance p-value: The p-value gives the fraction of training data with an MD greater than or equal to the one for the given sample, assuming normally distributed data. The smaller the p-value, the less trustworthy the prediciton. For highly non-normal X properties (e.g., fingerprints), the MD p-value is wildly inaccurate.

Model Applicability

Unknown features are fingerprint features in the query molecule, but not found or appearing too infrequently in the training set.

1. All properties and OPS components are within expected ranges.
2. Unknown FCFP_2 feature: -124685461: [*]n1:[*]:[*]:n:[cH]:1
3. Unknown FCFP_2 feature: 902193919: [*]:[c](:[*])C#C
4. Unknown FCFP_2 feature: 131784192: [*]C#C

Feature Contribution

Top features for positive contribution

Fingerprint	Bit/Smiles	Feature Structure	Score
FCFP_2	136627117	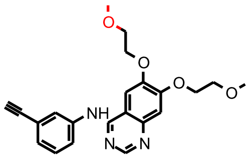 [*]N([*])C	0.173

FCFP_2	-1143715940	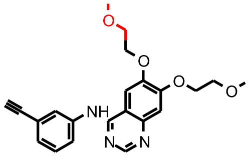 [*]COC	0.095
FCFP_2	1036089772	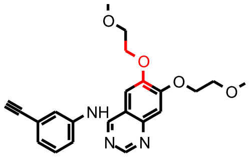 [*]CO[c](:[*]):[*]	0.0749

Top Features for negative contribution

Fingerprint	Bit/Smiles	Feature Structure	Score
FCFP_2	203677720	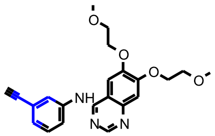 [*]C[c](:[cH]:[*]):[cH]:[*]	-0.0829
FCFP_2	1	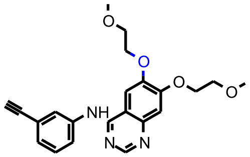 [*]N([*])[*]	-0.0796
FCFP_2	16	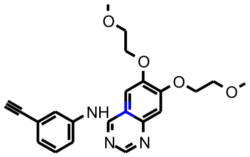 [*][c](:[*]):[*]	-0.0512

Compound 5

TOPKAT_Rat_Maximum_Tolerated_Dose_Gavage

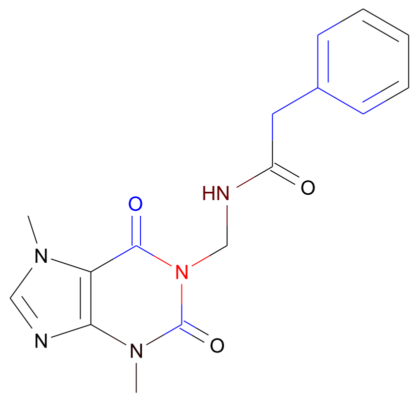

$C_{16}H_{17}N_5O_3$

Molecular Weight: 327.33788

ALogP: 0.773

Rotatable Bonds: 4

Acceptors: 4

Donors: 1

Model Prediction

Prediction: 0.2

Unit: g/kg_body_weight

Mahalanobis Distance: 8

Mahalanobis Distance p-value: 0.00209

Mahalanobis Distance: The Mahalanobis distance (MD) is a generalization of the Euclidean distance that accounts for correlations among the X properties. It is calculated as the distance to the center of the training data. The larger the MD, the less trustworthy the prediction.

Mahalanobis Distance p-value: The p-value gives the fraction of training data with an MD greater than or equal to the one for the given sample, assuming normally distributed data. The smaller the p-value, the less trustworthy the prediciton. For highly non-normal X properties (e.g., fingerprints), the MD p-value is wildly inaccurate.

Structural Similar Compounds

Name	SULFISOOXAZOLE	PROBENECID	PENICILLIN VK
Structure	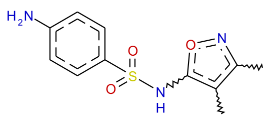	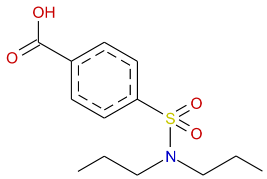	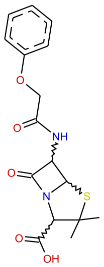
Actual Endpoint (-log C)	2.82494	2.85333	2.54455
Predicted Endpoint (-log C)	3.0705	2.4258	3.9702
Distance	0.672	0.780	0.846
Reference	NCI/NTP TR-138	NCI/NTP TR-395	NCI/NTP TR-336

Model Applicability

Unknown features are fingerprint features in the query molecule, but not found or appearing too infrequently in the training set.

1. All properties and OPS components are within expected ranges.
2. Unknown FCFP_2 feature: -1986158408: [*]N([*])C(=O)N([*])[*]
3. Unknown FCFP_2 feature: -1410049896: [*]N([*])[c]1:n:[*]:[*]:[c]:1[*]
4. Unknown FCFP_2 feature: -306856457: [*][c]1:[*]:[*]:[cH]:n:1C
5. Unknown FCFP_2 feature: -124685461: [*]n1:[*]:[*]:n:[cH]:1
6. Unknown FCFP_2 feature: 136150461: [*]:n(:[*])C
7. Unknown FCFP_2 feature: 470101049: [*]NCN([*])[*]

Feature Contribution

Top features for positive contribution

Fingerprint	Bit/Smiles	Feature Structure	Score

FCFP_2	1	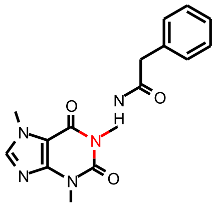 [*]N([*])[*]	0.511
FCFP_2	3	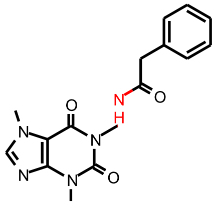 [*]N[*]	0.104
FCFP_2	136627117	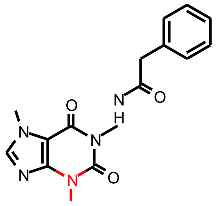 [*]N([*])C	0.0304
**Top Features for negative contribution**			
**Fingerprint**	**Bit/Smiles**	**Feature Structure**	**Score**
FCFP_2	203677720	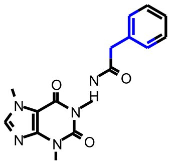 [*]C[c](:[cH]:[*]):[cH]:[*]	-0.406
FCFP_2	1872154524	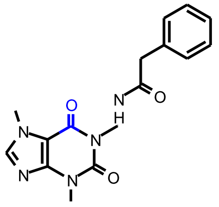 [*]C(=O)[*]	-0.307

FCFP_2	0	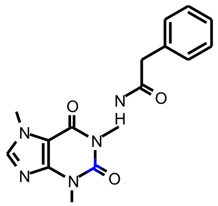 [*]C(=[*])[*]	-0.29

Erlotinib

TOPKAT_Rat_Maximum_Tolerated_Dose_Gavage

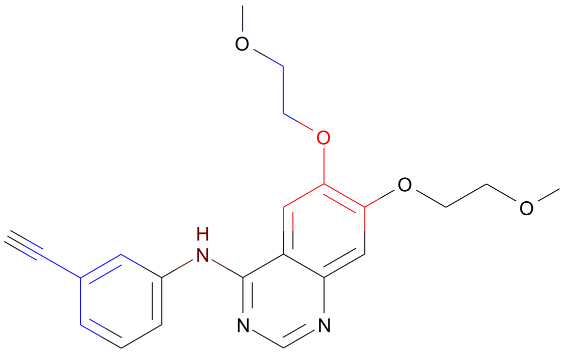

$C_{22}H_{23}N_3O_4$

Molecular Weight: 393.43572

ALogP: 4.309

Rotatable Bonds: 10

Acceptors: 7

Donors: 1

Model Prediction

Prediction: 0.000344

Unit: g/kg_body_weight

Mahalanobis Distance: 11.7

Mahalanobis Distance p-value: 2.74e-008

Mahalanobis Distance: The Mahalanobis distance (MD) is a generalization of the Euclidean distance that accounts for correlations among the X properties. It is calculated as the distance to the center of the training data. The larger the MD, the less trustworthy the prediction.
Mahalanobis Distance p-value: The p-value gives the fraction of training data with an MD greater than or equal to the one for the given sample, assuming normally distributed data. The smaller the p-value, the less trustworthy the prediciton. For highly non-normal X properties (e.g., fingerprints), the MD p-value is wildly inaccurate.

Structural Similar Compounds

Name	DIALLYL PHTHALATE	OCHRATOXIN	PROBENECID
Structure	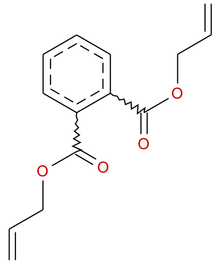	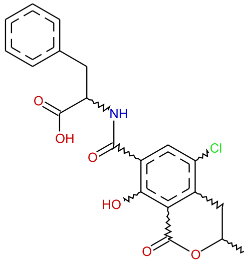	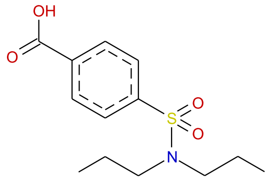
Actual Endpoint (-log C)	3.3914	6.28396	2.85333
Predicted Endpoint (-log C)	3.50093	5.12358	2.4258
Distance	1.113	1.136	1.152
Reference	NCI/NTP TR-284	NCI/NTP TR-358	NCI/NTP TR-395

Model Applicability

Unknown features are fingerprint features in the query molecule, but not found or appearing too infrequently in the training set.

1. Num_H_Acceptors out of range. Value: 7. Training min, max, mean, SD: 0, 6, 1.6146, 1.644.
2. Num_AromaticRings out of range. Value: 3. Training min, max, mean, SD: 0, 2, 0.5625, 0.693.
3. OPS PC6 out of range. Value: -3.0997. Training min, max, SD, explained variance: -2.4321, 2.9885, 1.256, 0.0488.
4. Unknown FCFP_2 feature: 1293778554: [*]:[c](:[*])N[c](:[*]):[*]
5. Unknown FCFP_2 feature: -124685461: [*]n1:[*]:[*]:n:[cH]:1
6. Unknown FCFP_2 feature: 902193919: [*]:[c](:[*])C#C
7. Unknown FCFP_2 feature: 131784192: [*]C#C

Feature Contribution

Top features for positive contribution

Fingerprint	Bit/Smiles	Feature Structure	Score

FCFP_2	332760439	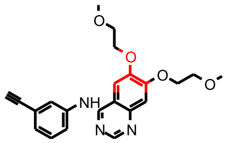 [*]O[c](:[cH]:[*]):[c]([*]):[*]	0.672
FCFP_2	1	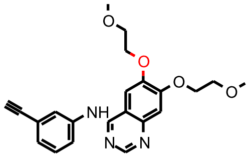 [*]N([*])[*]	0.511
FCFP_2	3	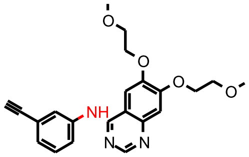 [*]N[*]	0.104

Top Features for negative contribution

Fingerprint	Bit/Smiles	Feature Structure	Score
FCFP_2	203677720	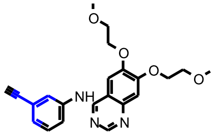 [*]C[c](:[cH]:[*]):[c H]:[*]	-0.406
FCFP_2	0	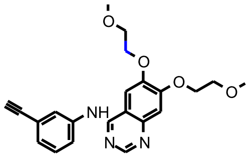 [*]C(=[*])[*]	-0.29

FCFP_2	-1272768868	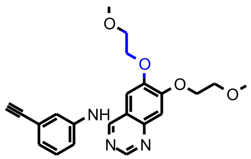 [*]CCO[*]	-0.271

Compound 5

TOPKAT_Rat_Oral_LD50

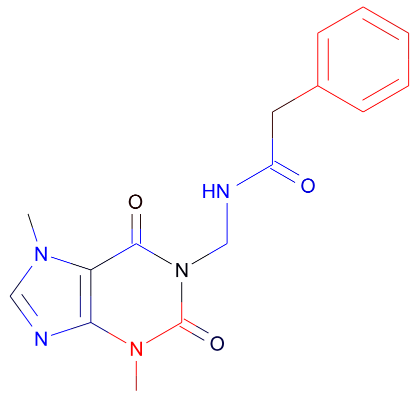

$C_{16}H_{17}N_5O_3$
Molecular Weight: 327.33788
ALogP: 0.773
Rotatable Bonds: 4
Acceptors: 4
Donors: 1

Structural Similar Compounds

Name	PENTOXYFYLLINE	NITRAZEPAM	PROXYPHYLLINE
Structure	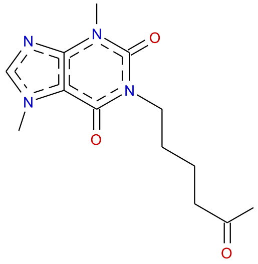	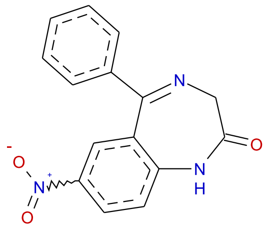	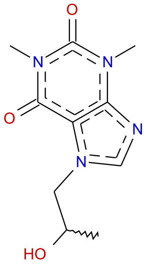
Actual Endpoint (-log C)	2.376	2.533	2.714
Predicted Endpoint (-log C)	2.23193	1.97908	2.38533
Distance	0.527	0.571	0.576
Reference	YACHDS 9;13;81	TXAPA9 18;185;71	OYYAA2 19;845;80

Model Prediction

Prediction: 1.58
Unit: g/kg_body_weight
Mahalanobis Distance: 21.8
Mahalanobis Distance p-value: 1.21e-015

Mahalanobis Distance: The Mahalanobis distance (MD) is a generalization of the Euclidean distance that accounts for correlations among the X properties. It is calculated as the distance to the center of the training data. The larger the MD, the less trustworthy the prediction.
Mahalanobis Distance p-value: The p-value gives the fraction of training data with an MD greater than or equal to the one for the given sample, assuming normally distributed data. The smaller the p-value, the less trustworthy the prediciton. For highly non-normal X properties (e.g., fingerprints), the MD p-value is wildly inaccurate.

Model Applicability

Unknown features are fingerprint features in the query molecule, but not found or appearing too infrequently in the training set.

1. All properties and OPS components are within expected ranges.
2. Unknown ECFP_2 feature: -39630146: [*]NCN([*])[*]
3. Unknown FCFP_6 feature: 16: [*][c](:[*]):[*]
4. Unknown FCFP_6 feature: -1410049896: [*]N([*])[c]1:n:[*]:[*]:[c]:1[*]
5. Unknown FCFP_6 feature: -306856457: [*][c]1:[*]:[*]:[cH]:n:1C
6. Unknown FCFP_6 feature: -124685461: [*]n1:[*]:[*]:n:[cH]:1
7. Unknown FCFP_6 feature: 1747237384: [*][c]1:[*]:[*]:[cH]:n:1
8. Unknown FCFP_6 feature: 136150461: [*]:n(:[*])C
9. Unknown FCFP_6 feature: 470101049: [*]NCN([*])[*]
10. Unknown FCFP_6 feature: 1618154665: [*][c](:[*]):[cH]:[cH]:[*]

Feature Contribution

Top features for positive contribution			
**Fingerprint**	**Bit/Smiles**	**Feature Structure**	**Score**

ECFP_6	642810091	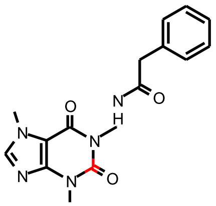<br>[*]C(=[*])[*]	0.281
ECFP_6	-1897341097	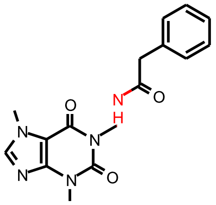<br>[*]N[*]	0.216
ECFP_6	1571214559	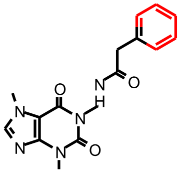<br>[*]1:[cH]:[cH]:[cH]:[cH]:[cH]:1	0.19
**Top Features for negative contribution**			
**Fingerprint**	**Bit/Smiles**	**Feature Structure**	**Score**
ECFP_6	2106656448	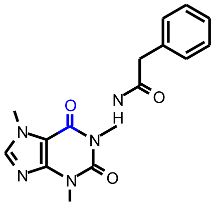<br>[*]C(=O)[*]	-0.352
ECFP_6	497523368	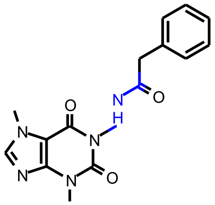<br>[*]CNC(=[*])[*]	-0.301

ECFP_6	655739385	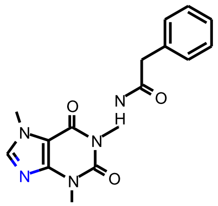<br>[*]:n:[*]	-0.239

Erlotinib

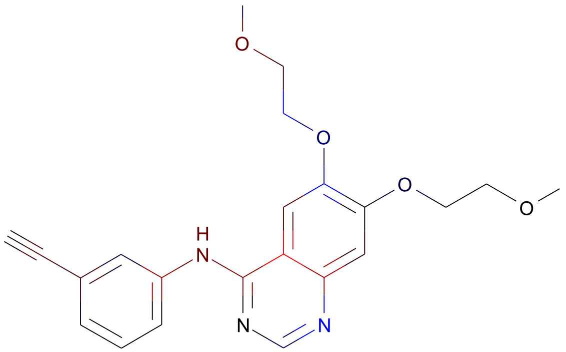

$C_{22}H_{23}N_3O_4$
Molecular Weight: 393.43572
ALogP: 4.309
Rotatable Bonds: 10
Acceptors: 7
Donors: 1

Model Prediction

Prediction: 0.662
Unit: g/kg_body_weight
Mahalanobis Distance: 20.8
Mahalanobis Distance p-value: 9.57e-012

Mahalanobis Distance: The Mahalanobis distance (MD) is a generalization of the Euclidean distance that accounts for correlations among the X properties. It is calculated as the distance to the center of the training data. The larger the MD, the less trustworthy the prediction.
Mahalanobis Distance p-value: The p-value gives the fraction of training data with an MD greater than or equal to the one for the given sample, assuming normally distributed data. The smaller the p-value, the less trustworthy the prediciton. For highly non-normal X properties (e.g., fingerprints), the MD p-value is wildly inaccurate.

Structural Similar Compounds

Name	TALNIFLUMATE	3-QUINOLINECARBOXYLIC ACID; 6;7-bis-(CYCLOPROPYLMETHOXY)-4-HYDROXY-; ETHYL ESTER	DIXYRAZINE .HCl (HCl STRIPPED)
Structure	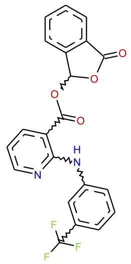	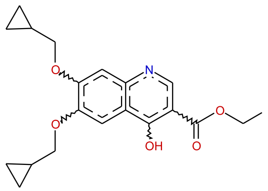	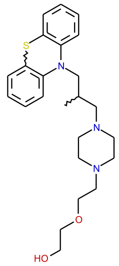
Actual Endpoint (-log C)	1.538	2.076	3.029
Predicted Endpoint (-log C)	2.82541	2.50101	2.47585
Distance	0.615	0.615	0.654
Reference	FRPSAX 36;372;81	TXAPA9 18;185;71	ANPBAZ 61;669;61

Model Applicability

Unknown features are fingerprint features in the query molecule, but not found or appearing too infrequently in the training set.

1. All properties and OPS components are within expected ranges.
2. Unknown ECFP_2 feature: 1139738044: [*]:[c](:[*])C#C
3. Unknown FCFP_6 feature: 16: [*][c](:[*]):[*]
4. Unknown FCFP_6 feature: 1293778554: [*]:[c](:[*])N[c](:[*]):[*]
5. Unknown FCFP_6 feature: 1747237384: [*][c]1:[*]:[*]:[cH]:n:1
6. Unknown FCFP_6 feature: 1618154665: [*][c](:[*]):[cH]:[cH]:[*]
7. Unknown FCFP_6 feature: -1151884458: [*]N[c](:n:[*]):[c](:[*]):[*]
8. Unknown FCFP_6 feature: -124685461: [*]n1:[*]:[*]:n:[cH]:1
9. Unknown FCFP_6 feature: 902193919: [*]:[c](:[*])C#C
10. Unknown FCFP_6 feature: 131784192: [*]C#C

Feature Contribution

Top features for positive contribution

Fingerprint	Bit/Smiles	Feature Structure	Score

ECFP_6	642810091	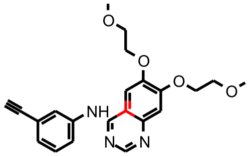 [*]C(=[*])[*]	0.281
ECFP_6	-1897341097	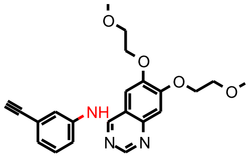 [*]N[*]	0.216
FCFP_6	136627117	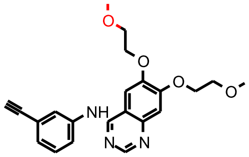 [*]N([*])C	0.17
**Top Features for negative contribution**			
**Fingerprint**	**Bit/Smiles**	**Feature Structure**	**Score**
ECFP_6	655739385	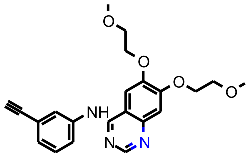 [*]:n:[*]	-0.239
ECFP_6	734603939	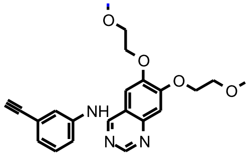 [*]C	-0.201

FCFP_6	1036089772	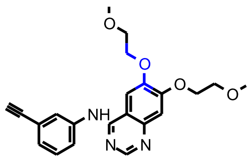 [*]CO[c](:[*]):[*]	-0.136
